# Spatial transcriptomics reveals a key role of fibroblast-like vascular smooth muscle cells in human atherosclerotic cell crosstalk and stability

**DOI:** 10.1093/eurheartj/ehaf1091

**Published:** 2026-02-13

**Authors:** Isabel Goncalves, Mengyu Pan, Pratibha Singh, Wenqi Wang, Jing Zhao, Lea Dib, Lena Sundius, Ana Persson, Chrysostomi Gialeli, Panagiotis Fountas, Mihaela Nitulescu, Jan Nilsson, Stephen Malin, Claudia Monaco, Helle F Jørgensen, Jiangming Sun, Andreas Edsfeldt

**Affiliations:** Cardiovascular Research–Translational Studies, Clinical Sciences Malmö, Lund University, Jan Waldenströms gata 35, SE-214 28, Malmö, Sweden; Department of Cardiology, University Hospital of Skåne, Jan Waldenströms gata 15, SE-214 28, Malmö, Sweden; Cardiovascular Research–Translational Studies, Clinical Sciences Malmö, Lund University, Jan Waldenströms gata 35, SE-214 28, Malmö, Sweden; Cardiovascular Research–Translational Studies, Clinical Sciences Malmö, Lund University, Jan Waldenströms gata 35, SE-214 28, Malmö, Sweden; Cardiovascular Research–Translational Studies, Clinical Sciences Malmö, Lund University, Jan Waldenströms gata 35, SE-214 28, Malmö, Sweden; Section of Cardiorespiratory Medicine, University of Cambridge, VPD Heart and Lung Research Institute, Papworth Road, Cambridge Biomedical Campus, Cambridge CB2 0BB, UK; Kennedy Institute of Rheumatology, Nuffield Department of Orthopaedics, Rheumatology and Musculoskeletal Sciences, University of Oxford, Oxford, UK; School of Biological Sciences, University of Reading, Berkshire, UK; Cardiovascular Research–Translational Studies, Clinical Sciences Malmö, Lund University, Jan Waldenströms gata 35, SE-214 28, Malmö, Sweden; Cardiovascular Research–Translational Studies, Clinical Sciences Malmö, Lund University, Jan Waldenströms gata 35, SE-214 28, Malmö, Sweden; Cardiovascular Research–Translational Studies, Clinical Sciences Malmö, Lund University, Jan Waldenströms gata 35, SE-214 28, Malmö, Sweden; Cardiovascular Research–Translational Studies, Clinical Sciences Malmö, Lund University, Jan Waldenströms gata 35, SE-214 28, Malmö, Sweden; Cardiovascular Research–Translational Studies, Clinical Sciences Malmö, Lund University, Jan Waldenströms gata 35, SE-214 28, Malmö, Sweden; Cardiovascular Research–Translational Studies, Clinical Sciences Malmö, Lund University, Jan Waldenströms gata 35, SE-214 28, Malmö, Sweden; Department of Medicine Solna, Karolinska Institute, Stockholm, Sweden; Kennedy Institute of Rheumatology, Nuffield Department of Orthopaedics, Rheumatology and Musculoskeletal Sciences, University of Oxford, Oxford, UK; Section of Cardiorespiratory Medicine, University of Cambridge, VPD Heart and Lung Research Institute, Papworth Road, Cambridge Biomedical Campus, Cambridge CB2 0BB, UK; Cardiovascular Research–Translational Studies, Clinical Sciences Malmö, Lund University, Jan Waldenströms gata 35, SE-214 28, Malmö, Sweden; Cardiovascular Research–Translational Studies, Clinical Sciences Malmö, Lund University, Jan Waldenströms gata 35, SE-214 28, Malmö, Sweden; Department of Cardiology, University Hospital of Skåne, Jan Waldenströms gata 15, SE-214 28, Malmö, Sweden; Wallenberg Centre for Molecular Medicine, Lund University, Lund, Sweden

**Keywords:** Atherosclerosis, Carotid plaque, RNA sequencing, Spatial transcriptomics, Human, Cell interactions

## Abstract

**Background and Aims:**

Atherosclerotic plaques are the leading cause of cardiovascular events. Single-cell approaches have identified diverse human plaque cell phenotypes but their spatial distribution and interactions remain unclear. Here, intercellular communication patterns in human plaque microenvironments were mapped to reveal novel targets to prevent atherosclerotic events.

**Methods:**

Spatial transcriptomics (Visium, 10x) from 13 carotid plaques, and single-cell transcriptomics (cells = 51 981) were used to analyse cell phenotypes, cell trajectories, and intercellular communications. Cells contributing to plaque stability were explored using deconvolution of plaque bulk RNA-seq data (*n* = 78), histology, and survival analyses. Key cells and pathways were validated in apolipoprotein E (*Apoe*)^−/−^ mice and *in vitro*. Genome-wide association study enrichment analyses were conducted using summary statistics of atherosclerotic diseases. LINCS L1000 data were used to explore drug repurposing.

**Results:**

A fibroblast-like vascular smooth muscle cell (VSMC) phenotype associated with extracellular matrix formation pathways (validated in *Apoe^−/−^* mice) emerged as a key regulator of intra-plaque ligand-receptor signalling, in particular in the cap region. A higher proportion of fibroblast-like VSMCs was found in asymptomatics, associated with stable plaque features and predicted a lower risk of future events. Genes specific to this VSMC phenotype were enriched in coronary artery disease and myocardial infarction. Finally, compounds, which could induce key marker genes were identified and validated *in vitro*.

**Conclusions:**

This study provides the first comprehensive spatial transcriptomics map of cell communication in human plaque microenvironments. A pivotal role of a fibroblast-like VSMC, orchestrating intraplaque cell signalling and contributing to plaque stability, was identified. Targeting these cells might present promising novel avenues for therapies.

## Graphical Abstract

**Structured Graphical abstract:**
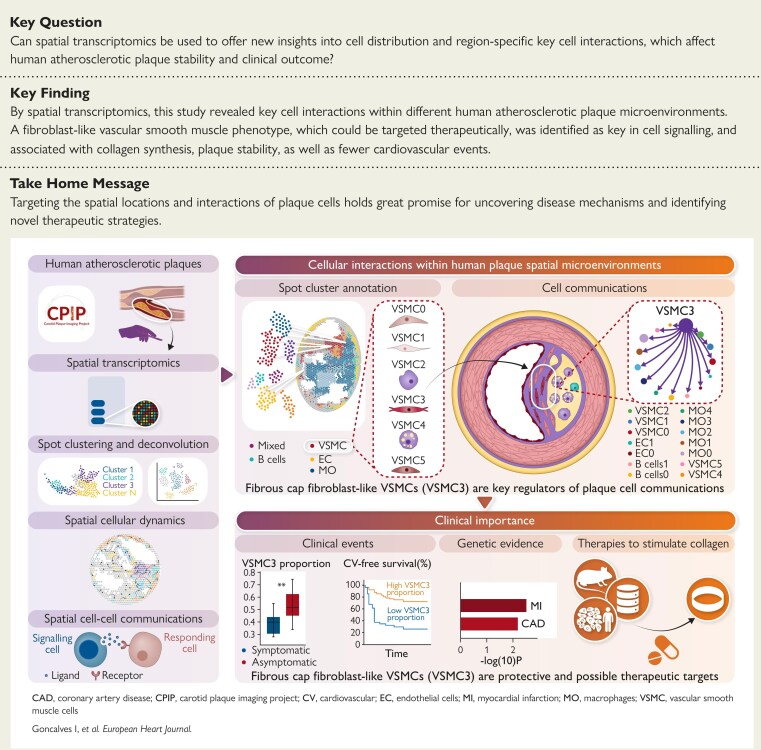

**See the editorial comment for this article ‘Spatial mapping of stability in human atherosclerosis for the next generation of patient stratification’, by G. Pasterkamp**  ***et al*****., https://doi.org/10.1093/eurheartj/ehag074.**

Translational perspective Atherosclerotic plaques are the leading cause of cardiovascular events. While transcriptomic advances have uncovered diverse plaque cell phenotypes, their location and interactions within the plaque microenvironments remain unknown.

Performing spatial transcriptomics of human plaques, key ligand–receptor signalling pathways among the cell types in the various geographic plaque regions were revealed. Notably, a fibroblast-like vascular smooth muscle cell emerges as a key regulator of plaque cell signalling, especially in the cap, associated with a stable plaque phenotype and less events in the follow up. This suggests that targeting these specific cell interactions may become promising targets to modulate human atherosclerosis.

## Introduction

Atherosclerosis is a leading cause of both cardiovascular (CV) and cerebrovascular events. Several biological processes, including inflammation, lipid accumulation, and apoptosis, contribute to disease progression and its acute complications.^1^ Targeting these processes locally, ideally within the specific microenvironments or regions within the atherosclerotic plaques, could provide an efficient strategy to prevent disease progression and plaque rupture-related complications.^2^ However, current treatment approaches focus on systemic risk factors rather than local plaque-specific mechanisms.

Clinical trials have demonstrated CV benefits by reducing inflammatory activity.^3^ Unfortunately, these studies also reported an increased risk of non-CV mortality, likely due to systemic effects that extend beyond local plaque inflammation. This highlights the need for a localized perspective on the pathophysiological mechanisms within plaques. Spatial transcriptomics has emerged as a powerful tool for achieving this spatial or ‘geographic’ understanding of plaque biology.^4–8^

In recent years, single-cell RNA sequencing (scRNA-seq) has revolutionized the knowledge of the transcriptional and cellular landscape in human atherosclerosis. This technique has led to major discoveries, including the identification of macrophage subtypes, the role of clonal cell expansion, and the contributions of specific cell populations to repair responses.^7,9,10^ However, despite these advances, single-cell approaches are inherently limited by their requirement for tissue dissociation, which destroys the spatial context of cells and may result in the loss of key cell phenotypes. These results in the loss of crucial ‘geographic’ information about the cellular microenvironment, including interactions with neighbouring cells and surrounding signalling molecules. Spatial transcriptomics addresses this limitation by preserving spatial context, enabling a thorough understanding of how cells interact, differentiate and communicate within their native environment. To uncover novel therapeutic targets, it is essential to integrate cell phenotypes with their spatial organization in atherosclerotic plaques. This approach allows us to explore how the local microenviroments influence cell-cell communication.

In this study, we aimed to characterize the spatial landscape of cellular communication within advanced human atherosclerotic plaques, focusing on histologically-defined regions crucial to plaque vulnerability. By integrating spatial sequencing and scRNA-seq, we provide a comprehensive map of cell-specific ligand-receptor interactions. In particular, we discovered that a specific phenotype of vascular smooth muscle cells (VSMCs) is central to plaque cell signalling and associated with extracellular matrix formation. Using validation datasets, bulk RNA data, public genome-wide association studies (GWAS), a mouse model and *in vitro* validation, we show that this fibrous cap-associated VSMC phenotype contributes to a stable plaque phenotype with a lower risk of future clinical events (*Structured Graphical Abstract*).

## Methods

The detailed methods are described in supplementary materials. Carotid plaques from the Carotid Plaque Imaging Project (ClinicalTrials.gov: ID NCT05821894) biobank were used.^7^ Clinical characteristics of the cohort are summarized in *Table 1*. The study was approved by the Swedish ethical committee (472/2005, 2014/904, 2017/89, 2018/63, 27-2020/3.1, 60/2008, 2012/209, 2023-05910-01). Written consent was provided by all participants, and the study follows the Declaration of Helsinki.

**Table 1:** Clinical characteristics of the 13 patients whose carotid plaques were included in the spatial transcriptome analysis

Clinical Characteristics	
Age, years	70 (64–79)
Male sex	11 (84.6)
Degree of stenosis, %	85 (80–95)
Symptoms	8 (61.5)
Type 2 diabetes	6 (46.2)
Hypertension	10 (77)
Current smokers	2 (15.3)
Family history	7 (53.8)
Body mass index, kg/m^2^	27.7 (25.3–30.6)
hsCRP, mg/L	4 (2.4–9.3)
Fasting lipoproteins	
Cholesterol, mmol/L	3.9 (2.8–4.9)
LDL, mmol/L	2.0 (1.5–3.2)
HDL, mmol/L	1.1 (1.0–1.5)
Triglycerides, mmol/L	1.2 (0.9–1.8)
Statin use	12 (92.3)

Continuous variables are presented as median (IQR), while categorical variables are summarized as *n* (%).

BMI, body mass index; HDL, high-density lipoprotein; hsCRP, high-sensitivity C-reactive protein; IQR, interquartile range; LDL , low-density lipoprotein.

### Spatial sequencing analysis

Briefly, spatial transcriptomics was performed on sections from 13 human carotid plaques using the Visium Spatial Gene Expression Slide & Reagent Kit, 16 reactions (Catalog #PN-1000184) following the manufacturer’s protocol (CG000239 RevD,10x Genomics, Pleasanton, CA, USA) and sequenced by NextSeq 500/550. The clustering was performed using Seurat.^11^ Previous scRNA-seq data^12^ were used as reference for cell type deconvolution. RNA velocity analyses were performed on cell subclusters.

Intercellular communications within subclusters and plaque regions were analysed using CellChat^13^ (version 2.1.2), within a contact range of 100 µm and an interaction range of 250 µm. The ligand-receptor interaction database used in this analsyis included 2239 interactions, excluding those involving non-protein signalling pairs.^13^ Cell communication differences comparing plaque regions (based on haematoxylin and eosin [H&E] staining) and presence of symptoms were examined.

### Plaque histological vulnerability index

Plaque sections were stained for α-smooth muscle actin (α-SMA), Oil Red O, CD68, glycophorin A and Russell–Movat pentachrome to generate the histological vulnerability index.^14^

### Deconvolution of bulk RNA sequencing of human carotid plaques

RNA was isolated from the most stenotic region from 78 carotid plaques (51 symptomatic) and sequenced using Illumina HiSeq2000 and NextSeq 500 platforms.^15,16^ Using the obtained 19 spot clusters from the spatial RNA-seq analysis of human carotid plaques as references, Dtangle^17^ was applied for spot cluster deconvolution, estimating spot cluster composition.

### Clinical follow-up

Information regarding postoperative CV events (2005–2015) was collected through the Swedish National In-patient Health Register and the National Cause of Death Register.^18^

### *In vitro* models of fibroblast-like VSMC signalling

Immortalized human coronary artery VSMCs were derived from primary SMCs^19^ and treated with transforming growth factor-β1 (TGF-β1; 24 h) and platelet-derived growth factor-BB (PDGF-BB; 48 h) to induce a fibroblast-like phenotype. For validation, CD44 signalling was blocked using CD44 antibodies (ab254530, Abcam), and candidate drugs were added to the culture medium. Potential effects were studied using qPCR for gene expression differences and Sirius Red for collagen production.

### Validation of fibroblast-like VSMC markers and CD44-COL 1 interaction in human carotid plaques

Multiplex immunofluorescence staining on human plaque tissue section was performed using a sequential protocol based on Phenoptics workflow (Akoya Biosciences) and imaged using PhenoImager HT (Akoya Biosciences). Proximity ligation assay (PLA) was performed on FFPE tissue sections using the Duolink® *in situ* red starter kit (Sigma-Aldrich, DUO92101) to validate CD44-COL1 interaction, following the manufacturer’s protocol.

### Genome-wide gene-based association and gene-set analyses

To determine whether fibroblast-like VSMCs (VSMC3) were implicated in large artery stroke (LAS), coronary artery disease (CAD), or myocardial infarction (MI), genome-wide gene-based associations and gene-set analyses were conducted using MAGMA.^20^

### Validation of human fibroblast-like VSMC in Apoe^−/−^ mice

Publicly available single-cell RNA-seq profiles of lineage-labelled VSMCs from healthy *Apoe^+/+^* mice and high-fat-diet feeding *Apoe^−/−^* mice were used to test for the presence of an equivalent fibroblast-like VSMC phenotype in mice.^21^

### Validation of fibroblast-like VSMC in carotid and coronary atherosclerosis

An integrated single-cell dataset of plaque cells from human carotid and coronary plaques was used to validate the presence of fibroblast-like VSMCs.^22^

### Candidate drug screening

Overlapping genes highly expressed in human fibroblast-like VSMCs (VSMC3) and the phenotypically similar fibrous cap-associated VSMCs from *Apoe^−/−^* mice were used to explore LINCS L1000 chemical perturbation consensus signatures^23^ through Enrichr^24^ aiming to identify candidate drugs for possible repurposing in the context of atherosclerosis. A single-sample gene set enrichment analysis was performed to create a drug score for bulk RNA sequencing data from human carotid plaques (*n* = 78), using the LINCS L1000 chemical perturbation consensus signatures. A high drug score indicated strong drug activity.

## Results

### Cell type composition in plaques

**Figure 1:**
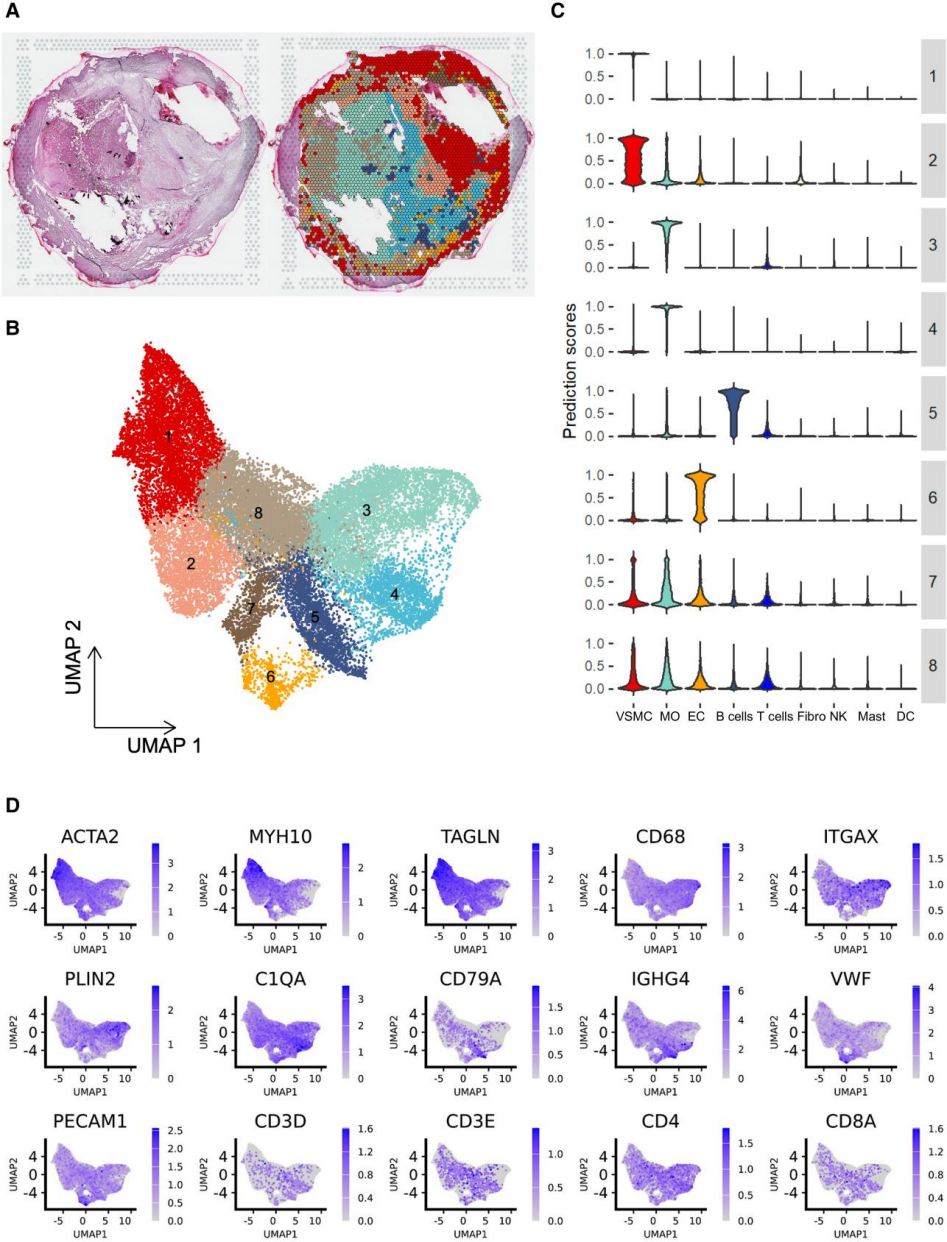
Human atherosclerotic plaque cell composition. (*A*) Left: haematoxylin and eosin staining of a representative plaque tissue section. Right: Spatial mapping of the 8 major clusters on a representative plaque tissue section. (*B*) Uniform Manifold Approximation and Projection visualizing the 8 main clusters identified in the human plaques by spatial transcriptomics. The spatial transcriptomics data contains 25 618 spots from 13 human carotid plaques. (*C*) Cell type deconvolution of the human plaque spatial transcriptomics data. The violin plots show prediction scores for each cell type (based on reference data) across the spot clusters. VSMC: vascular smooth muscle cells, MO: macrophages, EC: endothelial cells, Fibro: fibroblasts, NK:natural killer cells, Mast: mast cells, DC: dendritic cells. (*D*) Uniform manifold approximation and projections visualizing the expression of common marker genes for smooth muscle cells (*ACTA2, MYH10, TAGLN*), macrophages (*CD68, ITGAX, PLIN2, C1QA*), B-cells (*CD79A, IGHG4*), endothelial cells (*VWF, PECAM1*) and T-cells (CD3D, CD3E, CD4 and CD8A).

To obtain an overview of the plaque ‘geographic’ regions, H&E staining (*Figure 1A*) was performed on 13 human carotid plaques and, then in detail, spatial transcriptomic profiling was done, yielding a total of 25 618 sequencing spots, with a median of 14 548 genes (14 128–15 813) detected per plaque. Eight distinct spatial clusters were identified (*Figure 1A and 1B*), from which the cellular compositions were inferred using scRNA-seq data from human atherosclerotic plaques as reference.^12^ VSMC were predominant in clusters 1 and 2, whereas macrophages dominated clusters 3 and 4. Clusters 5 and 6 were primarily composed of B-cells and endothelial cells (ECs), respectively. Clusters 7 and 8 exhibited a mixed cellular composition where T-cells, macrophages, dendritic cells (DCs), and VSMCs were observed (*Figure 1C*). Overall, the expression of common T-cell markers was rather low (*Figure 1D*).

In line with the inferred cellular compositions, the VSMC markers *ACTA2, MYH10*, and *TAGLN* were highly expressed in clusters 1 and 2 and the macrophage markers—*CD68*, *ITGAX*, *PLIN2*, and *C1QA* were highly expressed in clusters 3 and 4 (*Figure 1D*). Additionally, B-cell markers (*CD79A*, *IGHG4*) and EC markers (*VWF*, *PECAM1*) were predominantly expressed in clusters 5 and 6, respectively (*Figure 1D*).

### Characterization and location of VSMC subsets

**Figure 2:**
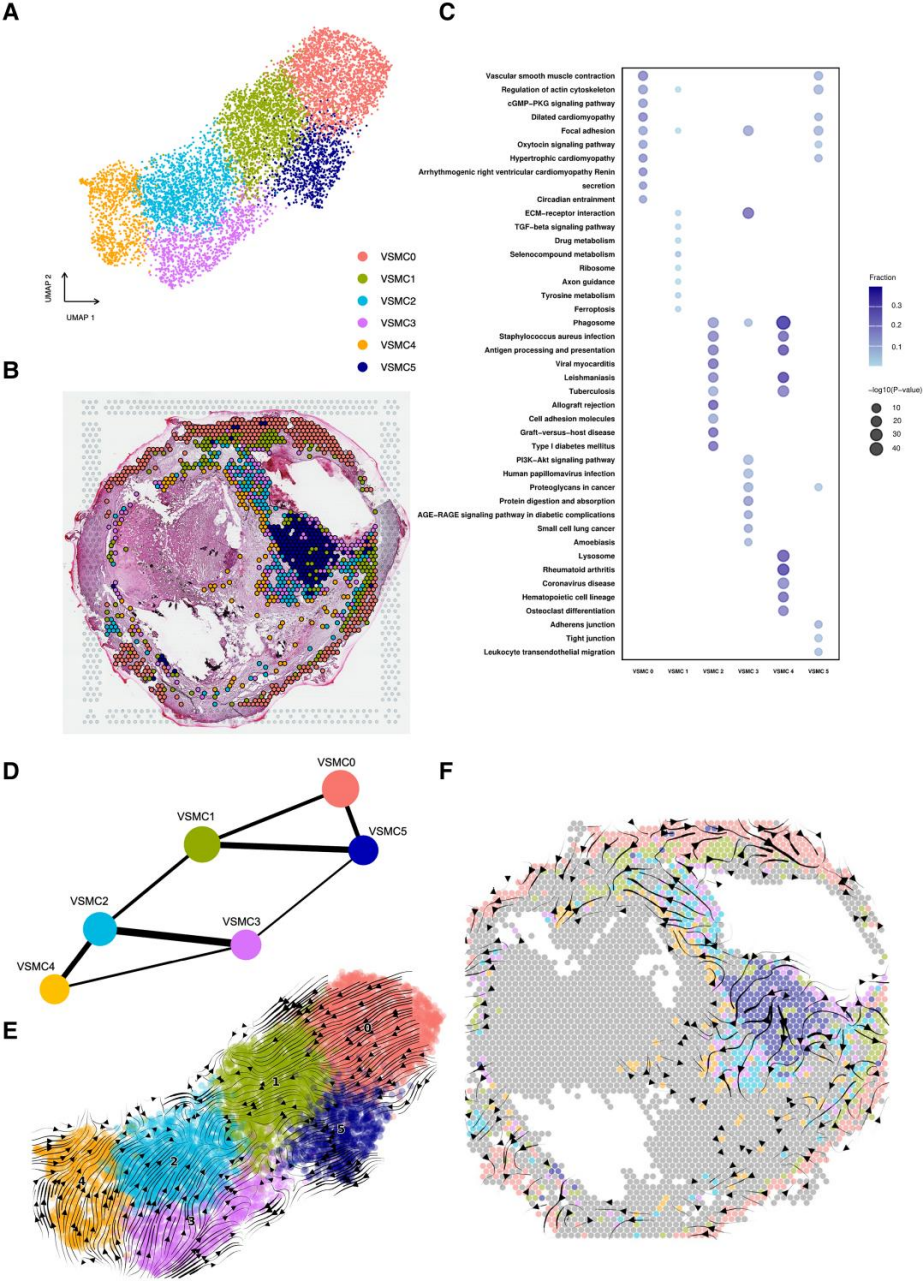
Vascular smooth muscle cells subtypes identified by spatial transcriptomics analysis of human atherosclerotic plaques. (*A*) Uniform manifold approximation and projection visualizing the six identified subclusters of vascular smooth muscle cells (VSMC), based on 8, 093 spots from 13 human carotid plaques. Six VSMC subclusters were annotated: contractile medial (VSMC0), transitional contractile (VSMC1) and transitional contractile (VSMC5), fibroblast-like (VSMC3), macrophages-foam-cell like (VSMC4) and macrophages-like VSMC (VSMC2). (*B*) Spatial mapping of the six VSMC subclusters on a representative human plaque section. (*C*) Heatmap showing the top 10 enriched pathways by *P*-value for each VSMC subcluster, identified using highly expressed genes per cluster. (*D*) Partition-based graph abstraction (PAGA) graph showing connectivity among the six VSMC subclusters, with line thickness indicating connection strength. (*E*) RNA velocity estimated cellular state transitions between VSMC subclusters. Left: stream plot of RNA velocities overlaid on a Uniform Manifold Approximation and Projection plot. (*F*) Right: RNA velocity stream mapping to spatial location on a plaque tissue section

To provide a higher resolution of the predicted human plaque VSMC spots, a sub-clustering analysis was performed on VSMC-predicted clusters 1 and 2 spots (*Figure 1B*). Six VSMC subclusters (*Figure 2A*) and, more importantly, their locations within the plaque tissue sections were demonstrated (*Figure 2B*). Interestingly, clusters showed spatially distinct distributions (*Figure 2B*), suggesting that the VSMC states differ in the various microenvironments.

Highly expressed differential expressed genes (DEGs) in the six subclusters and the enriched pathways (*Figure 2C*) were examined (Supplementary data online, *Table S1*). Cell phenotypes for each VSMC subcluster were annotated based on DEGs and described VSMC markers. This way, three clusters of contractile VSMCs were detected: medial VSMCs (VSMC0; *MYH11^high^, MYH10^high^, CNN1^high^, CALD1^high^ and PLN^high^*; Supplementary data online, *Figure S1A*-*C*), transitional contractile VSMC type 1 with a more synthetic transcriptional signature (VSMC1; *ACTA2^low^*, *CNN1^low^* compared to VSMC5, Supplementary data online, *Figure S1D*; *COL5A1^high^, COL3A1^high^,* and *LUM^high^* compared to VSMC0, Supplementary data online, *Figure S1E*) and transitional contractile VSMC type 2 (VSMC5; *ACTA2^high^, CNN1^high^* and *MYH11^high^; CALD1^low^, PLN^low^* and *MYH10^low^* comparing with VSMC0, Supplementary data online, *Figure S1F*). We also identified one cluster of fibroblast-like VSMCs (VSMC3; *FN1^high^, LUM^high^*, *DCN^high^,* and *MMP2^high^,*  Supplementary data online, *Figure S1A*–*C*), one cluster of macrophage like VSMCs (VSMC2; *CTSB^high^, SPP1^high^, CD14^high^, CD163^high^ ACTA2^l^*^ow^ and *MYH10^low^* comparing with VSMC1; Supplementary data online, *Figure S1G*) and one cluster of macrophage-foam-cell like VSMCs (VSMC4; *CD36^high^, LPL^high^, APOE^high^, SPP1^high^, CD14^high^, CD163^high^*, and *S100A9^high^*; Supplementary data online, *Figure S1A* and *B*). Enriched pathways in each cluster are shown in *Figure 2C*.

To assess the connectivity between VSMC subclusters, a partition-based graph abstraction (PAGA) graph was constructed. Strong connections were observed between VSMC0, VSMC1, and VSMC5, as well as between VSMC2, VSMC3 and VSMC4 (*Figure 2D*). To further investigate the potential cell-state transitions among VSMC subclusters, a RNA velocity analysis was performed. The directions of RNA velocity indicated apparent transitions from VSMC3 and VSMC4 towards VSMC2, as well as VSMC0 towards VSMC1, and subsequently to VSMC2 (*Figure 2E*). Transitions from VSMC0 to VSMC5, VSMC5 to VSMC1, VSMC5 to VSMC3, and VSMC3 to VSMC1 were also identified (*Figure 2E*). These transitions were also observed within plaque microenvironments (*Figure 2F*)

### Characterisation and location of macrophage subsets

**Figure 3:**
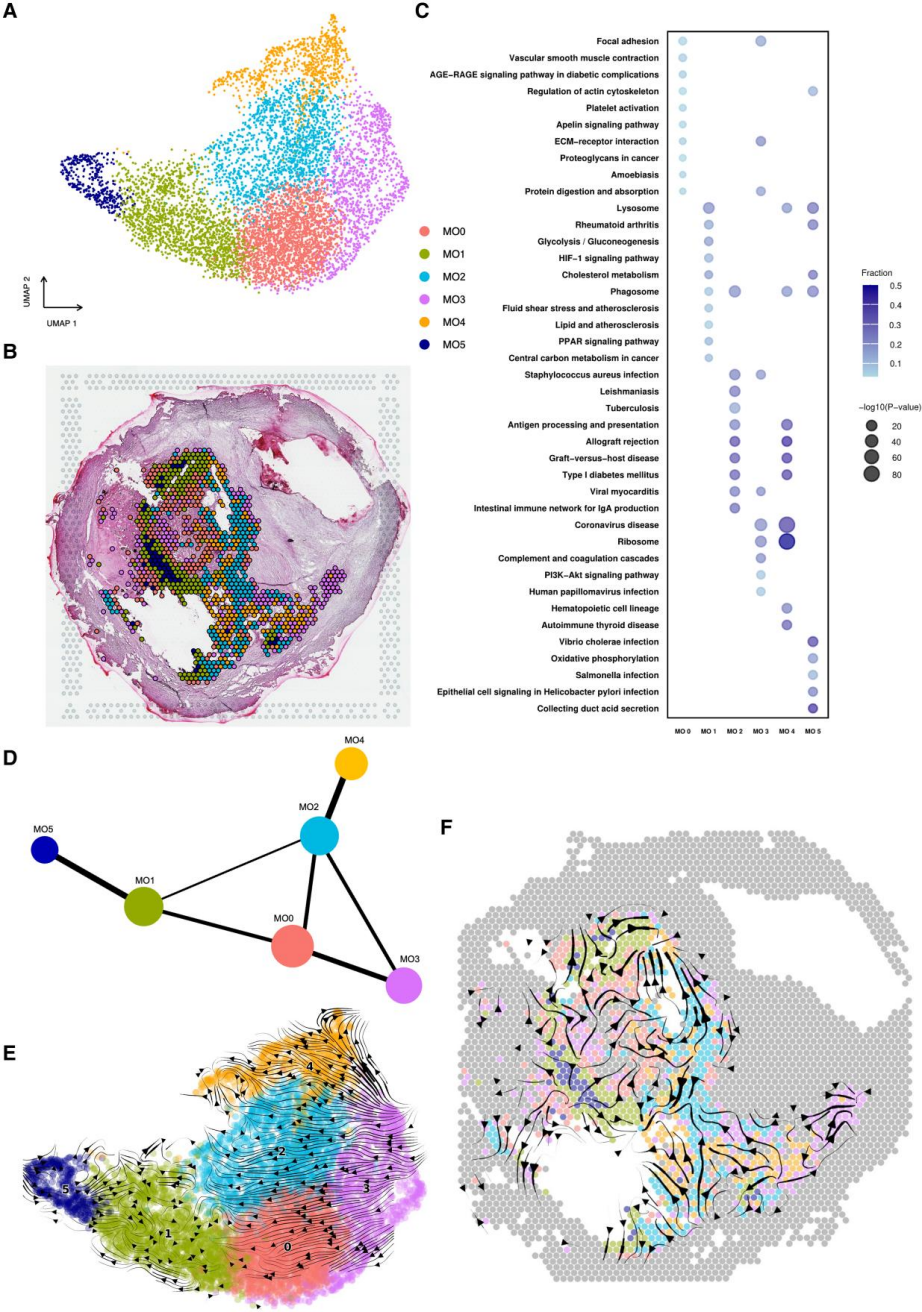
Macrophage subtypes identified by spatial transcriptomics analysis of human atherosclerotic plaques. (*A*) Uniform manifold approximation and projection plot visualizing the six identified macrophage (MO) subclusters in 7472 spots from 13 human carotid plaques. Six MO subclusters were annotated: MO1: *TREM1*^high^*PLIN2*^high^; MO2: resident; MO3 and MO0: synthetic/vascular smooth muscle cell like (VSMC-like); MO4: resident; MO5: *TREM2*^high^. (*B*) Spatial distribution of the six macrophage subclusters on a representative plaque tissue section. (*C*) Top 10 enriched pathways by *P*-value for each macrophage subcluster, identified using highly expressed genes per cluster. (*D*) Partition**-**based graph abstraction (PAGA) graph illustrating connectivity between macrophage subclusters, with line thickness indicating connection strength. (*E*) RNA velocity estimated cellular transcriptional state transitions between macrophage subclusters. Left: stream plot of RNA velocities overlaid on a uniform manifold approximation and projection plot. (*F*) Right: RNA velocity stream mapping to spatial location on a plaque tissue section.

A subclustering analysis was performed on all spots from the macrophage-predicted clusters (clusters 3 and 4 in *Figure 1B*), to characterize macrophage subtypes present in the plaque. Six macrophage subclusters were observed (*Figure 3A* and *B*), and cluster-specific DEGs were examined (Supplementary data online, *Table S2*).

Based on these DEGs and known macrophage markers (Supplementary data online, *Figure S2A*-*C*), the subclusters were annotated as *TREM1^high^PLIN2^high^* (MO1), *TREM2^high^* (MO5), two clusters with a resident-like signature: *C1Q^high^, LYVE1^high^* tissue resident (MO4; *C1QA^high^, C1QB^high^, CD206^high^, CD209^high^, and LYVE1^high^*) and activated *C1Q ^+^ FCGR1A^high^* tissue resident (MO2; *HIF1A^high^, FCGR1A^high^, and C1Q*^+^) and finally two synthetic macrophage populations (MO3; *ACTA2^+^, MYH11^+^, MYH10^+^, CCN1^+^, TAGLN^+^, C1Q^+^, and CD74^+^*), strained synthetic macrophages (MO0; *ACTA2^+^, MYH11^+^, MYH10^+^, CCN1^+^, TAGLN^+^* and apoptosis genes *MALAT1* and *MTRNR2L12;*  Supplementary data online, *Figure S2D*).

Pathway-enrichment analysis based on DEGs was performed to determine biological processes associated with each subcluster (*Figure 3C*). The phagosome pathway was enriched in macrophage subclusters MO1, MO2, and MO5, while cholesterol metabolism pathways were enriched in the lipid associated macrophages (LAMs); subclusters MO1 and MO5. The antigen processing and presentation pathway was enriched in subclusters MO2 and MO4, highlighting the functional diversity of macrophage subtypes.

PAGA analysis revealed strong connectivity between LAMs; MO1 and MO5, MO2, and MO4 as well as between synthetic; MO0 and MO3 (*Figure 3D*). By RNA velocity, we also observed the presence of the inferred trajectories between: (1) MO5 to MO1, (2) MO3 to MO2, MO4, or MO0, (3) MO2 to MO4, and (4) from clusters MO2 and MO0 to MO1 (*Figure 3E*). The RNA velocity directions between the MO subclusters were observed within plaque microenvironments (*Figure 3F*).

### Characterisation and location of B-cell and endothelial cell subsets

To investigate cellular heterogeneity in the B-cell and EC clusters, clustering analyses were performed on spots from clusters 5 and 6, respectively (*Figure 1B*). Three clusters were found within the B-cell-dominant spatial cluster (Supplementary data online, *Figure S3*), and two clusters were found within the EC-dominant spot cluster (Supplementary data online, *Figure S4*). Highly expressed genes in each cluster and enriched pathways in each cluster were examined (Supplementary data online, *Tables S3*–4, Supplementary data online, *Figures S3* and *S4*). Of the three B-cell clusters, one was identified as plasma B-cells (B-cell2; *PRDM1^high^, XBP1^high^, MZB1^high^, IGKC^high^*, *IGHA1^high,^ IGLC1^high^*, and *IGLC2*^high^, Supplementary data online, *Figure S3C* and *D*), one was intermediate plasma cells (B-cells0; *IGKC^high^, IGHA1^high^, IGHG3^high^, IGHG4^high^, IGLC7^high^* and *IGLC3^high^*, Supplementary data online, *Figure S3C* and *D*) and one cluster with likely mixed cell types (B-cells1), with relatively low expressions of *IGKC*, *IGHA1* and *IGLC*2 compared to B-cell clusters 0 and 2 (Supplementary data online, *Figure S3E*). Cluster enriched pathways, connectivity and cell dynamic velocity was also examined (Supplementary data online, *Figure S3F*–*I*).

Two EC clusters were identified: one expressing canonical endothelial markers (EC1; *PECAM1^high^, VWF^high^* and *CDH5^high^*), and the other suggestive of an endothelial-mesenchymal transitional phenotype (EC0; *CDH11^high^, TAGLN^high^, FN1^high^* and *COL1A2^high^*; Supplementary data online, *Figure S4*).

### Intercellular communications within human plaque microenvironments

**Figure 4:**
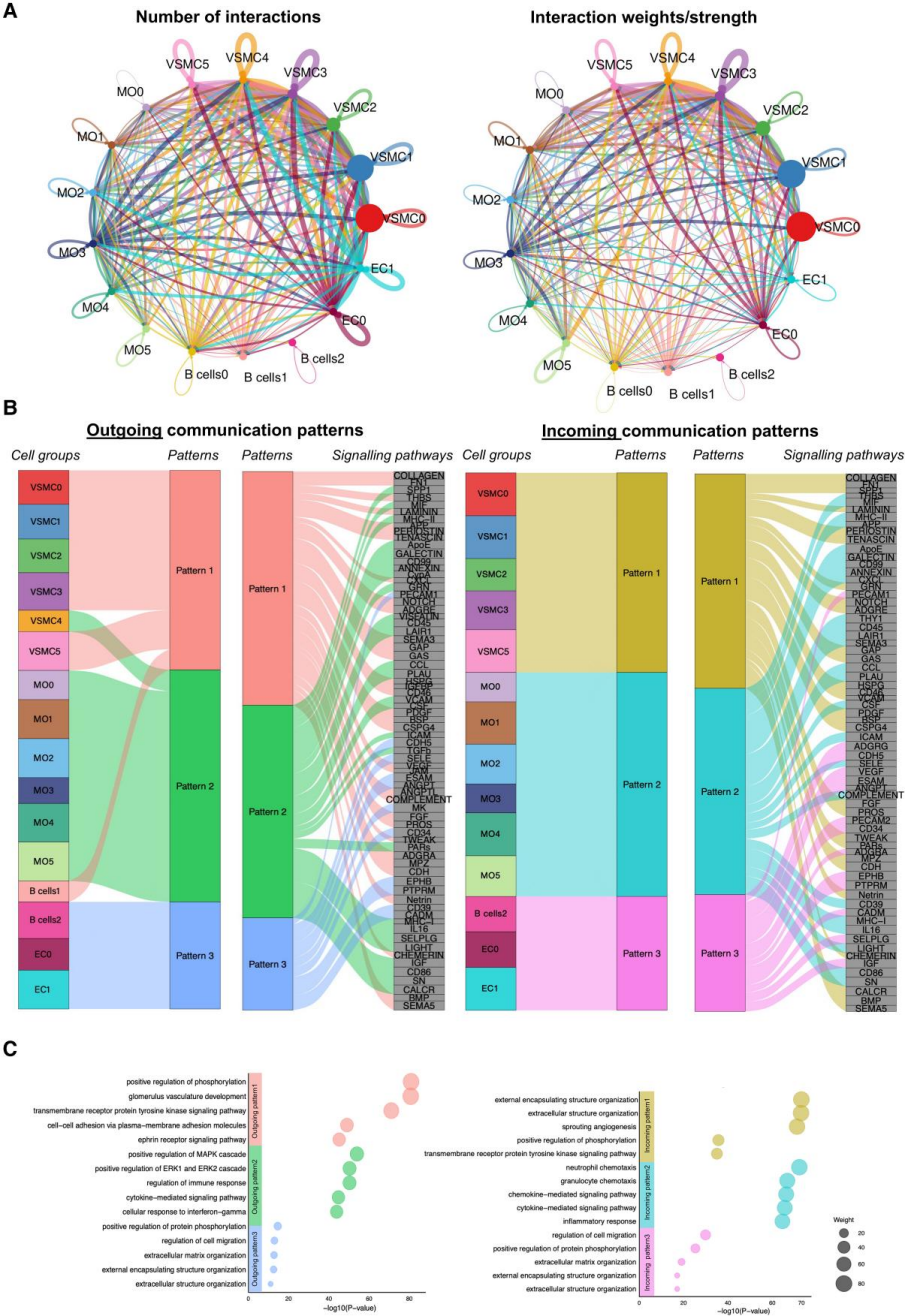
Spatially assessed cell–cell communication patterns in human atherosclerotic plaques. (*A*) Cell communication between the 17 identified cell subclusters, with line thickness representing the number of ligand–receptor interactions (left) and the total interaction strength between each pair of subclusters (right). (*B*) The inferred outgoing (left) and incoming (right) communication patterns of secreting and target cells reveal groups of sender (left) and receiver (right) cells that coordinate within specific signalling pathways to facilitate communication. (*C*) Enriched Gene Ontology (GO) terms for all ligand–receptor pairs involved in signalling pathways for each communication pattern. EC, endothelial cell; MO, macrophage; VSMC, vascular smooth muscle cell.

To understand cell–cell communications spatially, intercellular communication analyses were performed between the 17 identified spatial subclusters. CellChat identified 11 014 significant ligand–receptor pairs among these subclusters (*Figure 4A*). These interactions revealed three distinct patterns, with subclusters engaging in similar signalling pathways through both outgoing (*Figure 4B* left) and incoming (*Figure 4B* right) signals. VSMC outgoing signalling was predominantly characterized by pattern 1, which included COLLAGEN, FN1, THBS, and LAMININ pathways. Notably, COLLAGEN, FN1, and LAMININ pathways were also present in VSMC incoming signalling pattern 1. Macrophages outgoing signalling displayed the distinct pattern 2, including signalling pathways such as SPP1, MIF, ApoE, and GALECTIN. MIF, ApoE, and GALECTIN pathways were also observed in the incoming macrophage signalling. Interestingly, SPP1 signalling might play a paracrine-acting role between macrophages and VSMCs. Pattern 3 encompassed signalling pathways among EC and B cells, including CDH5, PECAM1, CD34, PTPRM, and EPHB pathways. The signalling pathways in each pattern (*Figure 4B*) show large similarity to the biological processes involved according to the Gene Ontology (GO; *Figure 4C*).

**Figure 5:**
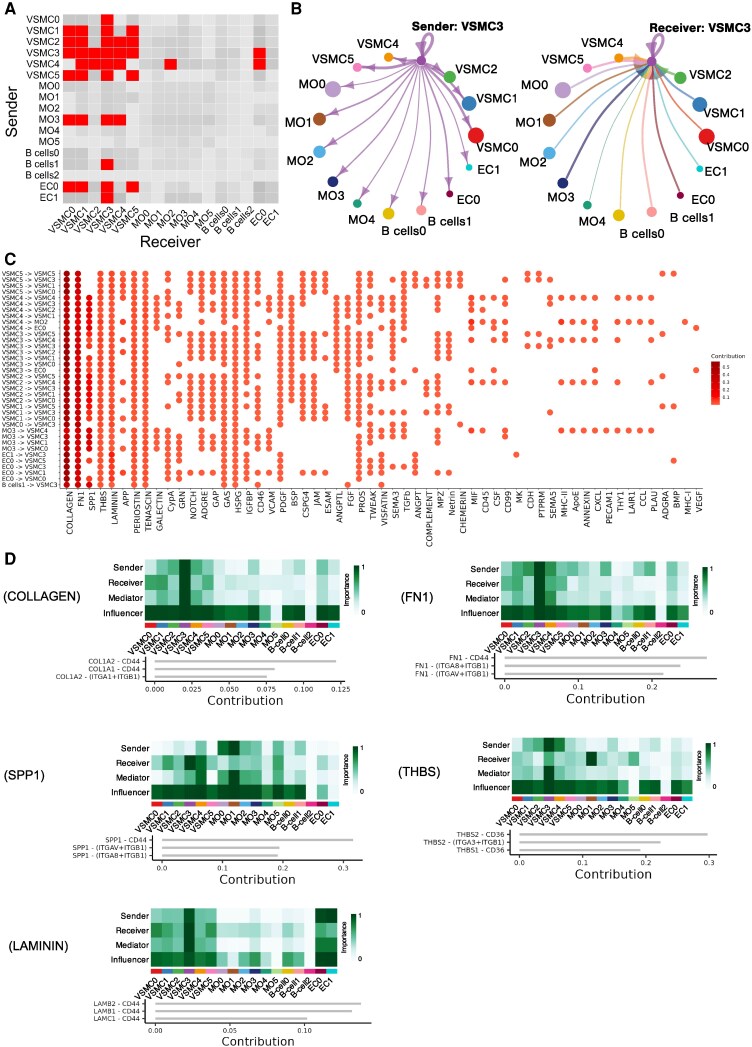
Fibroblast-like vascular smooth muscle cell is a key cell phenotype contributing to the major intercellular communication pathways. (*A*) Top 20% of intercellular communications among the 17 identified subclusters. Red blocks indicate the top 20% of cell-type interactions, ranked by both the number and overall strength of predicted ligand-receptor interactions. (*B*) Circle plots highlighting intercellular communications with fibroblast-like vascular smooth muscle cell (VSMC3), as senders (left) and receivers (right). Line weights indicate the total interaction strength between the sender and target cells. (*C*) Relative contribution of signalling pathways to the top 20% of intercellular communications between the 17 identified subclusters. (*D*) Heatmap showing signalling roles (senders, receivers, mediators, and influencers) of cell subclusters within COLLAGEN, FN1, SPP1, THBS and LAMININ signalling pathways, based on network centrality measures (out-degree, in-degree, flow betweenness, and information centrality). Top three ligand-receptor pairs driving the communication network within a signalling pathway are shown, based on their communication probability ratio in the inferred network. EC, endothelial cells; MO, macrophage; VSMC, vascular smooth muscle cell.

The top 20% of communicating pairs in terms of both interaction strength and number of interactions were highlighted (*Figure 5A*). VSMCs are highly ‘communicative’, showing strong intracluster communication, both as senders (to VSMC1-5) and receivers (from VSMC0-5), particularly VSMC3 (*Figure 5B*). VSMC4 demonstrated strong signal sending communication to macrophages (MO2), whereas VSMC3 and 4 showed strong sending communication to EC0. Both MO3 and EC0 were identified as senders to VSMCs (*Figure 5A*). Among those top 20% communicating subcluster pairs, 3500 significant ligand–receptor interactions were identified which were further categorized into 58 signalling pathways, with COLLAGEN, FN1, and SPP1 pathways emerging as the most prominent contributors to overall communications, followed by THBS and LAMININ (*Figure 5C*).

Focusing on the shortlisted pathways, the network analysis of COLLAGEN and FN1 signalling highlighted the critical role VSMC3 within the communication networks, serving as the dominant sender, receiver, and mediator. Namely, the ligand–receptor pairs, COL1A2 ligand and CD44 receptor contributed most to the COLLAGEN signalling (*Figure 5D* and Supplementary data online, *Figure S5*), whereas FN1 ligand and CD44 receptor contributed most to the FN1 signalling (*Figure 5D* and Supplementary data online, *Figure S6*).

Within the SPP1 signalling, MO1 emerged as senders and mediators, while VSMC3 was the main receiver, followed by VSMC4 (*Figure 5D* and Supplementary data online, *Figure S7*). Paracrine-acting VSMCs to macrophages were observed in THBS signalling, where VSMC3 acted as sender and receiver. In contrast, MO1 acted as a receiver (*Figure 5D* and Supplementary data online, *Figure S8*). In the LAMININ signalling, both EC subclusters (EC0 and EC1) were senders, while VSMC3 acted as both receiver and mediator (*Figure 5D* and Supplementary data online, *Figure S9*).

### Validation of CD44-COL1 signalling in fibroblast-like VSMC

**Figure 6:**
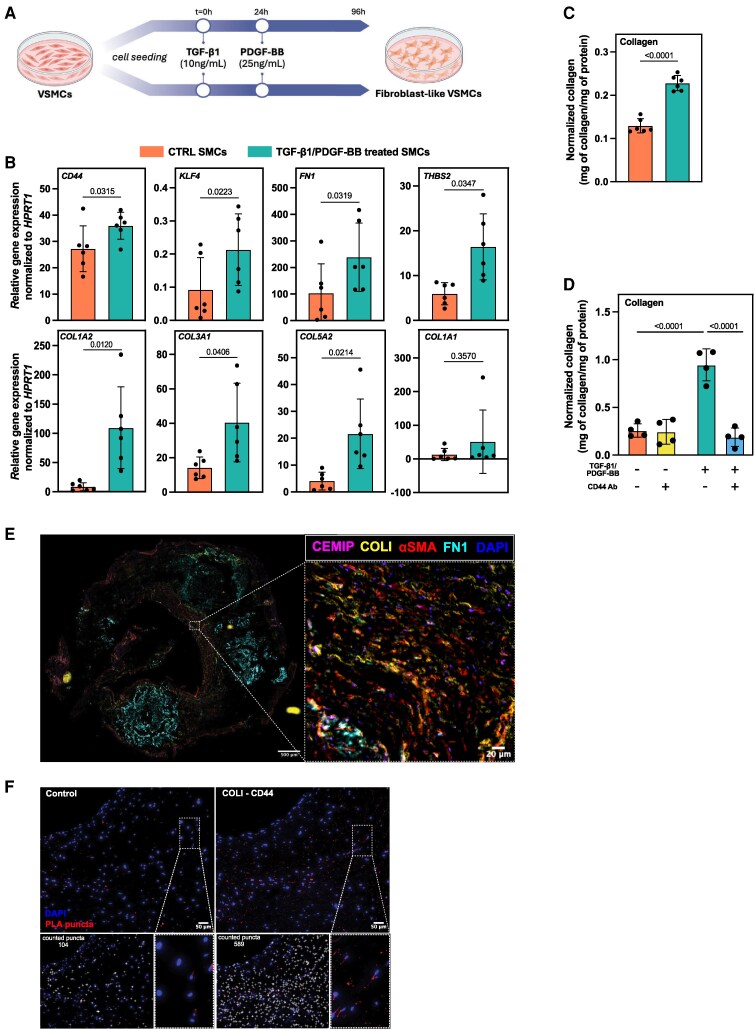
CD44-COL1 signalling modulates collagen production in differentiated fibroblast-like vascular smooth muscle cells *in vitro* and in human samples. (*A*) Fibroblast-like vascular smooth muscle cells (VSMCs) were differentiated using TGF-β1and PDGF-BB. (*B*) mRNA levels of key genes such as *CD44*, *KLF4*, *FN1*, *THBS2*, *COL1A2*, *COL3A1*, *COL5A2* and (*C*) collagen production were increased in fibroblast-like VSMC differentiated using TGF-β1and PDGF-BB. (*D*) Collagen production induced by TGF-β1 and PDGF-BB was halted when cells were co-cultured with CD44 blocking antibody (Ab). TGF-β1, transforming growth factor-β1; PDGF-BB, platelet-derived growth factor (*n* = 4). (*E*) Multiplex immunofluorescence staining was used to visualize the location and co-expression of key marker proteins of fibroblast-like VSMC3 in the cap of human atherosclerotic plaques. (*F*) Representative images show proximity ligation assay (PLA) signals (red puncta) and DAPI-stained nuclei (blue) in control (upper left) and CD44–collagen I (COL1) stained sections (upper right). Maximum intensity projections were generated from z-stacks spanning the full section thickness. Insets display magnified regions to highlight PLA puncta. The lower panels show the corresponding quantification, with individual detected PLA signals marked by white crosses. PLA puncta were selected based on morphological criteria—small area and high circularity—to distinguish specific interactions from background autofluorescence, which typically exhibits larger, irregular shapes. The number of detected puncta is indicated for each condition. DAPI (dark blue), CEMIP (magenta), COL I (yellow), aSMA (red), and FN1 (cyan). CEMIP, cell migration-inducing hyaluronidase 1; COL I, collagen type I; α-SMA, alpha-smooth muscle actin; FN1, fibronectin 1; PLA, proximity ligation assay. Created in BioRender. Gialeli, C. (2026) https://BioRender.com/hadvoej

TGF-β1 and PDGF-BB stimulation was used to induce a fibroblast-like VSMC phenotype, mimicking VSMC3, characterized by upregulation of *CD44, KLF4, FN1, THBS2, COL1A2, COL3A1,* and *COL5A2,* showing increased collagen production (*Figure 6A–C*). Blocking CD44 suppressed the collagen production (*Figure 6D*).

Additionally, *in silico* knock-out of *FN1, SPP1, CD44, CD36,* and *COL1A2* in VSMC3 demonstrated enrichment in elastic fibre formation (Supplementary data online, *Figure S10*).

Finally, we confirmed the co-expression of VSMC3 marker proteins and the interaction between CD44 and collagen I in the human fibrous cap using multiplex immunofluorescence and proximity ligation assay (*Figure 6E and F*).

### Intercellular communication in plaque regions

**Figure 7:**
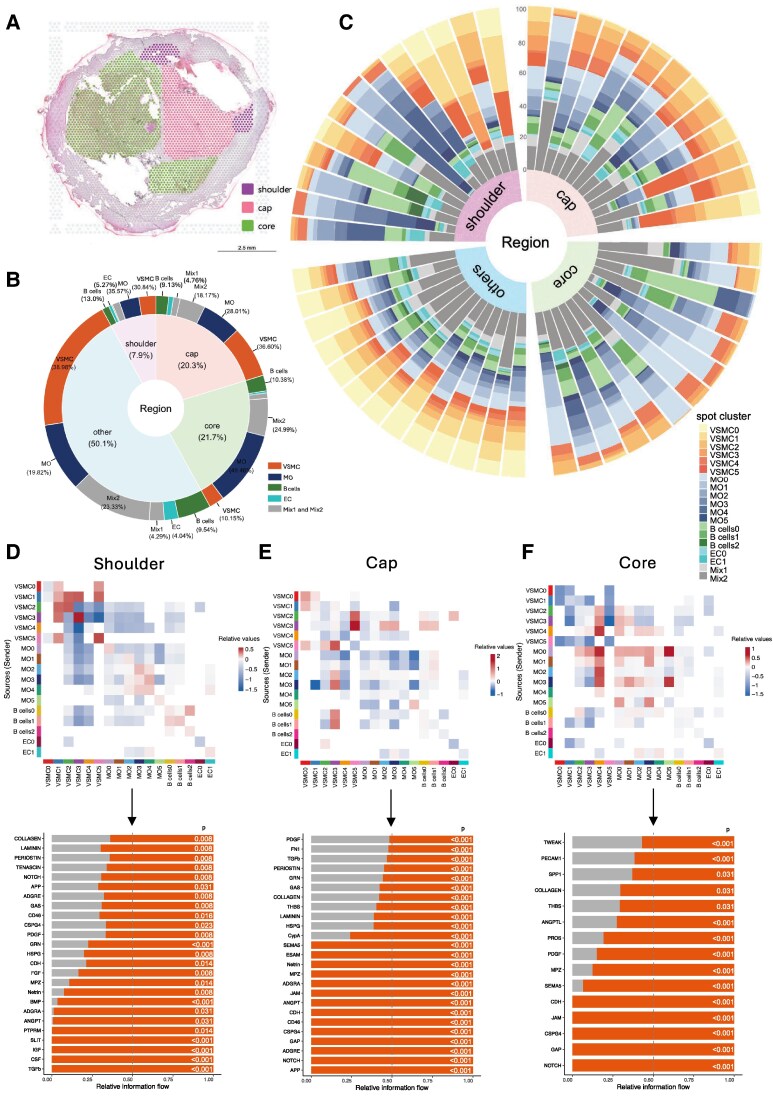
Regional variations in cell abundance and intercellular communication. (*A*) Schematic illustration of shoulder, cap and core regions in a human carotid plaque tissue section stained with haematoxylin and eosin. (*B*) Donut plot depicting the composition of plaque regions and the cell type distribution within each region. Cell types with percentage above 4% are shown. (*C*) Circle stacked bar plot showing spot subclusters comparing cap, shoulder, core and other regions (inner ring) for each plaque among individuals. (*D–F*) Heatmaps visualizing plaque regional differences in intercellular communication and signalling pathways that are increased or turned on at (*D*) shoulder, (*E*) cap, and (*F*) core regions. Senders are shown on the X-axes and the receivers on the Y-axes. Red blocks represent increased communication strength, while blue indicates decreased strength. Increased signalling pathways were identified by comparing interaction strengths within VSMCs between shoulder and non-shoulder (cap and core) regions (VSMC3 to VSMC1/3, VSMC1 to VSMC2/3/5, VSMC2 to VSMC1/2 and VSMC5 to VSMC5, cap and non-cap (shoulder and core) regions (VSMC2 to VSMC5, VSMC3 to VSMC5 and VSMC5 to VSMC3), and interaction strengths within macrophage-foam cells between core and non-core (cap and shoulder) regions (MO0 to VSMC4, MO3 to VSMC4, MO0 to MO5, MO3 to MO5, MO2 to VSMC4 and VSMC4 to VSMC4). Orange colour for cap, core, and shoulder regions; grey colour for non-cap, non-core, and non-shoulder regions. EC, endothelial cell; Mix1 and Mix2, mixed cells without a predominant cell type; MO, macrophage; VSMC, vascular smooth muscle cell

One of the main novelties of this study was the spatial mapping of the ligand–receptor communication between cells in key regions within the plaques (identified by H&E tissue staining). Plaque regions were categorized into shoulders (7.9%), core (21.7%), fibrous cap (20.3%) and other regions, beyond these three main regions (50.1%; *Figure 7A* and *B*). Regional and spot cluster compositions within each region were analysed (*Figure 7B* and *C* and Supplementary data online, *Figure S11*). The median Bray–Curtis dissimilarity was 0.15, indicating low differences in cell composition among the 13 plaques. Overall, the most abundant cell spot clusters in carotid plaque sections were VSMC (31.6%) and macrophage (29.2%) clusters. In the shoulder regions, 30.8% of the spots were VSMC clusters, while 35.6% were macrophage clusters. In the core regions, most spots (49.5%) were macrophage clusters. In the fibrous cap regions, 36.6% of the spots belonged to VSMC clusters, followed by macrophage clusters (28.0%, *Figure 7A* and *B*).

We then aimed to detect differences in interaction strengths within the plaque regions across the different cell subclusters. Changes in signalling among VSMC clusters, in particular, fibroblast-like VSMC (VSMC3) and contractile VSMC (VSMC1 and VSMC5), within the shoulder (VSMC3 to VSMC1/3, VSMC1 to VSMC2/3/5, VSMC2 to VSMC1/2 and VSMC5 to VSMC5) and the cap (VSMC2 to VSMC5, VSMC3 to VSMC5 and VSMC5 to VSMC3) regions (*Figure 7D* and *E*) were identified. When examining the pathways for the increased signalling in specific plaque regions, we identified common extracellular matrix-associated pathways (e.g. LAMININ, PERIOSTIN, and COLLAGEN) and growth factors-associated signalling (e.g. PDGF and TGFβ) in the shoulder and the cap regions (*Figure 7D* and *E*). Notably, the shoulder region showed marked enrichment in growth factor-related pathways including TGFβ, CSF and IGF (*Figure 7D*).

In the core region, an increased communication was predominantly observed within the macrophage subclusters, in particular, MO0, MO3, and the macrophage-foam cell-like VSMC4 (MO0 to VSMC4, MO3 to VSMC4, MO0 to MO5, MO3 to MO5, MO2 to VSMC4, and VSMC4 to VSMC4; *Figure 7F*). Pathways accounted for the increased signalling among these cell phenotypes in core regions were related to angiogenesis and endothelial activation, such as ESAM and NETRIN, and inflammation (e.g. NOTCH, GAP, JAM, CDH), many of which were also observed in the increased signalling within the cap.

### Clinical evidence for the association between the fibroblast-like VSMC3, stable plaque phenotype and future CV events

**Figure 8:**
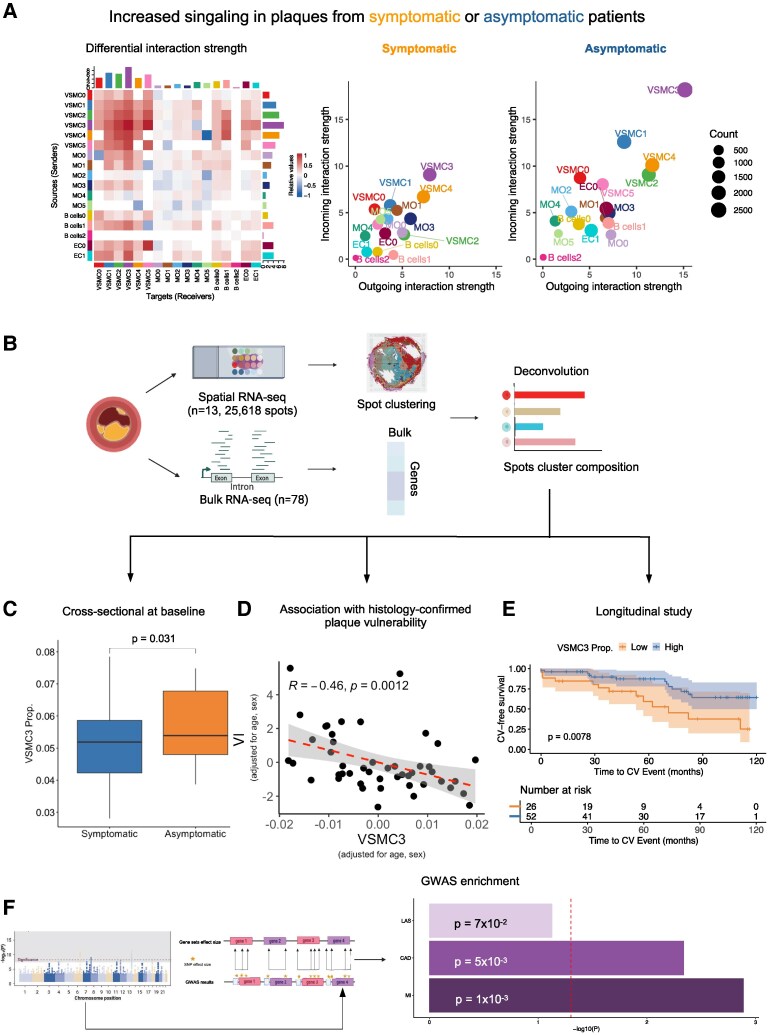
Fibroblast-like vascular smooth muscle cells (VSMC3) are associated with a stable plaque phenotype and a lower risk of clinical events. (*A*) Strength of cellular interactions in symptomatic and asymptomatic plaques. The coloured bar plot on the top of the heat map represents the summarized strength of all the incoming signalling per spot cluster. The coloured bar plot on the right represents the summarized strength of all outgoing signalling per spot cluster. In the colour bar, red (or blue) represents increased (or decreased) signalling comparing asymptomatic and symptomatic plaques. *n* = 13. (*B*). Schematic illustration of spot cluster deconvolution applied on human carotid plaque bulk RNA-Seq, using all the identified spatial RNA-Seq spot clusters as reference data. (*C*) Based on the deconvolution analysis, the proportion of fibroblast-like VSMC (VSMC3) was shown to be significantly higher in carotid plaques from asymptomatic individuals (*n* = 27 plaques) compared to individuals who suffered from cerebrovascular symptoms at baseline (*n* = 51). Student’s *t*-test was used. (*D*) The proportion of VSMC3 was inversely correlated with a calculated histological plaque vulnerability index (VI). Spearman´s correlation analysis adjusted by age and sex was used (*n* = 47). (*E*) Kaplan–Meier curve visualizing that patients with plaques with a greater proportion of VSMC3 (second to third tertiles compared to the first tertile) were at lower risk to suffer from postoperative cardiovascular events during the follow-up (*n* = 78). Log-rank test was used. (*F*). Schematic illustration (left) of the gene-set analysis of GWAS data. Bar chart (right) showing the *P*-values from GWAS enrichment analyses for VSMC3 marker genes in LAS, CAD, and MI. Vertical line indicates a *P*-value of .05. CAD, coronary artery disease; EC, endothelial cell; LAS, large artery stroke; MI, myocardial infarction; Mix1 and Mix2, mixed cells without a predominant cell type; MO, macrophage; Prop., proportion; VI, vulnerability index; VSMC, vascular smooth muscle cell. Created in BioRender. Sun, J. (2025) https://BioRender.com/ex05rdm

When analysing novel intercellular communications within the plaque microenvironment, increased signalling was observed within VSMC clusters in plaques from asymptomatic patients (*Figure 8A*). Notably, the fibroblast-like VSMC (VSMC3) contributed significantly to the increased signalling in asymptomatic plaques (*Figure 8A*), again highlighting their pivotal role in plaque cell communication.

The spot cluster deconvolution to bulk RNA-seq data of human carotid plaques (*n* = 78) was applied to estimate spot cluster composition for each plaque (*Figure 8B*). Of great interest, the proportion of VSMC3 was significantly greater in plaques from asymptomatic (*n* = 27) compared to symptomatic patients (*n* = 51; *P* = .03, *Figure 8C*). In line with that, the proportion of VSMC3 inversely correlated with the previously used histological plaque vulnerability index^14,25^ (r = −0.46, *P* = .0012; *Figure 8D*), indicating a strong negative association with a vulnerable plaque phenotype.

Furthermore, we explored if VSMC3 could predict future CV events. During the follow-up period [up to 10 years, interquartile range (IQR) 2.6–7.3 years], individuals with a greater proportion of VSMC3 (second to third tertiles compared to first tertile) at baseline had a lower risk of suffering from future CV events (*P* = .0078; *Figure 8E*). Similar analyses for all VSMC were shown in Supplementary data online, *Figure S12*.

Finally, using large-scale GWAS data to support the relevance of this novel VSMC phenotype, the gene-set analysis by MAGMA showed that VSMC3 marker genes were significantly enriched in CAD and MI, whereas a trend was observed for LAS (*Figure 8F*).

Together, these findings suggest that VSMC3 contributes to a more stable plaque phenotype.

### Human fibroblast-like VSMC show similarity to the fibrous cap–associated VSMC state in the *Apoe*^−/−^ mouse model of atherosclerosis

**Figure 9:**
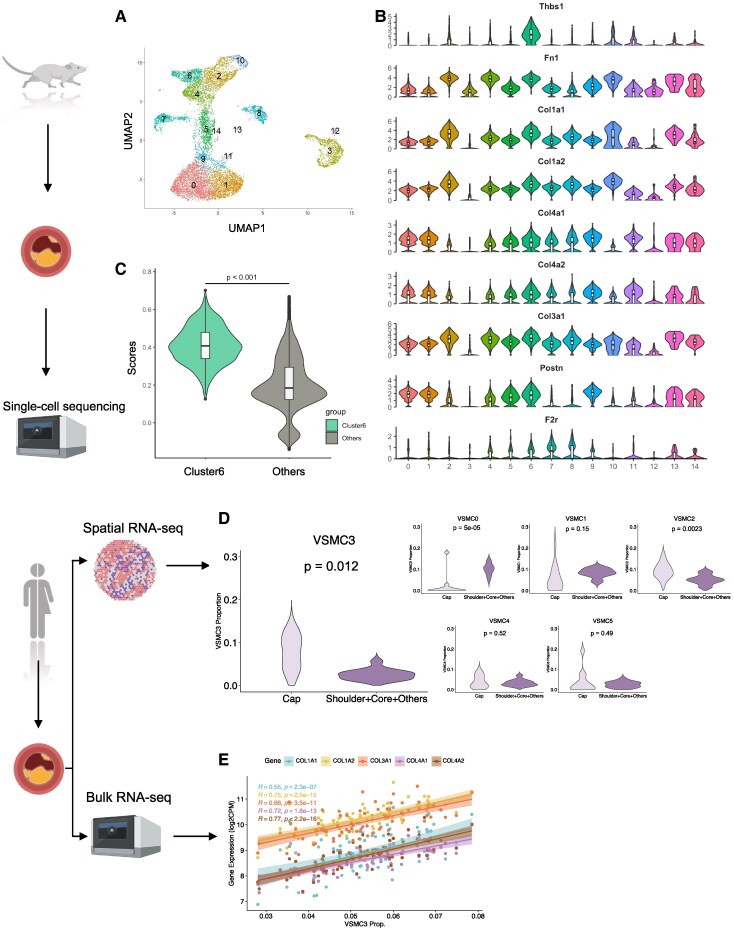
Fibroblast-like vascular smooth muscle cells (VSMC3) have a high similarity with fibrous cap associated VSMC in *Apoe^−/−^* mice. (*A–C*) scRNA-Seq analysis of lineage-labelled VSMCs from control (GSE274572) and atherosclerotic *Apoe^−/−^* mice on western diet (GSE155513). (*A*) Uniform manifold approximation and projection visualizing cell clusters identified by Taylor *et al.*^21^ (*B*) Violin plots showing expression of selected VSMC3 marker genes across all the identified mouse cell clusters. (*C*) Violin plot showing UCell scores for the VSMC3-marker gene signature in cells proposed to represent fibrous cap–associated VSMCs (mouse cell cluster 6) compared to all other VSMC phenotypes. Student’s *t*-test was used. (*D*) Spatial transcriptomics analysis of human carotid plaques showing an enrichment of fibroblast-like VSMC (VSMC3) and macrophage-like VSMC (VSMC2) spots in the cap regions compared to the non-cap regions (shoulder, core and other regions). Student’s *t*-test was used. *n* = 13. (*E*). Scatter plot showing positive correlations between collagen genes mRNA levels and VSMC3 proportion in human carotid plaques. *n* = 78. Pearson’s correlation coefficients are shown. Created in BioRender. Sun, J. (2025) https://BioRender.com/ex05rdm

Human fibroblast-like VSMCs were compared to lineage-labelled VSMCs from atherosclerotic plaques of *Apoe^−/−^* mice (8, 16, and 22 weeks old) fed with high fat diet (Supplementary data online, *Figure S13*).^26^ Using a clustering defined by Taylor *et al.*^21^, a cluster of fibrous cap-associated VSMC (mouse cell cluster 6) was identified, as well as previously identified contractile VSMCs (cVSMC, mouse clusters 0, 1, 7, 9, 11), intermediate modulated VSMCs (imVSMC, mouse clusters 4), chondromyocytes (CMC, mouse clusters 2 and 10), fibroblast-like (mouse cluster 8), VSMC-origin macrophage-like cells (mouse clusters 3 and 12; *Figure 9*).

High gene expression of the human VSMC3 marker genes (*THBS1*, *FN1*, *COL1A1*, *COL1A2*, *COL4A1*, *COL3A1*, *POSTN*, and *F2R*) was identified in the fibrous cap–associated VSMC cluster (mouse cluster 6, *Figure 9B*). A signature scoring analysis demonstrated that the gene expression profile of the human VSMC3 phenotype more closely resembled that of fibrous cap–associated VSMCs compared to other VSMCs in mice (*P* < .001, *Figure 9C*). This suggested that VSMC3 were fibrous cap-associated. Accordingly, the human spatial transcriptomics confirmed that the proportion of VSMC3 was significantly greater in the human cap regions compared to the other plaque regions (*P* = .012; *Figure 9D*, Supplementary data online, *Figure S14*). Furthermore, strong correlations between fibrous cap–associated collagen genes and the proportion of VSMC3 were observed in the human plaque bulk RNAseq deconvolution (*Figure 9E*).

### Validation of fibroblast-like VSMCs (VSMC3) in coronary atherosclerosis

The presence of fibroblast-like VSMC in atherosclerotic plaques from different arterial beds was examined using large public single cell datasets. From that, the VSMC phenotype most similar to VSMC3 (found in both human carotid and coronary plaques) was in an *MMP2*^high^, *LUM*^high^ and *F2R*^high^ cell cluster (Supplementary data online, *Figures S15*). Additionally, the role of fibroblast-like VSMCs in regulating COLLAGEN, FN1, and SPP1 signalling was confirmed in human coronary plaques (Supplementary data online, *Figure S15E*), even though it is impossible to generalize the data obtained in carotid plaques to what occurs in coronaries.

### Candidates therapies to induce fibroblast-like VSMCs (VSMC3) marker genes

**Figure 10:**
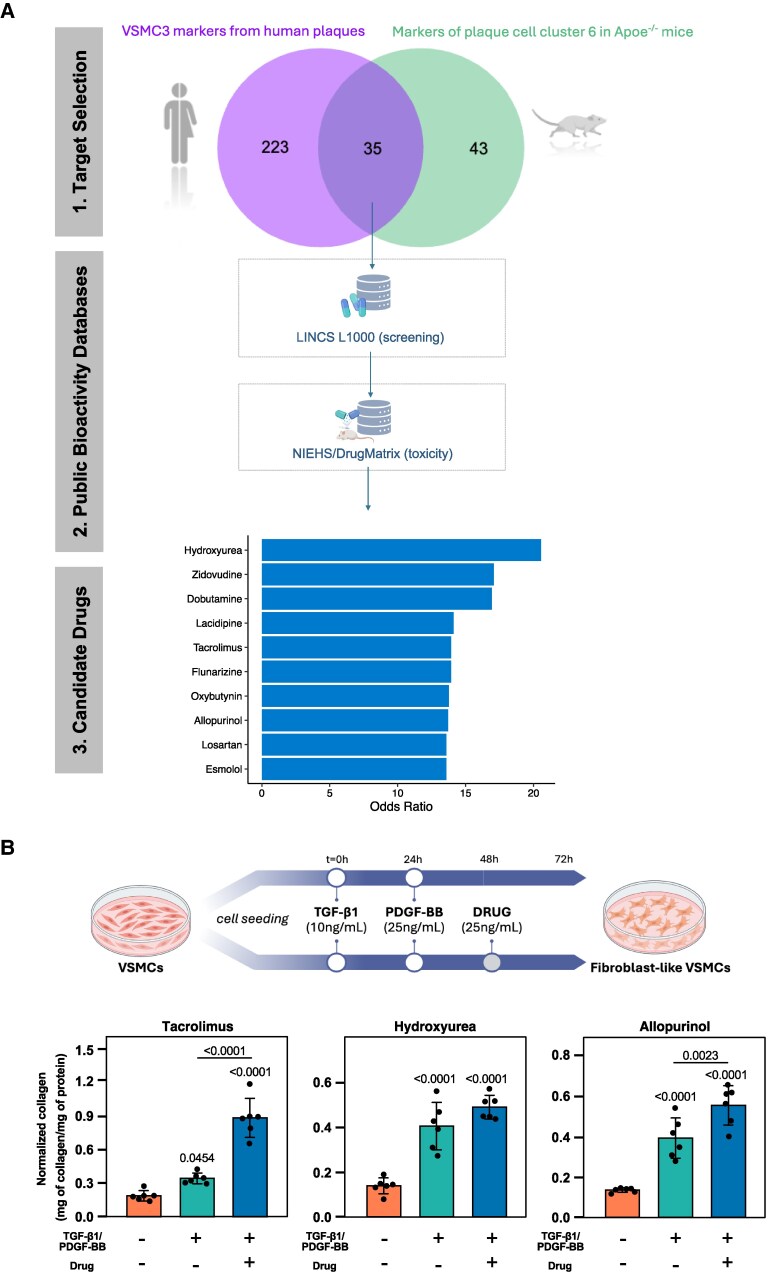
Potential therapies to stimulate vascular smooth muscle cell 3 (VSMC3) marker genes. (*A*) A schematic illustration showing the workflow to identify compounds that target VSMC3 marker genes in human cell lines. Step1, 35 overlapping genes highly expressed in both human fibroblast-like VSMCs (VSMC3) and the phenotypically similar fibrous cap-associated VSMCs from *Apoe^−/−^* mice were identified. Step2, Exploring public bioactivity databases for drugs. First, LINCS L1000 chemical perturbation data was used to screen compounds that can upregulate gene expression of these 35 marker genes in human cell lines. Next, the selected compounds that have potential beneficial transcriptional effects in rat were examined using the DrugMatrix toxicogenomic database. Analyses were conducted through Enrichr and odds ratio per compound is reported. Step 3, Odds ratios were used to rank the 38 identified drug candidates, highlighting the top 10 potential therapies. (*B*) Collagen production from fibroblast-like VSMCs was increased upon treatment with tacrolimus, hydroxyurea and allopurinol. PDGF-BB, platelet-derived growth factor; TGF-β1, transforming growth factor-β1 (*n* = 6). Created in BioRender. Sun, J. (2025) https://BioRender.com/ex05rdm

As our findings suggest a potential protective role of VSMC3, we explored whether existing drugs could stimulate VSMC3 marker genes. Thirty-five overlapping marker genes, highly expressed in human and mouse fibroblast-like VSMC, were used for the analysis (*Figure 10A*). With LINCS L1000 chemical perturbation data, 1960 compounds were suggested to affect the expression of these 35 marker genes in human cell lines (BH-adjusted *P* < .05). Thirty-eight compounds had beneficial transcriptional effects in high-dose exposures in rat tissues (Supplementary data online, *Table S5*). Among the top 10 compounds ranked by the estimated effect, several clinically used therapies were identified: hydroxyurea, tacrolimus, losartan, allopurinol, zidovudine, dobutamine, and lacidipine (*Figure 10A*). An *in-silico* drug score analysis was also performed, suggesting that losartan, allopurinol, and hydroxyurea treatments were associated with increased VSMC3 proportions in human carotid plaques (Supplementary data online, *Figure S16*). A positive trend for the use of tacrolimus was observed.

Finally, to test if these therapies hold potential for modulating VSMC3 and contribute to fibrous cap formation fibroblast-like VSMC were differentiated *in vitro* and stimulated with tacrolimus, hydroxyurea, and allopurinol. All three therapies induced collagen synthesis (*Figure 10B*). However, further investigation on the drugs is warranted even if beyond the scope of this study.

## Discussion

Recent single-cell sequencing studies have greatly advanced our understanding of cell diversity in atherosclerosis, revealing distinct cell phenotypes and their functions beyond traditional surface markers.^10,27–30^ Advances in spatial RNA sequencing have further mapped where these cells are distributed within the plaques.^6–8^ However, the intercellular communications, their precise spatial location, and trajectories of these cells within the plaque’s micro-architecture and how this affects the clinical phenotype of the plaque and the patients’ future CV risk remain largely unexplored. This knowledge is crucial to uncover disease-specific mechanisms and potential treatment targets *in situ*.

This study integrated multimodal RNA sequencing (spatial, single cell, and bulk), GWAS analyses, human histology, follow-up studies, a murine model and *in vitro* functional assays to provide novel insights into the communication between disease-defining cellular populations by mapping their interactions to their spatial locations and characterizing spatial dynamics of cell state changes (RNA velocity) within advanced human carotid atherosclerotic plaques for the first time. The fibroblast-like VSMC stood out as pivotal in intracellular communication within the human plaque microenvironment, strongly linked to the stable plaque phenotype, fibrous cap formation (in mice, human and *in vitro*), and predicted lower risk of CV events, highlighting their key role in human atherosclerosis. Repurposed drugs against the key marker genes of these cells increased collagen production *in vitro*, opening for potential future therapeutical applications.

### VSMC are the most abundant cells in the plaque

Among the eight distinct major cell clusters identified from spatial transcriptomic of the human plaques, the spots majority was VSMCs, followed by macrophages. This finding aligns with recent studies using single-cell or bulk RNA-seq deconvolution,^29,31,32^ suggesting that VSMCs are commonly overseen in biological processes in atherosclerosis.

When subclustering the VSMC, the six transcriptional states identified were separated into two major trajectories based on their connectivity: (1) a contractile community (VSMC0/VMSC1/VSMC5), (2) a modified community, including two macrophages-like VSMC clusters (VSMC2/VSMC4) and a fibroblast-like VSMC cluster (VSMC3) with synthetic behaviour. The spatial gene expression and RNA velocity analyses suggested that contractile VSMCs near the medial layer (VSMC0) gradually shift towards a more synthetic phenotype (VSMC1), supporting a continuum of VSMC differentiation.^33,34^ This was consistent with a large-scale scRNA-seq study suggesting that *MYH11^+^* contractile VSMCs are a likely starting point for VSMC differentiation.^29^ Our current findings provide the first spatial- and RNA velocity-based evidence to support that.

The contractile VSMC (VSMC5) could transition into a fibroblast-like phenotype (VSMC3), and possibly reverted back into a contractile state (VSMC1), indicating reversible differentiation. These phenotypes showed distinct spatial patterns: contractile cells near the media (VSMC0) and cap (VSMC5), whereas contractile synthetic (VSMC1) and fibroblast-like (VSMC3) cells are nearby, mostly in caps and shoulders.

### Synthetic macrophages accumulate in the core with pro-inflammatory foam cells

Six phenotypes of macrophages were identified, including two clusters of tissue-resident (MO2 and MO4), *TREM2^high^* (MO5) and *PLIN2^high^TREM1^high^* (MO1) macrophages. Beside these well-characterized clusters, two clusters with synthetic transcriptional signatures resembling VSMC-like macrophage phenotypes (MO0 and MO3) were also identified. MO0 appeared to be more activated and under stress, expressing higher levels of genes involved in apoptosis (*MALAT1* and *MTRNR2L12*, Supplementary data online, *Figure S2D*) compared to MO3 macrophages. The spatial distribution supported this as MO0 were predominantly found in the core regions, a hostile microenvironment known for its hypoxic and pro-apoptotic conditions.^35,36^ RNA velocity suggested a transition from MO3 towards MO0, potentially marking MO0 as a terminal state. This aligns with the idea that the MO0 reflects a phenotype subjected to harmful core conditions, leading to a pro-apoptotic transcriptional signature.

The RNA velocity analysis indicated a differentiation trajectory from a *TREM2^high^* phenotype towards the highly pro-inflammatory *PLIN2^high^TREM1^high^* transcriptional state, consistent with previous results^10^ and also supported by the PAGA analysis revealing strong connectivity between *TREM2^high^* (MO5) and *TREM1^high^PLIN2^high^* (MO1). Regarding the spatial location, these clusters were near the shoulders and in the core regions, respectively, in agreement with the previous notion of *TREM2^high^* and *PLIN2^high^TREM1^high^* myeloid cells playing a role in necrotic core formation.

### Intercellular communication and signalling patterns characterize cell-specific regulation of biological processes

One of our main aims was to map extensively the communication between cell phenotypes involved in larger interaction numbers and strengths (*Figs 4A* and *5*): crucial to guide future studies aiming for cellular therapeutic modulation. Building on the spatial plaque location of the cell phenotypes, key intercellular signalling pathways were unveiled revealing three main interaction patterns: (1) VSMC-driven, (2) macrophage and foam cell-like VSMC-driven, and (3) B-cell and EC-driven (*Figure 4B*). VSMCs mainly regulated extracellular matrix pathways (e.g. COLLAGEN, FN1, THBS, and LAMININ),^37–41^ macrophages were enriched in inflammatory signalling (e.g. SPP1, MIF, ApoE, and GALECTIN),^42–46^ and B-cells/ECs were linked to angiogenesis and adhesion (e.g. CDH5, PECAM1, and EPHB).^47–51^ Besides unearthing key signalling pathways, the strong autocrine signalling by VSMCs stood out.

### Specific regional differences in intercellular communication

A further novelty of this study was the regional mapping of the intercellular signalling in the advanced plaques, bringing a new ‘geographic’ or spatial perspective. In the core, the heightened signalling from macrophage subclusters MO0, MO3, and MO5, as well as in macrophage-foam cell-like VSMC was evident. Accordingly, a distinct pattern of enriched signalling in the inflammatory process was seen in this region (NOTCH, GAP, JAM, and CDH), affecting key functions such as immune cell differentiation, migration, and activation. A large overlap of these pathways was seen in the cap (which is consistent with its frequent inflammation in advanced lesions) mainly observed within contractile, synthetic and macrophage-like VSMC communications. In caps, some of the enriched pathways were very similar to those found predominantly in shoulders, namely enriched extracellular matrix-related signalling pathways (COLLAGEN, LAMININ, and PERIOSTIN), witnessing for the permanent struggle of the cap to remain stable and fibrotic. Finally, the shoulders, known by intense activity, with pronounced communication among contractile, synthetic and macrophage-like VSMC (VSMC1/2/3/5), demonstrated distinctly enriched signalling of growth factor-associated pathways (TGFb, IGF, and CSF). Collectively, these pathways likely reflect fibrous repair signalling in the cap and shoulder, critical for plaque stability.

### Fibroblast-like VSMCs play a key role in human plaque intercellular signalling, contribute to plaque stability, and predict fewer future clinical events

Of utmost novelty, when analysing the top 20% of all cell signalling, the fibroblast-like VSMC (VSMC3) emerged as a key player—particularly via COLLAGEN, FN1, LAMININ, and THBS—functioning as both strong senders and receivers of signals, communicating with a wide range of cells, including all VSMC subtypes, ECs and VSMC-like macrophages (MO3). The fibroblast-like VSMC3 stood out as major drivers of critical biologic processes within the plaque microenvironment.

We also aimed to explore if specific intercellular communication and signalling patterns could separate plaques from patients with a recent plaque rupture and symptoms vs asymptomatics. Strikingly, VSMC3-dependent signalling was enhanced in asymptomatic plaques suggesting a potential protective role of intercellular communication through VSMC3. To validate this, deconvolution of a larger set of human carotid plaques was performed. In line with the intercellular signalling, the proportion of VSMC3 was significantly higher in asymptomatic compared to symptomatic plaques. The proportion of VSMC3 also correlated inversely with the plaque vulnerability index (an independent predictor of future CV events^14^). Finally, increased VSMC3 proportion predicted reduced risk of future CV events longitudinally.

Providing further insight on the VSMC3 phenotype role, we demonstrated their similarity comparing with plaque VSMC phenotypes from an *Apoe^−/−^* model, where these cells were predominantly identified in the fibrous cap.^21^ Supporting their role in cap formation also in humans, the VSMC3 were enriched in the human cap regions and strongly correlated with collagen genes, also predominantly expressed in the fibrous cap.^21,52,53^ As additional functional evidence, increased expression of matrix-related genes and elevated collagen production was demonstrated *in vitro* in induced fibroblast-like VSMC resembling VSMC3. This could be reversed using CD44 antibodies, blocking that ligand-receptor communication (visualized in human plaque cap with multiplex immunofluorescence and proximity ligation assays) and even supported by *in silico* knock-out models.

As a final level of evidence, in large population GWAS studies, VSMC3 genes were enriched in CAD and MI. The role of VSMC in plaque stability has long been known,^54,55^ but we here bring an incremental granularity with the exact phenotype that underlies stability, confirming them in mice, *in vitro* and in a longitudinal cohort. Clonal expansion of few VSMCs that generate the range of diverse plaque cell states was shown in mouse^56^ and suggested in humans.^57^ Such shared origin of the cells in different plaque regions opens now further the exciting potential of plaque stabilization by drugs inducing the VSMC3 state of clonal cells.

### Potential therapies targeting fibroblast-like (VSMC3) marker genes

To assess whether key VSMC3 genes could be targeted to promote fibrous cap formation and plaque stability, publicly available pharmacogenomic databases were used, showing that 38 existing therapies upregulated 35 key VSMC3 marker genes (common to human and mice). Several are already used in the CV field like losartan, dobutamine, and tacrolimus. Tacrolimus, frequent in stents, may promote collagen synthesis and fibrous cap formation,^58^ which aligned with our *in vitro* results. In contrast, drugs like losartan and hydroxyurea may reduce collagen synthesis^59^ even if affecting VSMC3 marker genes. These findings suggest that VSMC3 function could potentially be modulated even by existing therapies, though further studies are needed.

### Limitations

First, the spatial transcriptomic technology used does not offer single cell resolution (in contrast with other now available techniques^60–62^) as each spot may encompass 1 to 10 cells (depending on cell size). Although individual spot may contain multiple cell types, cell type prediction revealed predominant cell types in the spots corresponding to the six major cell clusters, with low heterogeneity in plaque spot cluster composition. Nevertheless, not all sources of biological heterogeneity can be excluded. Some cell types (e.g. T-cells) were not identified as major clusters. T-cells were predicted within the mixed clusters, not distinctly separated from macrophages and VSMCs, even though this has previously been reported by others.^8^ The used spatial sequencing technology performed on fresh-frozen plaque sections offered untargeted, deep sequencing of all cells present in the tissue section spots, enabling the assessment of RNA velocity (not possible using currently available single cell spatial transcriptomic approaches). This avoided biases associated with cell loss during isolation or region of interest preselection. It also provided advantages over single-cell RNA sequencing by reducing the influence of circulating cells and, most importantly, by preserving the spatial context of the cell phenotypes −essential when assessing communication among neighbouring cells.

Second, despite comprehensive mapping of intercellular communication across plaque regions, additional low-expression interactions may have been missed or filtered during quality control. Among >11 000 significant ligand–receptor pairs, only one (CD44-COL1A2) was functionally validated *in vitro* as proof-of-concept for its role in cap formation.

Third, while more complex animal models (e.g. deletion of the human-VSMC3 equivalent cell type) could be explored, such studies would be resource-intensive, of uncertain viability and might not accurately have reflected human disease due to interspecies differences.

Fourth, since only advanced carotid plaques were analysed (no control segments were included), results may not generalize to other arterial territories, healthy arteries or earlier disease stages. Plaques from symptomatic patients obtained after a cerebrovascular event (within 22 days) might have already ongoing repair processes affecting the results.

Finally, even though several lines of evidence suggest an important role of the fibroblast-like VSMCs in plaque stability, no causality could be shown.

## Conclusion

In conclusion, this study provides a comprehensive novel spatial characterisation of human carotid plaque cell phenotypes and, for the first time, maps intercellular ligand-receptor communication in distinct ‘geographic’ plaque microenvironments. By verifying increased collagen formation *in vitro*, cap formation mechanisms in a mouse model, integrating human histological, GWAS and longitudinal survival analysis, fibroblast-like VSMCs emerged as key ‘communicators’ orchestrating specific intercellular signalling pathways, one of which was functionally validated and pharmacologically modulated *in vitro* and *in silico* using repurposed drugs. These findings open new avenues for targeting ligand-receptor interactions mediated by specific cell phenotypes to modulate cell communication and stabilize human atherosclerotic plaques.

## Supplementary Material

ehaf1091_Supplementary_Data

## Declarations

### Data Availability

Individual data regarding living humans cannot be publicly available due to the sensitive nature of the data regulated by European privacy laws (GDPR). It might be accessed for the sole purpose of replicating the procedures and results presented in the article and providing that the data transfer is in agreement with European Union legislation on the general data protection regulation and decisions by the ethical review board of Sweden, the Region Skåne and the Lund University.

### Pre-registered Clinical Trial Number

The pre-registered clinical trial number for the Carotid Plaque Imaging Project is NCT05821894.
