# Supplementary material for: Spatial transcriptomics reveals a key role of fibroblast-like vascular smooth muscle cells in human atherosclerotic cell crosstalk and stability
Source: Eur Heart J. 2026 Feb 13;47(28):3711–32. doi: 10.1093/eurheartj/ehaf1091 (PMC13384730; doi:10.1093/eurheartj/ehaf1091)
Supplement: ehaf1091_Supplementary_Data [file ehaf1091_supplementary_data.zip › Supl_figures and tables_2026.pdf]

"SUPPLEMENTARY FIGURES & TABLES"

Spatial transcriptomics reveals a key role of fibroblast-like vascular smooth muscle cells in human atherosclerotic cell crosstalk and stability

Isabel Goncalves, MD, PhD[1,2]; Mengyu Pan[1]; Pratibha Singh, PhD[1]; Wenqi Wang[1]; Jing Zhao, PhD[3]; Lea Dib, PhD[4,5]; Lena Sundius[1]; Ana Persson[1]; Chrysostomi Gialeli, PhD[1]; Panagiotis Fountas[1]; Mihaela Nitulescu[1]; Jan Nilsson, MD, PhD[1]; Stephen Malin, PhD[6]; Claudia Monaco, MD, PhD[4]; Helle F Jørgensen, PhD[3]; Jiangming Sun, PhD[1#]; Andreas Edsfeldt, MD, PhD[1,2,7#]

[#]Shared senior authorship

[1]Cardiovascular Research–Translational Studies, Clinical Science Malmö, Lund University, Jan Waldenströms gata 35, SE-214 28, Malmö, Sweden.
[2]Department of Cardiology, University Hospital of Skåne, Jan Waldenströms gata 15, SE-205 02, Sweden.
[3]Section of Cardiorespiratory Medicine, University of Cambridge, VPD Heart and Lung Research Institute, Papworth Road, Cambridge Biomedical Campus, Cambridge, CB2 0BB, UK
[4]Kennedy Institute of Rheumatology, Nuffield Department of Orthopaedics, Rheumatology and Musculoskeletal Sciences, University of Oxford, Oxford, UK
[5]School of Biological Sciences, University of Reading, Berkshire, UK
[6]Department of Medicine Solna, Karolinska Institute, Stockholm, Sweden
[7]Wallenberg Centre for Molecular Medicine, Lund University, Lund, Sweden.

Short title: Spatial cell communications in human plaque microenvironments

Supplementary Table 1. Top 20 differentially expressed genes (DEGs) for each of the six vascular smooth muscle cell (VSMC) clusters identified by spatial transcriptomics.

Gene	P	Avg log2FC	pct.1	pct.2	P_val_adj	Cluster
MYH10	0.00E+00	1.846	0.777	0.306	0.00E+00	VSMC0
RGS5	3.95E-282	1.291	0.841	0.423	6.02E-278	VSMC0
CALD1	2.78E-279	1.283	0.889	0.552	4.24E-275	VSMC0
SPARCL1	9.25E-279	1.981	0.614	0.224	1.41E-274	VSMC0
ITGA8	1.71E-260	1.581	0.705	0.320	2.60E-256	VSMC0
MYH11	4.80E-256	1.461	0.738	0.344	7.30E-252	VSMC0
MAP1B	2.32E-251	1.704	0.693	0.317	3.53E-247	VSMC0
A2M	3.97E-232	1.166	0.870	0.587	6.05E-228	VSMC0
CLU	3.26E-231	1.457	0.600	0.224	4.97E-227	VSMC0
MYL9	6.76E-222	1.032	0.884	0.577	1.03E-217	VSMC0
MFAP4	1.61E-199	1.645	0.526	0.193	2.46E-195	VSMC0
PLN	1.22E-184	1.921	0.418	0.130	1.86E-180	VSMC0
TAGLN	5.15E-180	0.813	0.918	0.674	7.85E-176	VSMC0
ACTN4	3.23E-177	1.198	0.763	0.469	4.92E-173	VSMC0
FRZB	2.98E-175	1.456	0.561	0.241	4.54E-171	VSMC0
CARMN	2.74E-151	1.866	0.383	0.128	4.17E-147	VSMC0
ACTA2	4.49E-150	0.704	0.901	0.629	6.84E-146	VSMC0
FLNA	1.50E-149	0.978	0.750	0.468	2.29E-145	VSMC0
MCAM	3.45E-144	1.421	0.502	0.218	5.26E-140	VSMC0
ADH1B	7.28E-139	1.259	0.562	0.277	1.11E-134	VSMC0
MGP	7.00E-84	0.518	0.975	0.856	1.07E-79	VSMC1
VCAN	4.27E-64	0.521	0.859	0.717	6.50E-60	VSMC1
IGFBP7	1.59E-46	0.324	0.972	0.892	2.42E-42	VSMC1
AEBP1	5.46E-42	0.472	0.803	0.707	8.31E-38	VSMC1
OGN	6.58E-37	0.628	0.493	0.332	1.00E-32	VSMC1
BGN	7.36E-26	0.328	0.825	0.742	1.12E-21	VSMC1
ADH1B	2.55E-24	0.495	0.452	0.320	3.88E-20	VSMC1
EFEMP1	2.74E-24	0.380	0.710	0.602	4.17E-20	VSMC1
CP	1.00E-22	1.034	0.210	0.123	1.53E-18	VSMC1
A2M	6.25E-22	0.246	0.763	0.630	9.52E-18	VSMC1
ECRG4	3.88E-15	0.881	0.177	0.111	5.90E-11	VSMC1
LTBP2	4.23E-15	0.376	0.482	0.388	6.45E-11	VSMC1
FMO2	1.40E-14	0.612	0.215	0.141	2.13E-10	VSMC1
SLC14A1	4.88E-13	0.597	0.226	0.156	7.42E-09	VSMC1
RPS27	1.47E-12	0.290	0.679	0.624	2.23E-08	VSMC1
SFRP2	2.81E-12	0.389	0.362	0.277	4.28E-08	VSMC1
NR2F2	4.88E-12	0.452	0.350	0.273	7.43E-08	VSMC1
MTRNR2L12	6.48E-12	0.420	0.534	0.464	9.87E-08	VSMC1
DEPP1	7.67E-12	0.591	0.240	0.172	1.17E-07	VSMC1
RGS5	1.34E-10	0.135	0.628	0.502	2.04E-06	VSMC1
DCN	5.16E-111	1.128	0.537	0.241	7.86E-107	VSMC2
LUM	1.73E-86	0.724	0.706	0.425	2.64E-82	VSMC2
CD74	2.38E-79	0.655	0.814	0.569	3.63E-75	VSMC2
FTL	1.48E-78	0.518	0.974	0.881	2.25E-74	VSMC2

HLA-DRA	6.58E-73	0.650	0.743	0.498	1.00E-68	VSMC2
CTSB	3.74E-67	0.600	0.816	0.586	5.69E-63	VSMC2
IGKC	3.28E-55	0.877	0.714	0.559	4.99E-51	VSMC2
TIMP3	1.29E-54	0.522	0.445	0.231	1.96E-50	VSMC2
POSTN	1.53E-49	0.451	0.646	0.428	2.33E-45	VSMC2
C1QA	7.10E-49	0.791	0.477	0.277	1.08E-44	VSMC2
HLA-B	2.39E-46	0.481	0.850	0.701	3.64E-42	VSMC2
RNASE1	5.25E-44	0.755	0.447	0.262	7.99E-40	VSMC2
SFRP2	1.70E-42	0.920	0.438	0.267	2.59E-38	VSMC2
SPP1	6.22E-42	0.569	0.674	0.507	9.47E-38	VSMC2
TIMP1	1.66E-40	0.157	0.751	0.566	2.53E-36	VSMC2
JCHAIN	3.28E-40	1.225	0.316	0.169	5.00E-36	VSMC2
B2M	1.13E-36	0.268	0.985	0.954	1.73E-32	VSMC2
COL1A1	2.44E-35	0.445	0.676	0.515	3.72E-31	VSMC2
THBS1	8.67E-35	0.458	0.479	0.304	1.32E-30	VSMC2
COL1A2	1.30E-34	0.304	0.901	0.807	1.98E-30	VSMC2
FN1	6.03E-260	1.339	1.000	0.981	9.18E-256	VSMC3
POSTN	1.24E-216	1.986	0.843	0.417	1.88E-212	VSMC3
COL1A2	4.18E-216	1.415	0.974	0.804	6.36E-212	VSMC3
COL1A1	6.56E-195	1.869	0.865	0.501	9.99E-191	VSMC3
THBS1	2.48E-188	2.239	0.702	0.288	3.77E-184	VSMC3
COL3A1	7.22E-184	1.849	0.835	0.482	1.10E-179	VSMC3
SERPINE1	1.34E-178	2.174	0.698	0.290	2.04E-174	VSMC3
LUM	6.13E-165	1.689	0.818	0.430	9.34E-161	VSMC3
SULF1	1.87E-127	1.560	0.696	0.358	2.85E-123	VSMC3
COL5A1	2.31E-118	1.796	0.480	0.168	3.51E-114	VSMC3
COL5A2	3.87E-106	1.755	0.444	0.155	5.89E-102	VSMC3
COL15A1	7.58E-103	1.624	0.476	0.179	1.15E-98	VSMC3
TIMP1	4.78E-100	1.701	0.823	0.569	7.28E-96	VSMC3
COL4A1	2.43E-98	1.592	0.602	0.306	3.70E-94	VSMC3
FBN1	4.91E-95	1.624	0.482	0.195	7.47E-91	VSMC3
CEMIP	6.73E-95	2.292	0.309	0.088	1.02E-90	VSMC3
TIMP3	5.31E-92	2.079	0.512	0.237	8.09E-88	VSMC3
SERPINE2	6.96E-83	2.295	0.324	0.105	1.06E-78	VSMC3
TNFRSF11B	3.69E-79	1.552	0.550	0.288	5.61E-75	VSMC3
PRSS23	2.01E-69	1.242	0.574	0.316	3.07E-65	VSMC3
CTSB	3.21E-284	1.875	0.975	0.586	4.88E-280	VSMC4
FTL	3.65E-264	1.541	0.994	0.885	5.56E-260	VSMC4
CD74	1.86E-251	1.765	0.933	0.574	2.84E-247	VSMC4
HLA-DRA	6.76E-228	1.839	0.897	0.499	1.03E-223	VSMC4
RNASE1	9.82E-209	2.134	0.702	0.246	1.50E-204	VSMC4
TIMP1	6.42E-205	1.745	0.925	0.560	9.78E-201	VSMC4
LAPTM5	9.01E-203	1.982	0.665	0.213	1.37E-198	VSMC4
TNC	8.53E-195	2.115	0.622	0.189	1.30E-190	VSMC4
LYZ	1.96E-188	2.081	0.612	0.188	2.99E-184	VSMC4
ITGB2	4.72E-187	2.118	0.565	0.156	7.19E-183	VSMC4
IFI30	9.84E-183	2.163	0.518	0.133	1.50E-178	VSMC4
CD68	1.45E-179	2.082	0.535	0.144	2.21E-175	VSMC4

HLA-DRB1	1.54E-175	1.943	0.692	0.269	2.34E-171	VSMC4
TIMP3	1.74E-174	1.859	0.641	0.224	2.64E-170	VSMC4
FTH1	1.24E-169	1.263	0.973	0.816	1.89E-165	VSMC4
CD14	9.48E-166	2.125	0.519	0.146	1.44E-161	VSMC4
PSAP	1.47E-164	1.273	0.917	0.643	2.23E-160	VSMC4
TMSB10	1.71E-164	1.246	0.918	0.579	2.61E-160	VSMC4
APOE	1.23E-163	2.067	0.590	0.198	1.87E-159	VSMC4
CTSD	1.51E-161	1.370	0.900	0.607	2.30E-157	VSMC4
ACTA2	4.21E-214	1.476	0.961	0.669	6.42E-210	VSMC5
TPM2	2.01E-165	1.461	0.843	0.456	3.07E-161	VSMC5
DSTN	6.10E-130	1.136	0.878	0.552	9.29E-126	VSMC5
MYL9	3.44E-124	1.049	0.910	0.628	5.24E-120	VSMC5
TAGLN	3.39E-121	0.936	0.954	0.712	5.16E-117	VSMC5
IGFBP7	8.13E-106	0.674	0.995	0.901	1.24E-101	VSMC5
CRIP1	1.52E-84	1.362	0.615	0.323	2.31E-80	VSMC5
FHL1	1.32E-82	1.091	0.732	0.431	2.01E-78	VSMC5
FLNA	2.46E-81	0.984	0.797	0.511	3.75E-77	VSMC5
RGS5	2.78E-77	1.011	0.785	0.502	4.23E-73	VSMC5
ADIRF	5.67E-73	1.237	0.650	0.365	8.63E-69	VSMC5
MCAM	8.27E-67	1.093	0.543	0.263	1.26E-62	VSMC5
INHBA	9.95E-67	1.263	0.521	0.259	1.51E-62	VSMC5
MYH11	3.04E-62	0.834	0.711	0.416	4.63E-58	VSMC5
ANTXR1	4.24E-62	1.141	0.539	0.278	6.45E-58	VSMC5
PPP1R14A	8.13E-62	1.340	0.431	0.192	1.24E-57	VSMC5
NDUFA4L2	3.17E-59	1.495	0.386	0.165	4.83E-55	VSMC5
CSRP1	1.16E-41	0.955	0.475	0.259	1.77E-37	VSMC5
MGP	6.51E-41	0.376	0.986	0.872	9.92E-37	VSMC5
MYH9	1.38E-38	0.760	0.637	0.432	2.11E-34	VSMC5

P: unadjusted p-value from a Wilcoxon rank sum test; Avg_log2FC: log2-transofrmed fold-change of the average expression between each studied Vascular Smooth Muscle Cell (VSMC) cluster and all the rest VSMC clusters; pct.1: The percentage of cells where the gene is detected in the studied VSMC cluster; pct.2: The percentage of cells where the gene is detected in the all the rest VSMC clusters ; P_val_adj: Adjusted p-value, based on Bonferroni correction using all the detected genes in the data. Cluster: the studied VSMC cluster.

Supplementary Table 2. Top 20 differentially expressed genes (DEGs) for each of the six macrophage (MO) clusters identified by spatial transcriptomics.

Gene	P	Avg_log2FC	pct.1	pct.2	P_val_adj	Cluster
MALAT1	1.47E-152	0.9	0.95	0.84	2.33E-148	MO0
MGP	5.90E-97	0.7	0.79	0.56	9.36E-93	MO0
MTRNR2L12	5.80E-76	1.21	0.63	0.45	9.21E-72	MO0
NEAT1	2.86E-65	0.71	0.71	0.54	4.54E-61	MO0
COL1A2	4.02E-53	0.53	0.71	0.53	6.38E-49	MO0
MTRNR2L8	4.30E-53	0.94	0.49	0.33	6.82E-49	MO0
IGFBP7	2.02E-51	0.52	0.71	0.54	3.21E-47	MO0
FN1	5.58E-51	0.29	0.98	0.86	8.86E-47	MO0
VCAN	2.31E-50	0.69	0.6	0.44	3.67E-46	MO0
IGFBP5	1.69E-39	0.53	0.45	0.29	2.68E-35	MO0
RGS5	3.78E-38	0.95	0.3	0.17	5.99E-34	MO0
CCN2	4.11E-36	0.51	0.61	0.46	6.52E-32	MO0
CALD1	3.97E-24	0.64	0.36	0.25	6.30E-20	MO0
ACTA2	2.80E-21	0.5	0.43	0.32	4.44E-17	MO0
LTBP1	5.30E-20	0.76	0.26	0.17	8.42E-16	MO0
TAGLN	3.01E-17	0.46	0.45	0.36	4.77E-13	MO0
AL627171.2	8.93E-17	0.73	0.29	0.2	1.42E-12	MO0
CLU	1.11E-15	0.62	0.2	0.13	1.77E-11	MO0
CCN3	1.87E-15	0.77	0.18	0.11	2.96E-11	MO0
LPP	2.01E-15	0.64	0.26	0.18	3.19E-11	MO0
MMP9	3.71E-248	1.47	0.81	0.39	5.88E-244	MO1
CSTB	1.62E-179	1.11	0.85	0.55	2.57E-175	MO1
SPP1	1.67E-171	0.94	0.99	0.8	2.65E-167	MO1
MMP12	1.26E-141	1.64	0.64	0.33	2.00E-137	MO1
FTH1	3.97E-134	0.66	1	0.98	6.30E-130	MO1
CD36	1.72E-127	1.46	0.56	0.26	2.73E-123	MO1
CTSB	1.28E-111	0.61	0.99	0.95	2.03E-107	MO1
SDC2	2.30E-95	1.15	0.54	0.27	3.65E-91	MO1
CTSL	7.50E-95	1	0.73	0.51	1.19E-90	MO1
PLIN2	1.28E-88	1.41	0.5	0.26	2.04E-84	MO1
ADAM8	1.90E-86	1.52	0.34	0.13	3.01E-82	MO1
S100A10	1.56E-78	0.87	0.67	0.43	2.48E-74	MO1
VIM	3.54E-78	0.53	0.94	0.83	5.62E-74	MO1
MMP7	5.40E-74	1.21	0.35	0.15	8.57E-70	MO1
GAPDH	3.34E-66	0.69	0.76	0.58	5.29E-62	MO1
CHI3L1	4.90E-66	0.89	0.38	0.17	7.78E-62	MO1
SCD	8.75E-59	1.18	0.38	0.2	1.39E-54	MO1
LAPTM5	4.90E-58	0.58	0.79	0.62	7.78E-54	MO1
CD52	1.60E-56	0.91	0.37	0.19	2.54E-52	MO1
PLAUR	1.02E-55	1.03	0.37	0.18	1.62E-51	MO1
CD74	2.37E-142	0.78	0.98	0.89	3.77E-138	MO2
HLA-DRA	4.08E-124	0.77	0.96	0.85	6.48E-120	MO2
C1QA	1.59E-81	0.65	0.79	0.54	2.53E-77	MO2
HLA-DRB1	1.79E-76	0.86	0.78	0.57	2.84E-72	MO2

HLA-DPA1	2.37E-68	0.84	0.7	0.48	3.75E-64	MO2
HLA-DPB1	7.19E-68	0.85	0.66	0.43	1.14E-63	MO2
FCGR2A	1.84E-62	0.99	0.53	0.32	2.92E-58	MO2
C1QB	5.54E-58	0.51	0.72	0.48	8.79E-54	MO2
HLA-DQB1	9.15E-50	0.86	0.52	0.32	1.45E-45	MO2
SELENOP	1.42E-39	0.4	0.59	0.39	2.25E-35	MO2
HLA-DQA1	2.89E-38	0.84	0.4	0.23	4.59E-34	MO2
CD14	1.35E-35	0.55	0.57	0.39	2.15E-31	MO2
C1QC	1.54E-31	0.61	0.41	0.26	2.44E-27	MO2
STAB1	3.29E-31	0.81	0.4	0.25	5.22E-27	MO2
FCGR3A	4.41E-29	0.62	0.48	0.33	7.00E-25	MO2
MSR1	3.60E-28	0.69	0.47	0.33	5.71E-24	MO2
CTSD	5.55E-26	0.29	0.95	0.93	8.81E-22	MO2
MS4A6A	9.27E-26	0.6	0.36	0.22	1.47E-21	MO2
ALOX5AP	1.25E-25	1.08	0.21	0.11	1.98E-21	MO2
C3AR1	2.23E-23	0.77	0.31	0.19	3.54E-19	MO2
COL1A2	1.52E-149	1.17	0.86	0.53	2.41E-145	MO3
COL3A1	2.19E-130	1.54	0.69	0.35	3.47E-126	MO3
BGN	8.96E-125	1.32	0.69	0.35	1.42E-120	MO3
AEBP1	1.09E-115	1.38	0.63	0.31	1.74E-111	MO3
POSTN	4.30E-113	1.51	0.69	0.4	6.82E-109	MO3
THBS2	8.75E-107	1.27	0.61	0.29	1.39E-102	MO3
COL1A1	1.01E-104	1.39	0.69	0.4	1.60E-100	MO3
IGFBP7	2.52E-103	1.02	0.84	0.55	4.00E-99	MO3
LUM	4.11E-101	1.36	0.64	0.35	6.53E-97	MO3
SPARC	3.42E-97	1.05	0.74	0.44	5.42E-93	MO3
EFEMP1	1.04E-94	1.19	0.61	0.3	1.66E-90	MO3
C1R	2.33E-91	1.39	0.48	0.21	3.70E-87	MO3
FN1	9.17E-90	0.67	0.98	0.88	1.46E-85	MO3
SULF1	1.84E-87	1.26	0.5	0.23	2.93E-83	MO3
TAGLN	1.86E-84	1.11	0.62	0.34	2.95E-80	MO3
CCN2	2.28E-84	1.01	0.74	0.46	3.62E-80	MO3
CCDC80	5.97E-81	1.25	0.49	0.23	9.47E-77	MO3
IFITM3	4.06E-79	1.23	0.52	0.26	6.44E-75	MO3
TIMP1	6.34E-78	1.09	0.84	0.64	1.01E-73	MO3
MGP	1.74E-70	0.98	0.81	0.6	2.76E-66	MO3
SELENOP	2.15E-239	2.19	0.88	0.38	3.41E-235	MO4
C1QB	1.37E-234	1.92	0.92	0.48	2.18E-230	MO4
FUCA1	1.45E-200	2.15	0.68	0.21	2.31E-196	MO4
SLC40A1	1.39E-192	2.58	0.67	0.22	2.21E-188	MO4
C1QA	1.89E-179	1.62	0.92	0.55	3.00E-175	MO4
CCL18	1.03E-161	2.36	0.76	0.37	1.64E-157	MO4
CTSC	3.31E-147	1.98	0.67	0.26	5.25E-143	MO4
MS4A6A	3.02E-143	1.88	0.61	0.21	4.80E-139	MO4
CPVL	3.27E-143	1.9	0.58	0.19	5.20E-139	MO4
FOLR2	6.79E-143	2.15	0.48	0.13	1.08E-138	MO4
LGMN	1.71E-129	1.45	0.81	0.46	2.72E-125	MO4
CD74	4.94E-128	0.89	0.99	0.9	7.83E-124	MO4

C1QC	7.19E-127	1.81	0.64	0.25	1.14E-122	MO4
ALDH1A1	8.35E-125	2.35	0.34	0.07	1.32E-120	MO4
HLA-DPB1	6.82E-118	1.24	0.82	0.44	1.08E-113	MO4
MS4A4A	5.34E-111	1.67	0.55	0.2	8.48E-107	MO4
CFD	2.05E-106	1.77	0.51	0.18	3.25E-102	MO4
F13A1	1.69E-104	2.1	0.5	0.18	2.68E-100	MO4
HLA-DPA1	9.59E-103	1.09	0.83	0.49	1.52E-98	MO4
S100A9	2.03E-98	1.66	0.58	0.25	3.23E-94	MO4
RGCC	2.20E-240	3.12	0.82	0.17	3.49E-236	MO5
CHI3L1	3.59E-226	3.43	0.83	0.19	5.70E-222	MO5
MMP9	1.49E-204	2.89	0.99	0.45	2.36E-200	MO5
MMP7	6.90E-187	3.2	0.74	0.16	1.10E-182	MO5
LPL	2.92E-180	3.14	0.54	0.08	4.64E-176	MO5
GPC4	8.54E-180	3.49	0.44	0.05	1.36E-175	MO5
CD52	1.61E-178	2.87	0.78	0.2	2.55E-174	MO5
IL1RN	1.26E-173	3.16	0.66	0.13	1.99E-169	MO5
FBP1	2.17E-168	2.91	0.59	0.1	3.45E-164	MO5
MMP19	1.25E-161	2.71	0.61	0.12	1.99E-157	MO5
CSTB	6.15E-141	1.75	0.99	0.6	9.75E-137	MO5
TM4SF19	1.09E-132	3.31	0.34	0.04	1.73E-128	MO5
TFRC	3.53E-128	2.74	0.63	0.16	5.60E-124	MO5
GAL	1.78E-119	4.04	0.24	0.02	2.83E-115	MO5
CIR1	1.39E-118	2.59	0.63	0.17	2.21E-114	MO5
EMP1	3.88E-113	2.41	0.59	0.15	6.15E-109	MO5
CD109	7.00E-113	2.24	0.56	0.13	1.11E-108	MO5
ANPEP	1.03E-111	2.32	0.56	0.13	1.63E-107	MO5
S100A10	1.02E-110	1.7	0.9	0.46	1.62E-106	MO5
MFSD12	9.20E-105	2.43	0.49	0.11	1.46E-100	MO5

P: unadjusted p-value from a Wilcoxon rank sum test; Avg_log2FC: log2-transofrmed fold-change of the average expression between each studied macrophage (MO) cluster and all the rest MO clusters; pct.1: The percentage of cells where the gene is detected in the studied MO cluster; pct.2: The percentage of cells where the gene is detected in the all the rest MO clusters ; P_val_adj: Adjusted p-value, based on Bonferroni correction using all the detected genes in the data. Cluster: the studied MO cluster.

Supplementary Table 3. Top 20 differentially expressed genes (DEGs) for each of the three B cell clusters identified by spatial transcriptomics.

Gene	P	Avg_log2FC	pct.1	pct.2	P_val_adj	Cluster
IGKC	2.59E-44	0.572	0.990	0.975	4.75E-40	B cells0
IGHG3	3.49E-36	0.374	0.896	0.699	6.39E-32	B cells0
JCHAIN	1.13E-25	0.433	0.848	0.661	2.06E-21	B cells0
IGHG4	1.77E-24	0.380	0.777	0.637	3.24E-20	B cells0
IGLC3	5.66E-20	0.236	0.599	0.431	1.04E-15	B cells0
IGHG1	1.06E-17	0.176	0.769	0.603	1.94E-13	B cells0
IGHGP	1.55E-16	0.127	0.497	0.324	2.84E-12	B cells0
IGHA1	1.76E-15	0.364	0.775	0.671	3.22E-11	B cells0
VWF	8.07E-10	0.249	0.268	0.167	1.48E-05	B cells0
FOS	1.44E-09	0.208	0.308	0.204	2.64E-05	B cells0
TXNDC5	1.92E-09	0.150	0.537	0.389	3.52E-05	B cells0
SSR4	2.83E-09	0.106	0.566	0.421	5.18E-05	B cells0
PECAM1	4.19E-09	0.143	0.310	0.202	7.69E-05	B cells0
IGLC7	1.07E-08	0.274	0.143	0.073	0.000196	B cells0
CLTC	8.69E-08	0.110	0.298	0.201	0.001594	B cells0
COTL1	1.10E-07	0.114	0.288	0.191	0.002021	B cells0
MS4A6A	3.57E-07	0.133	0.269	0.182	0.006542	B cells0
HLA-DMA	5.36E-07	0.105	0.213	0.136	0.009817	B cells0
SLCO2B1	1.57E-06	0.101	0.274	0.188	0.028704	B cells0
SELENOP	1.77E-06	0.195	0.586	0.484	0.032385	B cells0
FN1	2.72E-50	0.605	0.988	0.918	4.98E-46	B cells1
SPP1	2.35E-34	0.898	0.810	0.650	4.31E-30	B cells1
COL1A2	4.02E-31	0.499	0.892	0.739	7.37E-27	B cells1
FTH1	1.46E-29	0.439	0.961	0.910	2.68E-25	B cells1
CTSB	3.55E-29	0.391	0.932	0.822	6.51E-25	B cells1
VIM	6.43E-26	0.381	0.912	0.779	1.18E-21	B cells1
TIMP1	3.27E-25	0.404	0.847	0.685	6.00E-21	B cells1
CTSD	1.17E-24	0.325	0.929	0.762	2.15E-20	B cells1
VCAN	3.12E-23	0.434	0.770	0.619	5.72E-19	B cells1
FTL	2.62E-21	0.339	0.995	0.976	4.81E-17	B cells1
THBS2	1.20E-18	0.364	0.631	0.480	2.20E-14	B cells1
NEAT1	6.41E-17	0.293	0.795	0.650	1.17E-12	B cells1
COL1A1	1.25E-16	0.356	0.681	0.536	2.28E-12	B cells1
SERPINE1	2.22E-16	0.335	0.409	0.265	4.06E-12	B cells1
MFGE8	3.14E-16	0.357	0.486	0.345	5.76E-12	B cells1
MALAT1	1.23E-15	0.258	0.951	0.893	2.26E-11	B cells1
SPARC	2.32E-15	0.356	0.750	0.649	4.26E-11	B cells1
MGP	4.28E-15	0.527	0.914	0.811	7.85E-11	B cells1
BGN	1.13E-14	0.410	0.719	0.619	2.06E-10	B cells1
RGS5	1.83E-14	0.311	0.476	0.336	3.35E-10	B cells1
XBP1	1.29E-84	1.672	0.985	0.346	2.36E-80	B cells2
MZB1	6.19E-81	1.735	0.934	0.269	1.13E-76	B cells2
TENT5C	8.89E-78	0.874	0.818	0.169	1.63E-73	B cells2

DERL3	2.64E-75	1.248	0.839	0.209	4.83E-71	B cells2
SDC1	3.02E-75	0.692	0.672	0.112	5.53E-71	B cells2
PIM2	9.85E-75	0.987	0.847	0.200	1.81E-70	B cells2
FKBP11	1.87E-69	1.131	0.796	0.202	3.44E-65	B cells2
SEC11C	3.05E-65	1.053	0.847	0.234	5.59E-61	B cells2
IGHG2	3.98E-64	2.322	0.825	0.247	7.30E-60	B cells2
IGHG3	7.72E-62	2.310	0.978	0.793	1.41E-57	B cells2
CD79A	9.59E-62	0.913	0.672	0.144	1.76E-57	B cells2
SSR4	1.74E-60	1.589	0.985	0.470	3.18E-56	B cells2
TXNDC5	2.32E-57	1.306	0.971	0.439	4.25E-53	B cells2
FCRL5	4.82E-56	0.540	0.526	0.088	8.83E-52	B cells2
PRDX4	1.03E-53	0.740	0.781	0.209	1.89E-49	B cells2
IGHGP	4.16E-53	2.532	0.883	0.388	7.62E-49	B cells2
IGHG1	2.38E-51	2.177	0.978	0.674	4.35E-47	B cells2
PTP4A3	1.36E-50	0.617	0.628	0.141	2.49E-46	B cells2
POU2AF1	4.40E-47	0.514	0.482	0.088	8.06E-43	B cells2
HERPUD1	1.13E-46	0.819	0.745	0.231	2.08E-42	B cells2

P: unadjusted p-value from a Wilcoxon rank sum test; Avg_log2FC: log2-transofrmed fold-change of the average expression between each studied B-cell cluster and all the rest B-cell clusters; pct.1: The percentage of cells where the gene is detected in the studied B-cell cluster; pct.2: The percentage of cells where the gene is detected in the all the rest B-cell clusters ; P_val_adj: Adjusted p-value, based on Bonferroni correction using all the detected genes in the data. Cluster: the studied B cells cluster.

Supplementary Table 4. Top 20 differentially expressed genes (DEGs) for the two endothelial cell (EC) clusters identified by spatial transcriptomics.

Gene	P	Avg_log2FC	pct.1	pct.2	P_val_adj	Cluster
MGP	6.30E-36	1.099	0.933	0.705	1.16E-31	EC0
BGN	3.82E-34	0.843	0.791	0.476	7.01E-30	EC0
VCAN	6.50E-34	0.875	0.772	0.444	1.19E-29	EC0
FN1	1.11E-27	0.699	0.985	0.917	2.03E-23	EC0
AEBP1	2.53E-25	0.697	0.724	0.390	4.65E-21	EC0
COL1A2	9.99E-25	0.709	0.890	0.683	1.83E-20	EC0
COL8A1	1.79E-23	0.595	0.390	0.105	3.29E-19	EC0
THBS2	2.25E-23	0.607	0.595	0.290	4.13E-19	EC0
CALD1	2.02E-21	0.558	0.629	0.339	3.70E-17	EC0
EFEMP1	7.55E-21	0.711	0.720	0.456	1.38E-16	EC0
OGN	1.30E-17	0.524	0.394	0.141	2.38E-13	EC0
PTGIS	5.49E-17	0.416	0.336	0.105	1.01E-12	EC0
CCDC80	5.67E-17	0.524	0.569	0.290	1.04E-12	EC0
MYH10	1.70E-16	0.390	0.312	0.090	3.11E-12	EC0
LTBP2	2.22E-15	0.437	0.461	0.229	4.07E-11	EC0
TAGLN	6.28E-15	0.491	0.694	0.454	1.15E-10	EC0
SULF1	1.41E-14	0.575	0.517	0.285	2.58E-10	EC0
ELN	1.60E-14	0.517	0.280	0.083	2.94E-10	EC0
NEAT1	1.66E-14	0.525	0.761	0.554	3.04E-10	EC0
IGFBP6	2.29E-14	0.329	0.276	0.078	4.20E-10	EC0
SELENOP	9.73E-28	0.904	0.756	0.487	1.78E-23	EC1
LGMN	1.48E-21	0.640	0.527	0.246	2.72E-17	EC1
C1QA	1.05E-20	0.874	0.761	0.526	1.92E-16	EC1
HMOX1	1.11E-19	0.769	0.432	0.177	2.04E-15	EC1
RPS28	4.81E-19	0.440	0.837	0.629	8.81E-15	EC1
TMSB10	1.34E-18	0.390	0.990	0.948	2.46E-14	EC1
FKBP1A	4.28E-18	0.386	0.622	0.351	7.84E-14	EC1
FUCA1	4.53E-18	0.445	0.356	0.121	8.29E-14	EC1
CTSC	7.33E-18	0.462	0.551	0.287	1.34E-13	EC1
RPS2	1.47E-17	0.384	0.912	0.754	2.70E-13	EC1
PRCP	2.34E-17	0.414	0.498	0.241	4.29E-13	EC1
RPL36A	1.50E-16	0.360	0.807	0.534	2.74E-12	EC1
RPL39	7.56E-16	0.355	0.944	0.821	1.39E-11	EC1
ACKR1	9.08E-16	0.392	0.288	0.084	1.67E-11	EC1
CCL18	9.18E-16	0.962	0.410	0.188	1.68E-11	EC1
CCL14	1.21E-15	0.543	0.476	0.228	2.21E-11	EC1
HLA-A	1.33E-15	0.363	0.963	0.881	2.43E-11	EC1
RPL41	1.38E-15	0.370	0.980	0.918	2.53E-11	EC1
CD163	2.53E-15	0.537	0.480	0.239	4.64E-11	EC1
RPLP0	2.95E-15	0.366	0.846	0.666	5.41E-11	EC1

P: unadjusted p-value from a Wilcoxon rank sum test; Avg_log2FC: log2-transofrmed fold-change of the average expression between each studied endothelial cell (EC) cluster and another EC cluster; pct.1: The percentage of cells where the gene is detected in the studied EC

cluster; pct.2: The percentage of cells where the gene is detected in the another EC cluster ; P_val_adj: Adjusted p-value, based on Bonferroni correction using all the detected genes in the data. Cluster: the studied EC cluster.

Supplementary Table 5. 38 candidate therapies screened from LINCS L1000 targeting VSMC3 markers.

Drug	Combined Score	Odds Ratio	Adjusted P-value
Hydroxyurea	316.780	20.547	5.03E-06
Zidovudine	212.983	17.076	5.51E-05
Dobutamine	210.402	16.933	5.51E-05
Lacidipine	138.310	14.114	4.67E-04
Flunarizine	135.703	13.933	4.67E-04
Tacrolimus	135.703	13.933	4.67E-04
Oxybutynin	133.173	13.756	4.67E-04
Allopurinol	132.346	13.698	4.67E-04
Esmolol	130.717	13.583	4.67E-04
Losartan	130.717	13.583	4.67E-04
Neomycin	129.914	13.527	4.67E-04
Valdecoxib	80.128	11.072	3.52E-03
Bisphenol-A	74.619	10.560	3.52E-03
Leflunomide	74.619	10.560	3.52E-03
Amikacin	74.148	10.516	3.52E-03
Methotrexate	74.148	10.516	3.52E-03
Procainamide	74.148	10.516	3.52E-03
Calcitriol	73.681	10.472	3.52E-03
Daunorubicin	73.681	10.472	3.52E-03
Doxifluridine	73.681	10.472	3.52E-03
Stavudine	73.681	10.472	3.52E-03
Carmustine	73.218	10.429	3.52E-03
Cytarabine	73.218	10.429	3.52E-03
Oxymetazoline	73.218	10.429	3.52E-03
Sulindac	72.761	10.386	3.52E-03
Angiogenesis-Inhibitor	37.533	7.837	2.18E-02
Acyclovir	37.037	7.771	2.18E-02
Azathioprine	36.793	7.738	2.18E-02
Mitomycin	36.793	7.738	2.18E-02
Mycophenolate-Mofetil	36.075	7.641	2.18E-02
Citalopram	35.841	7.609	2.18E-02
Nevirapine	35.841	7.609	2.18E-02
Ifosfamide	35.608	7.577	2.18E-02
Iproniazid	35.608	7.577	2.18E-02
Mevastatin	35.608	7.577	2.18E-02
Aspirin	35.151	7.515	2.18E-02
Gallamine	35.151	7.515	2.18E-02
Ketoprofen	35.151	7.515	2.18E-02

Adjusted P-value: Benjamini-Hochberg (BH)-adjusted p-values for the enriched compounds.

Supplementary Figure 1

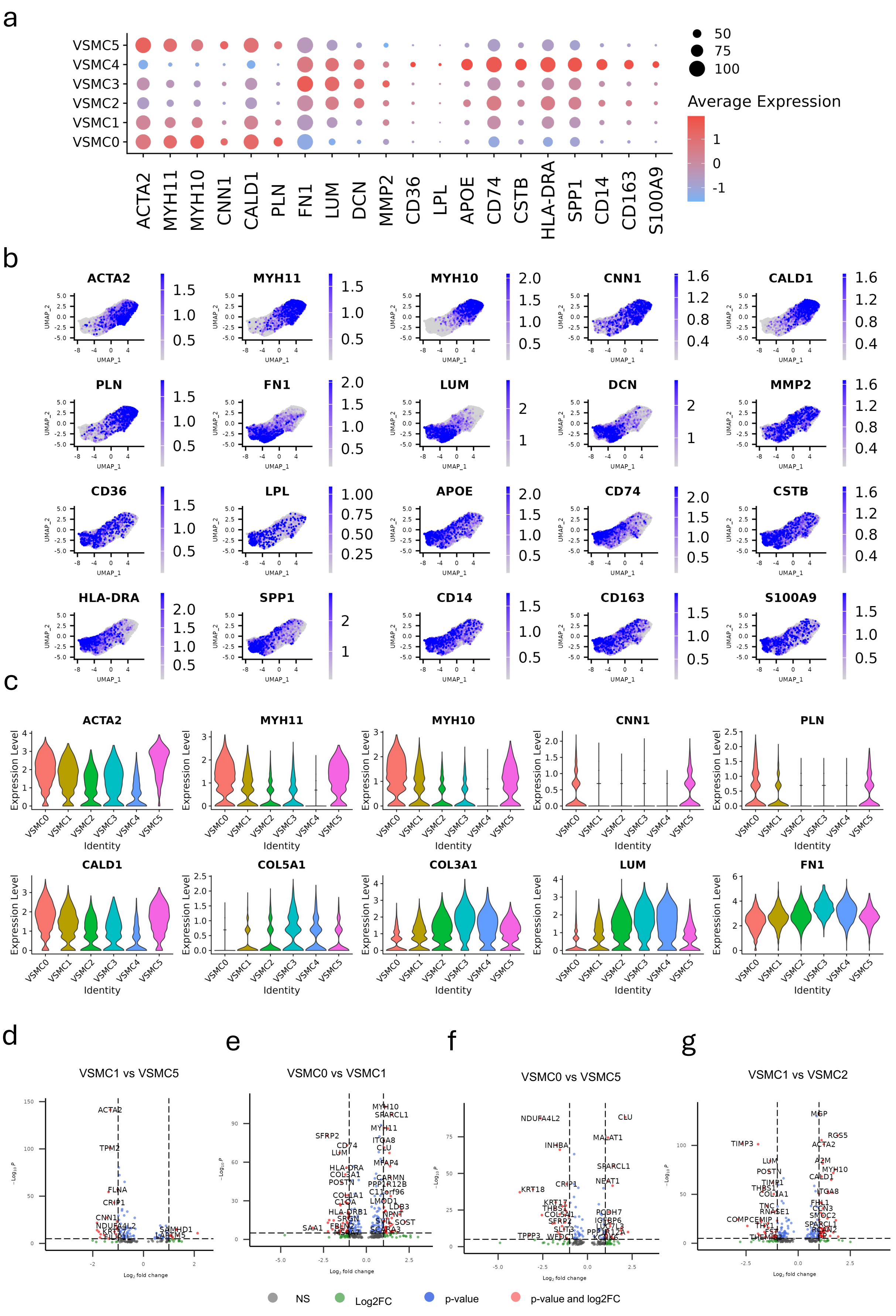

Supplementary Figure 1. Gene expression in the six identified vascular smooth muscle cell (VSMC) clusters. Gene expression is visualized using **a)** dot plot, **b)** feature plot over a Uniform Manifold Approximation and Projection (UMAP) dimensionality reduction, and **c)** violin plot. In the dot plot, color represents the average expression level of each gene within VSMC clusters, while the dot size indicates the percentage of cells expressing the gene in each VSMC cluster. Volcano plots are showing differentially expressed genes between **d)** VSMC1 and VSMC5, **e)** VSMC0 and VSMC1, **f)** VSMC0 and VSMC5, and **g)** VSMC1 and VSMC2.

Supplementary Figure 2

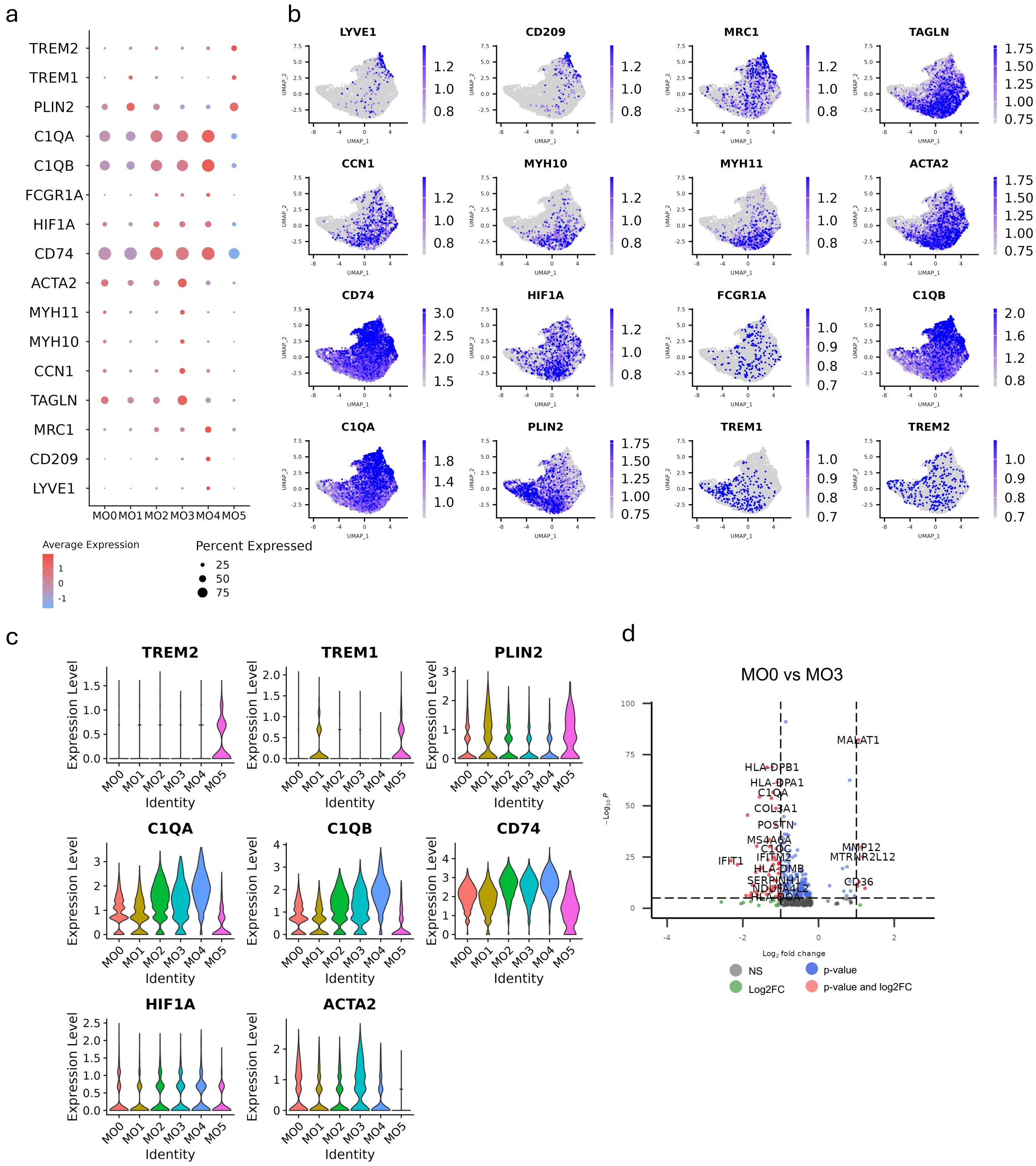

Supplementary Figure 2. Gene expression of marker genes in the six identified macrophage (MO) clusters. Marker gene expression visualized by **a)** dot plot, **b)** feature plot over a Uniform Manifold Approximation and Projection (UMAP) dimensionality reduction, and **c)** violin plot. In the dot plot, the color represents the average expression level of each gene within MO clusters, while the dot size indicates the percentage of cells expressing the gene in each MO cluster. **d)** Volcano plot showing differentially expressed genes between MO0 and MO3.

Supplementary Figure 3

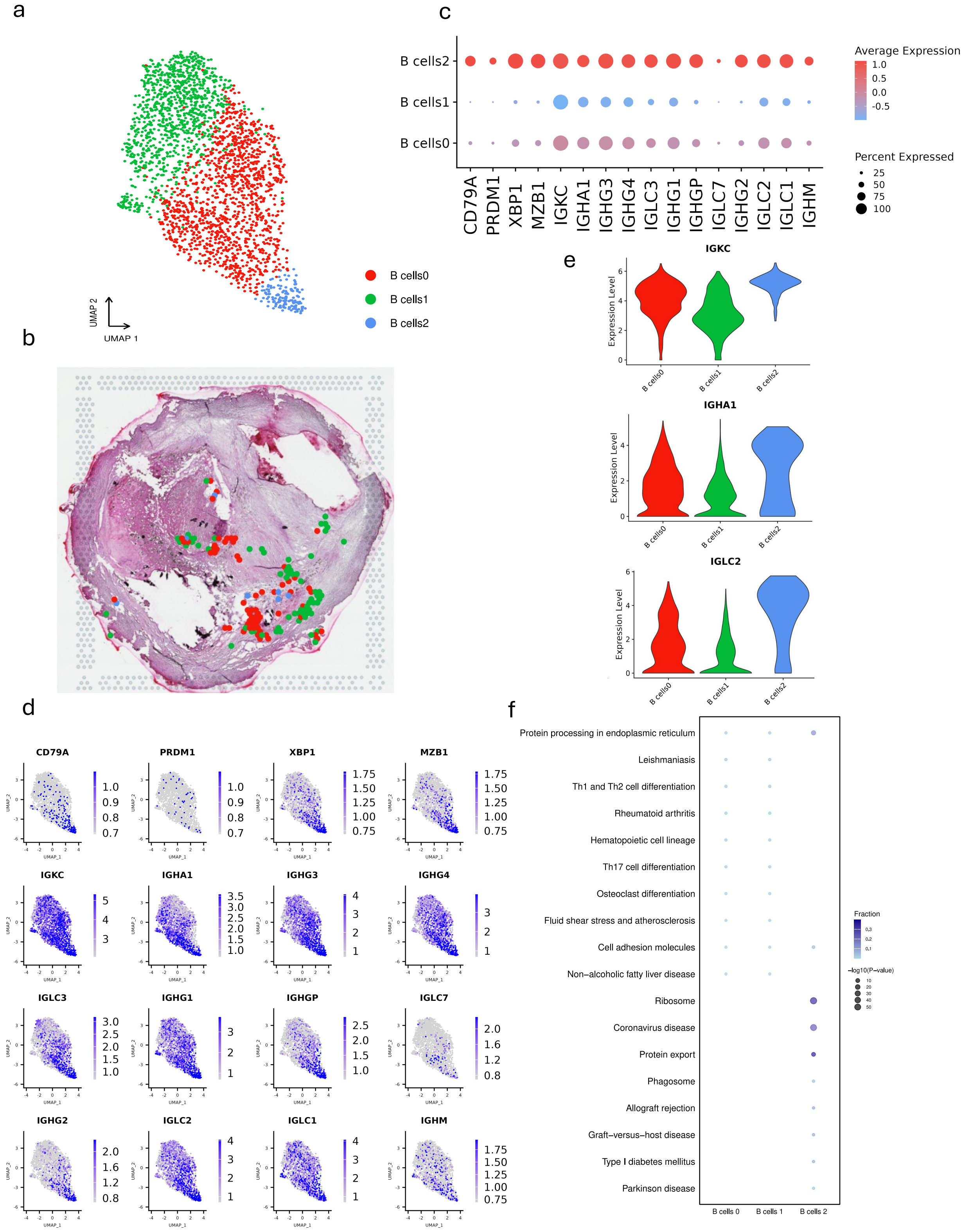

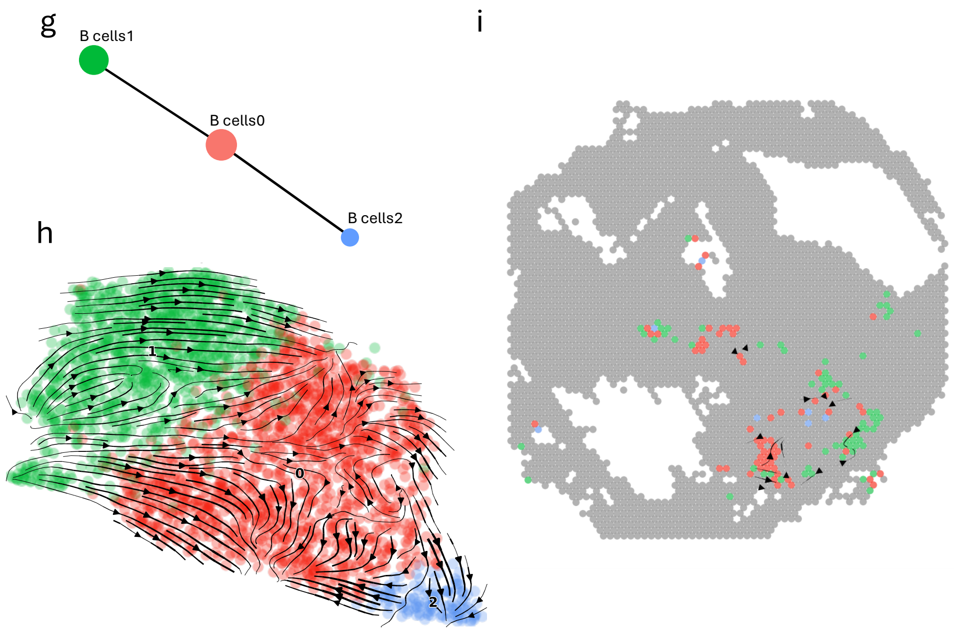

Supplementary Figure 3. B-cell subtypes identified by spatial transcriptomic analysis of human carotid plaques. **a)** UMAP plot of the three identified B cells subclusters in 2,539 spots from 13 plaques. Based on common B cells marker gene expression, subclusters B cells0 (intermediate plasma cells), B cells1 (mixed cell type) and B cells2 (plasma B cells) were annotated. **b)** Spatial distribution of the three B cell subclusters visualized on a representative plaque tissue section. Gene expression visualized by **c)** dot plot, **d)** feature plot over a Uniform Manifold Approximation and Projection (UMAP) dimensionality reduction, and **e)** violin plot. In the dot plot, the color represents the average expression level of each gene within B cell clusters, while the dot size indicates the percentage of cells expressing the gene in each B cell cluster. **f)** Heatmap showing the top 10 enriched pathways by p-value for each B cell subcluster. **g)** Partition-based graph abstraction (PAGA) graph showing connectivity among the three B cell subclusters, with line thickness indicating connection strength. **h)** RNA velocity estimated cellular state transitions between B cell subclusters. The stream plot of RNA velocities overlaid on a UMAP plot. **i)** RNA velocity stream mapping to spatial location on a plaque tissue section.

Supplementary Figure 4

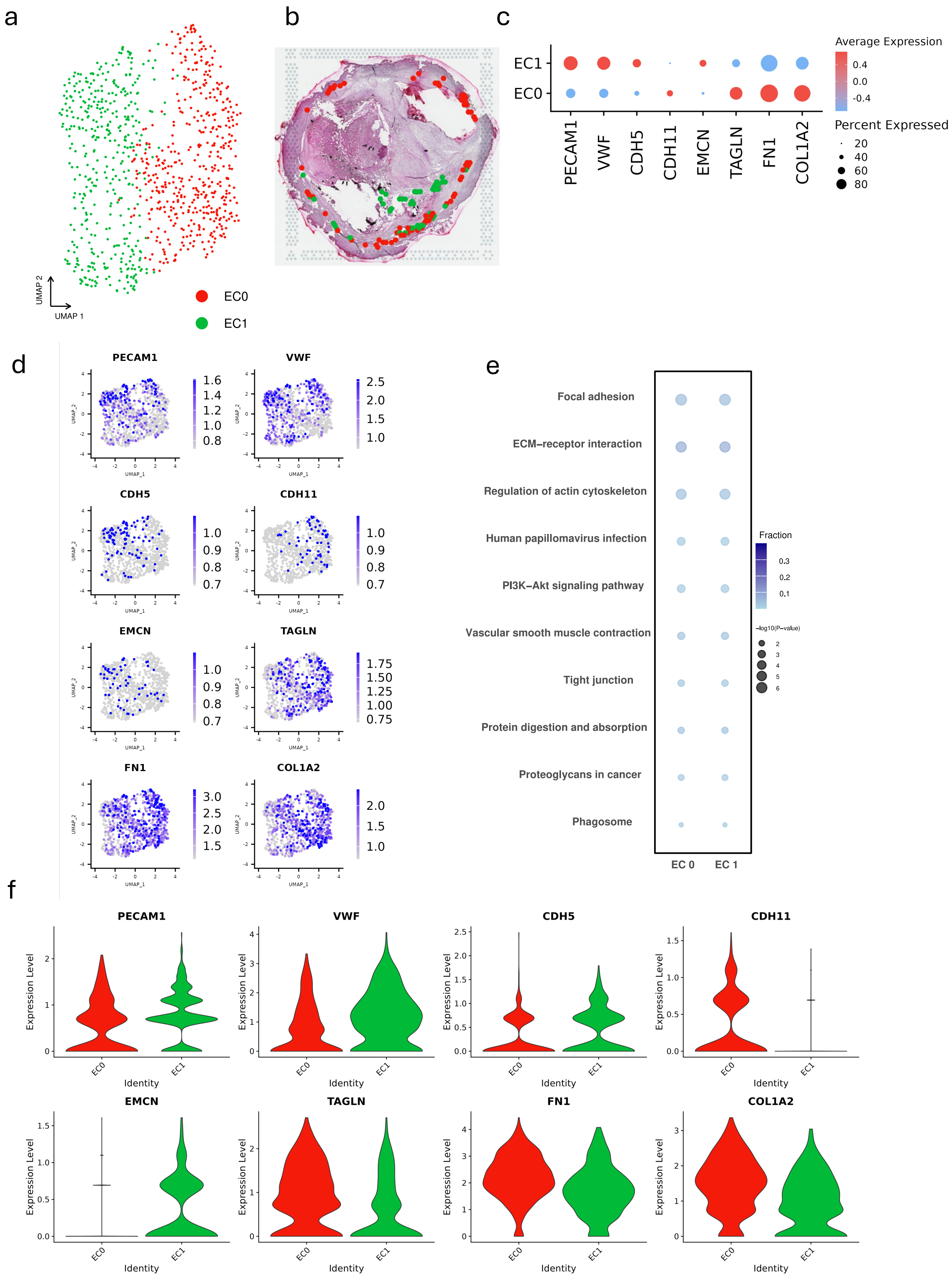

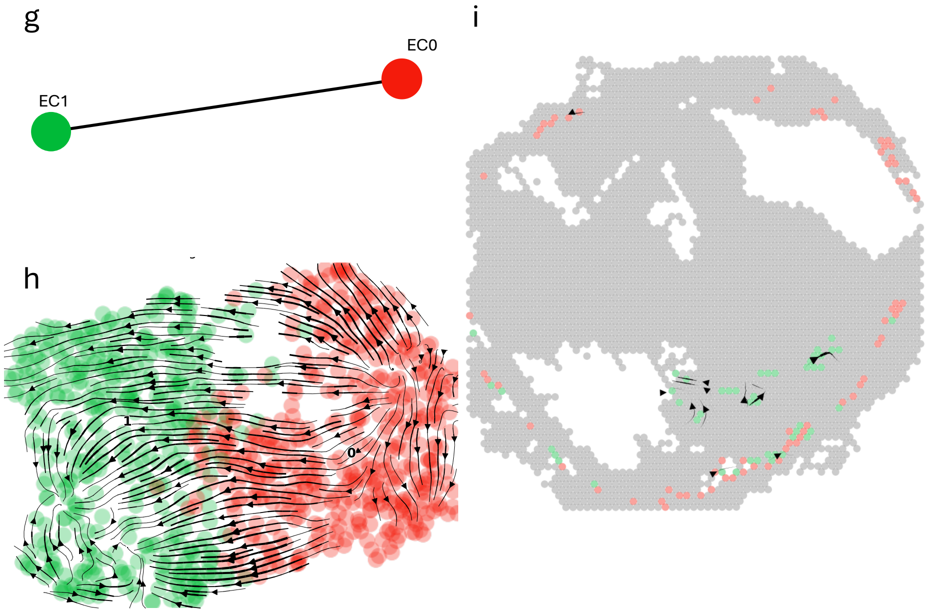

Supplementary Figure 4. Endothelial cells subtypes identified by spatial transcriptomic analysis of human carotid plaques. a) UMAP plot of the two identified endothelial cells subclusters in 874 spots from 13 plaques. Based on common endothelial cells (EC) marker gene expression, subclusters EC0 (endothelial-mesenchymal transitional EC) and to EC1 (canonical EC) were annotated. **b)** Spatial distribution of the two EC subclusters in a plaque section. Gene expression visualized by **c)** dot plot and **d)** feature plot over a Uniform Manifold Approximation and Projection (UMAP) dimensionality reduction. In the dot plot, the color represents the average expression level of each gene within EC clusters, while the dot size indicates the percentage of cells expressing the gene in each EC cluster. **e)** Gene expression visualized by violin plot. **f)** Heatmap showing the top 10 enriched pathways by p-value for each EC subcluster. **g)** Partition-based graph abstraction (PAGA) graph showing connectivity among the two EC subclusters, with line thickness indicating connection strength. **h)** RNA velocity estimated cellular state transitions between EC subclusters. The stream plot of RNA velocities overlaid on a UMAP plot. **i)** RNA velocity stream mapping to spatial location on a plaque tissue section.

Supplementary Figure 5

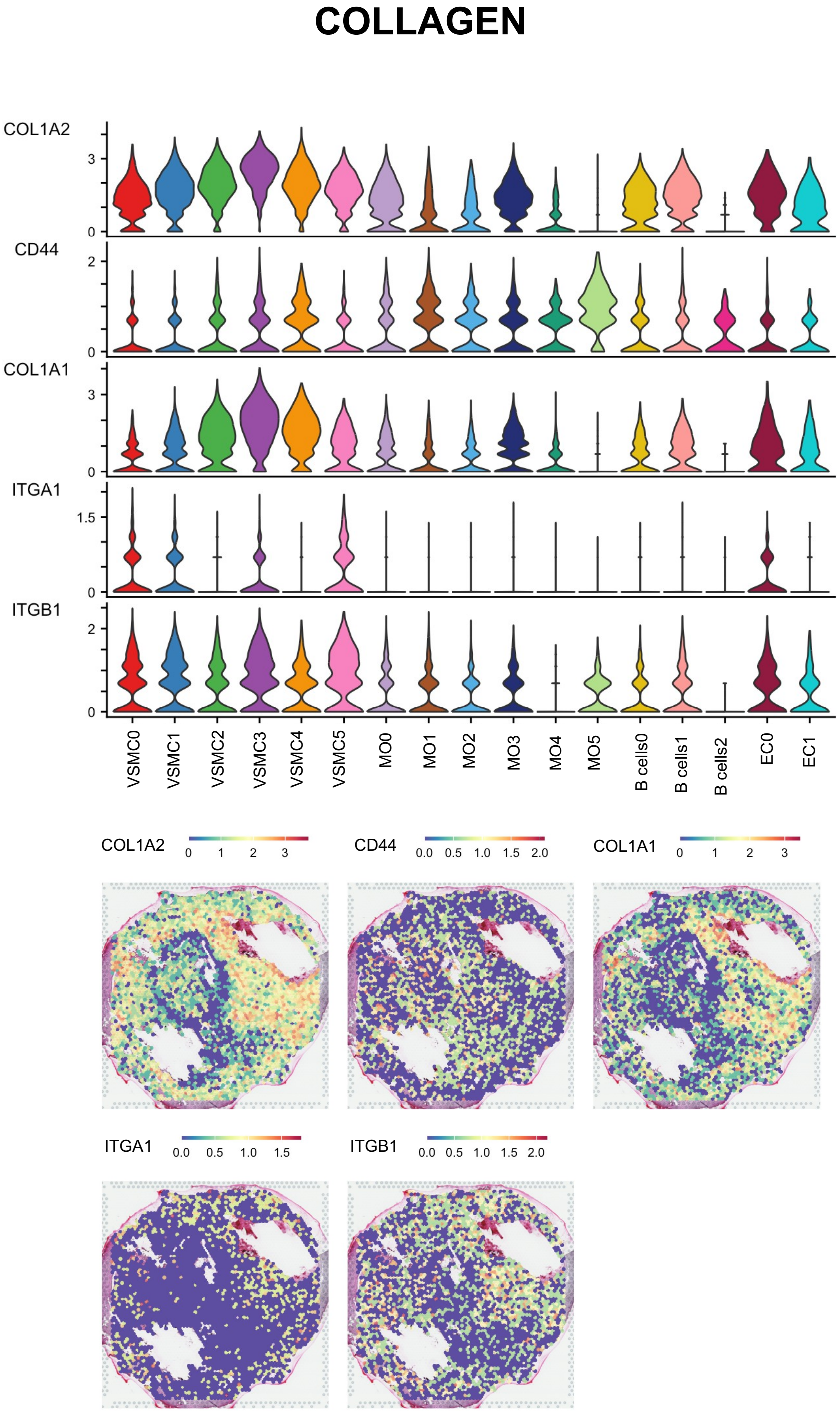

Supplementary Figure 5. Gene expression of ligand-receptors associated with the COLLAGEN signaling pathway in human carotid plaques. Violin plots showing the gene expression of top ligand-receptor pairs driving COLLAGEN signaling in the identified 17 transcriptional states of plaque cells and the spatial distribution of the gene expression visualized on a plaque tissue section. VSMC, vascular smooth muscle cell; MO, macrophage cell; EC, endothelial cell.

Supplementary Figure 6

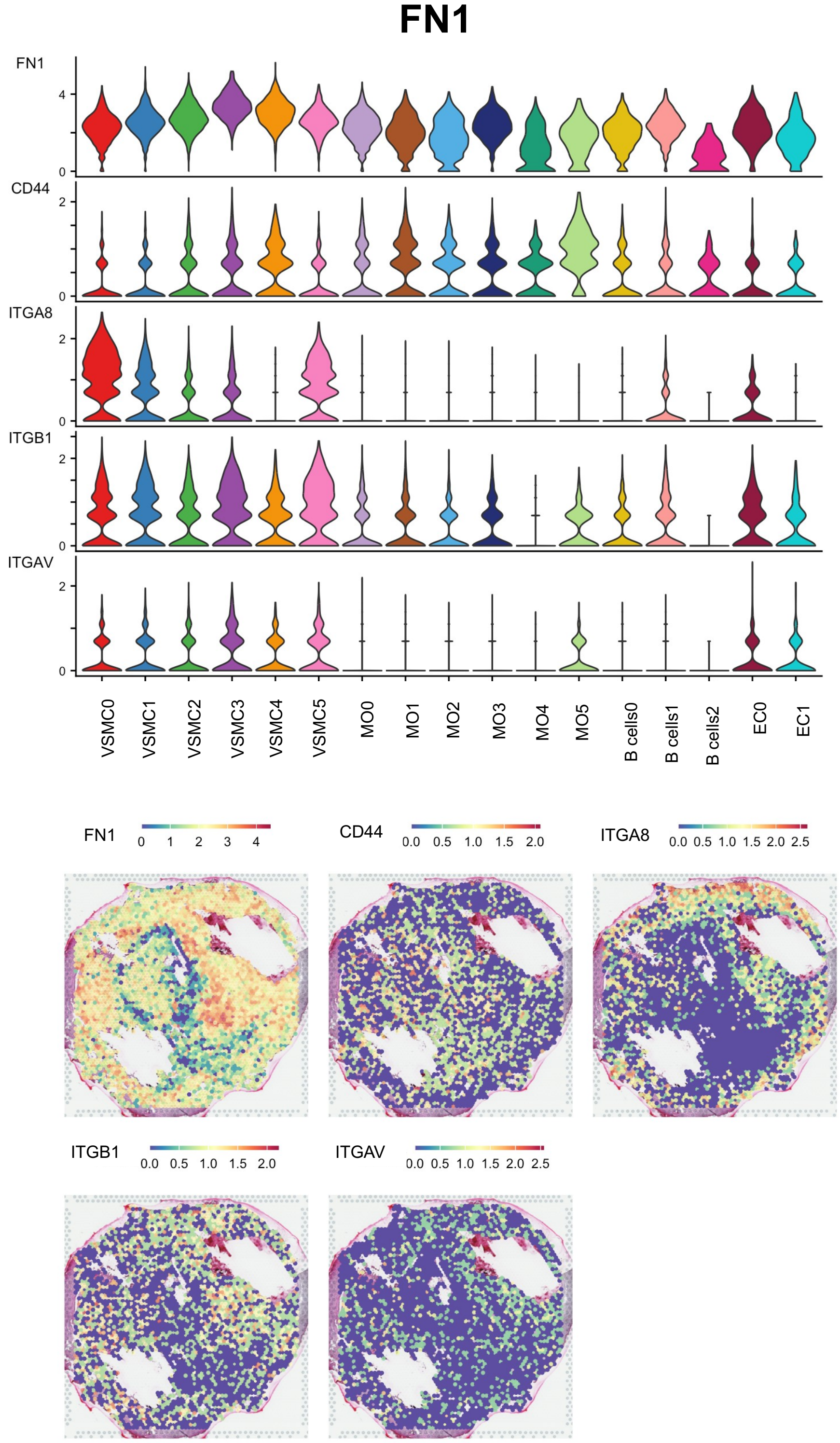

Supplementary Figure 6. Gene expression of ligand-receptors associated with the FN1 signaling pathway in human carotid plaques. Violin plots showing the gene expression of top ligand-receptor pairs driving FN1 signaling in the identified 17 transcriptional states of plaque cells and the spatial distribution of the gene expression visualized on a plaque tissue section. VSMC, vascular smooth muscle cell; MO, macrophage cell; EC, endothelial cell.

Supplementary Figure 7

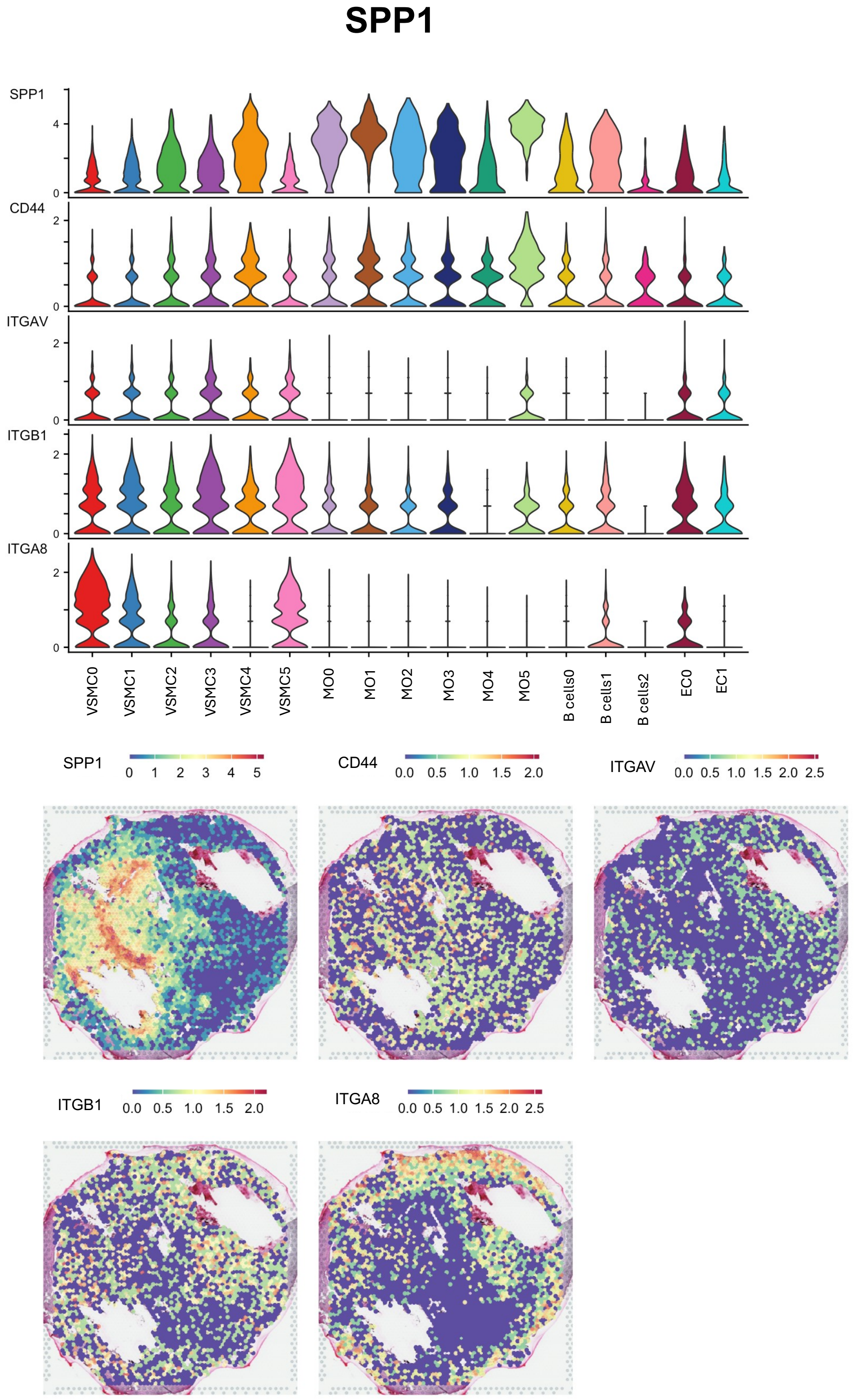

Supplementary Figure 7. Gene expression of ligand-receptors associated with the SPP1 signaling pathway in human carotid plaques. Violin plots showing the gene expression of top ligand-receptor pairs driving SPP1 signaling in the identified 17 transcriptional states of plaque cells and the spatial distribution of the gene expression visualized on a plaque tissue section.. VSMC, vascular smooth muscle cell; MO, macrophage cell; EC, endothelial cell.

Supplementary Figure 8

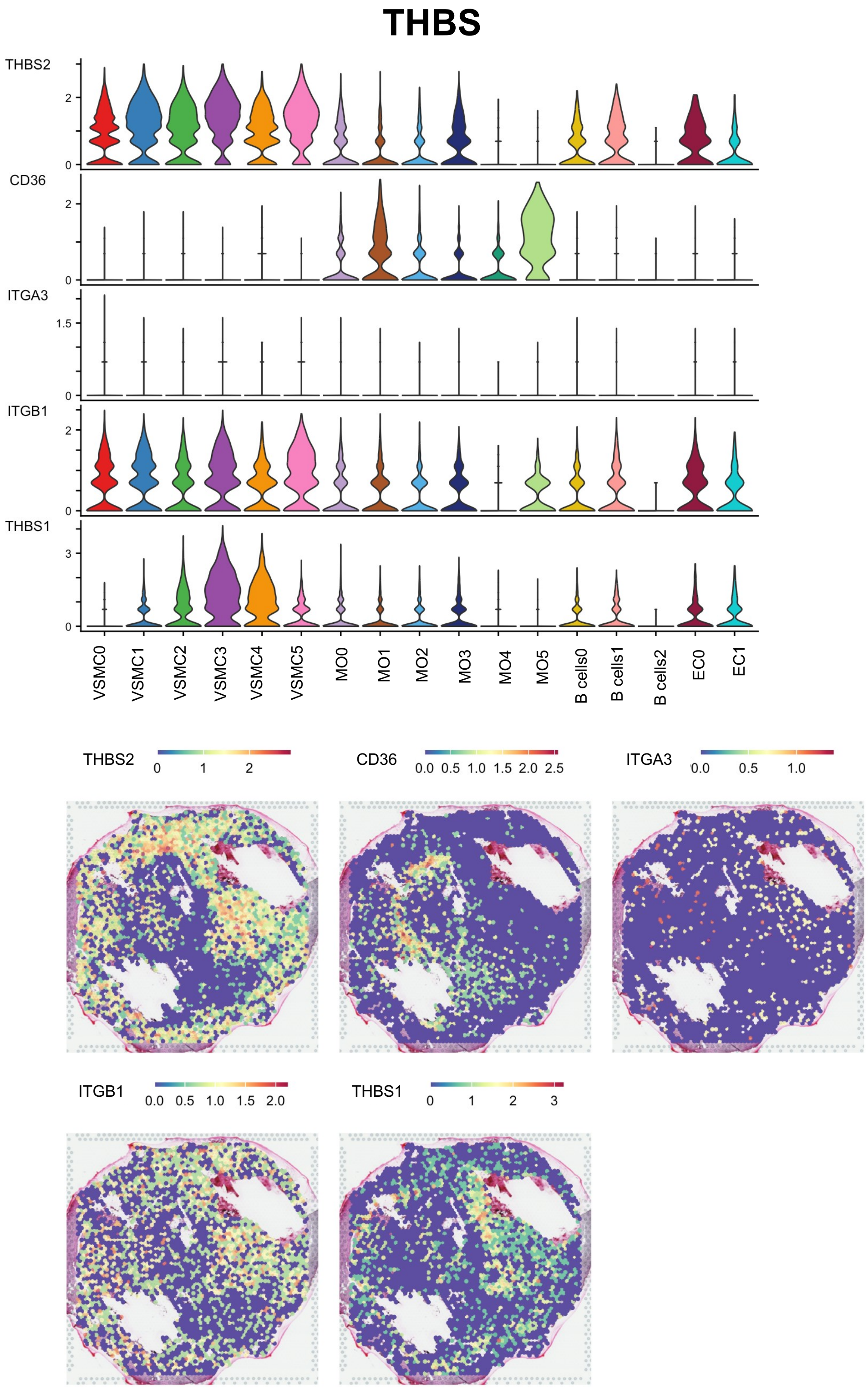

Supplementary Figure 8. Gene expression of ligand-receptors associated with the THBS pathway in human carotid plaques. Violin plots showing the gene expression of top ligand-receptor pairs driving THBS signaling in the identified 17 transcriptional states of plaque cells and the spatial distribution of the gene expression visualized on a plaque tissue section. VSMC, vascular smooth muscle cell; MO, macrophage cell; EC, endothelial cell.

Supplementary Figure 9

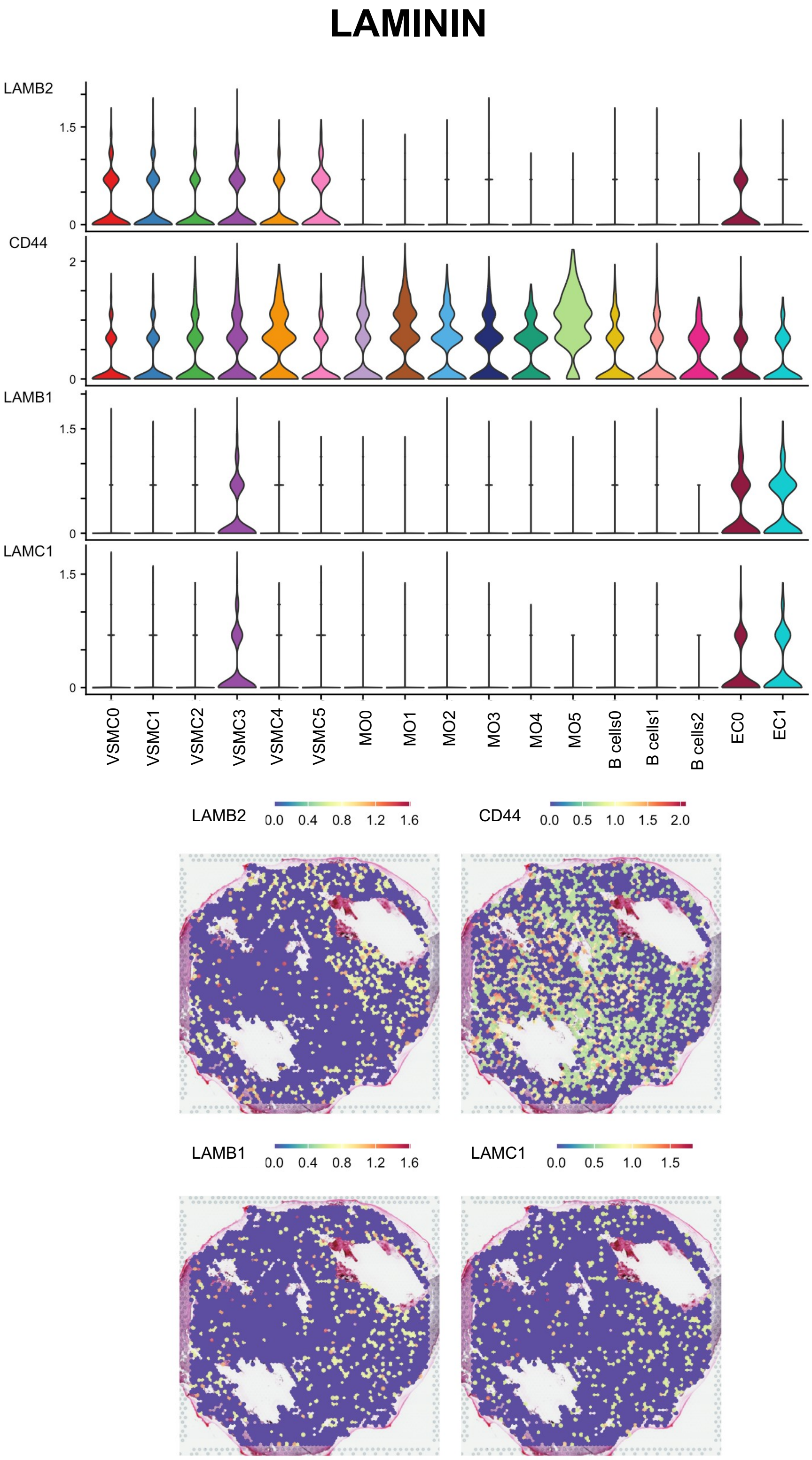

Supplementary Figure 9. Gene expression of ligand-receptors associated with the LAMININ pathway in human carotid plaques. Violin plots showing the gene expression of top ligand-receptor pairs driving LAMININ signaling in the identified 17 transcriptional states of plaque cells and the spatial distribution of the gene expression visualized on a plaque tissue section. VSMC, vascular smooth muscle cell; MO, macrophage; EC, endothelial cell.

Supplementary Figure 10

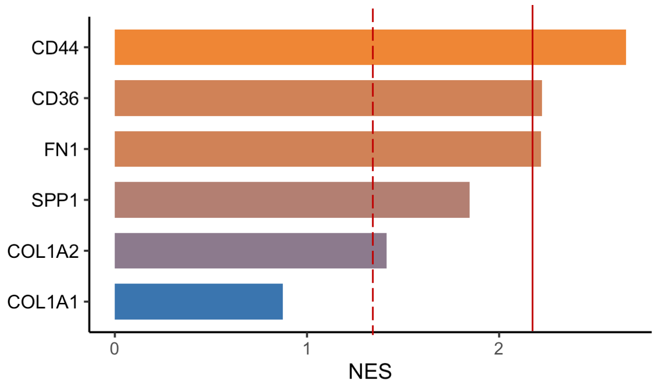

Supplementary Figure 10. *In silico* knock-out of *FN1, SPP1, CD44, CD36,* and *COL1A2* in VSMC3 demonstrated enrichment in elastic fiber formation. Solid line represents BH adjusted p-value of 0.05. Dashed line represents nominal p-value of 0.05. NES: normalized enrichment score

Supplementary Figure 11

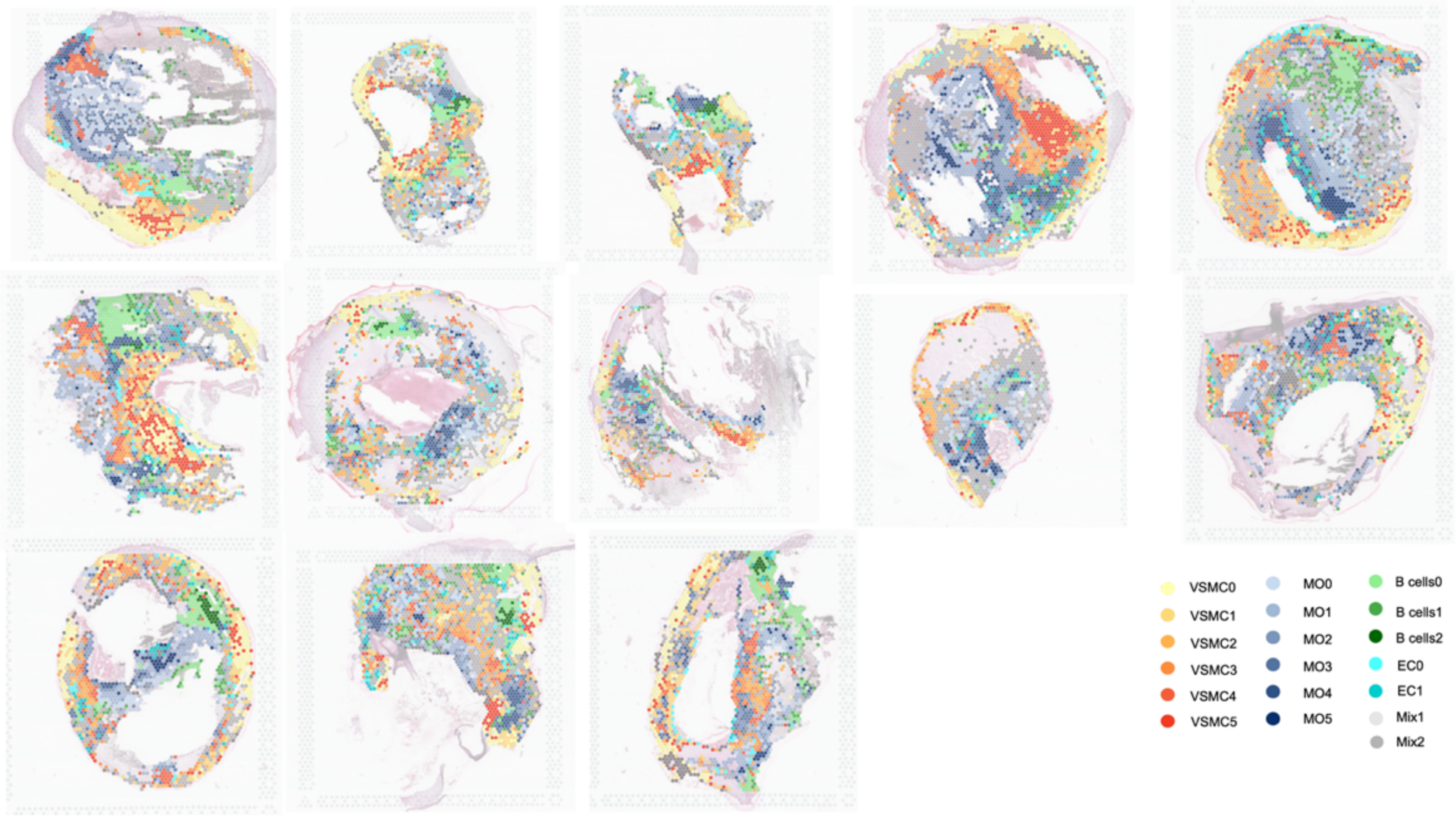

Supplementary Figure 11. Spatial distribution of the 19 plaque cell clusters on 13 plaques. VSMC, vascular smooth muscle cell; MO, macrophage; EC, endothelial cell.

Supplementary Figure 12

a

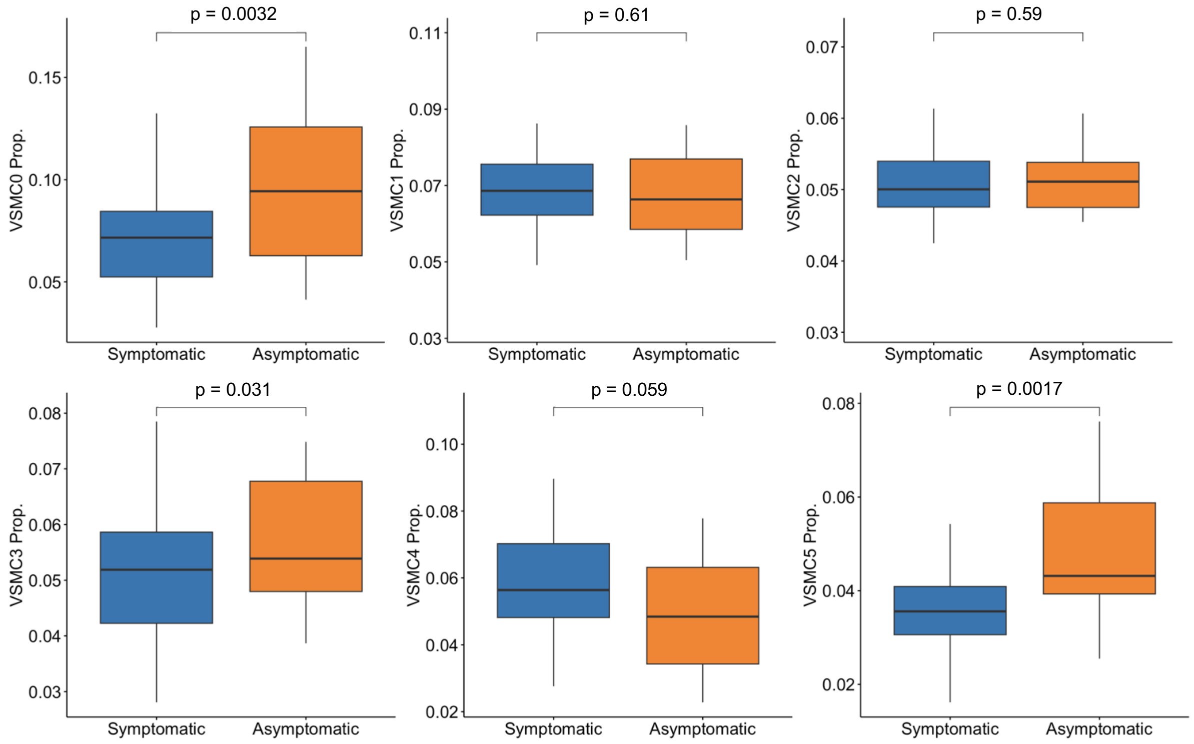

b

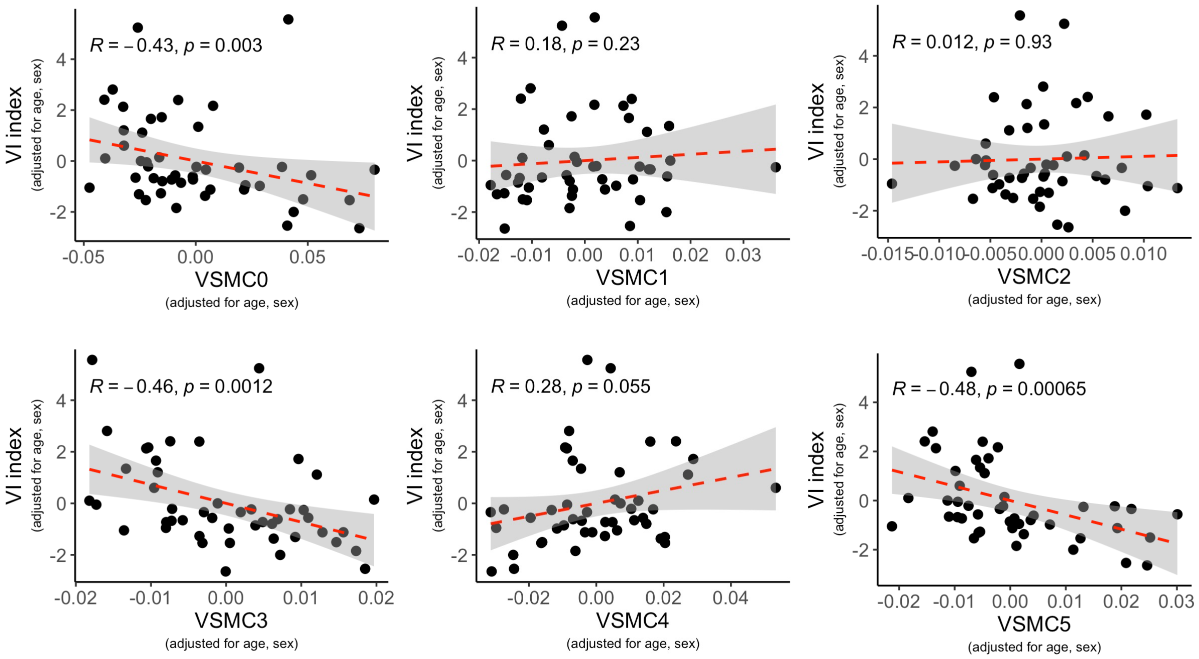

c

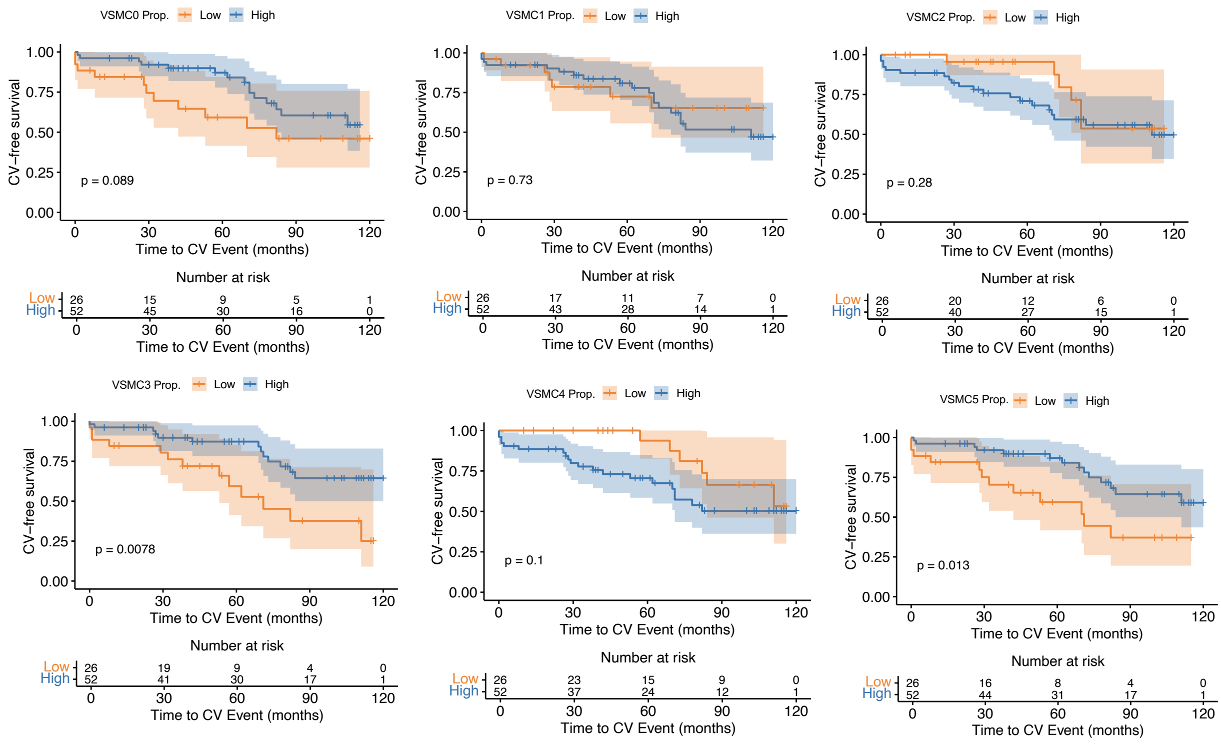

Supplementary Figure 12. a) Cell type deconvolution analyses of 78 human carotid plaques comparing symptomatic (n=51) and asymptomatic (n=27). The proportion (Prop.) of contractile VSMC (VSMC0 and VSMC5) and fibroblast-like VSMC (VSMC3) was greater in asymptomatic plaques compared to symptomatic human carotid plaque. Paired t-test was used. **b)** Scatterplots showing the Spearman correlations, adjusted for age and sex, of the proportions of VSMC phenotypes and the histological vulnerability index. n=47. **c)** Kaplan–Meier curves showing that patients with plaques containing a higher proportion of VSMC3 and VSMC5 (2nd–3rd tertiles) have a significantly lower risk of postoperative cardiovascular events during follow-up compared to those in the lowest tertile (1st tertile). Log-rank test was used. n=78.

Supplementary Figure 13

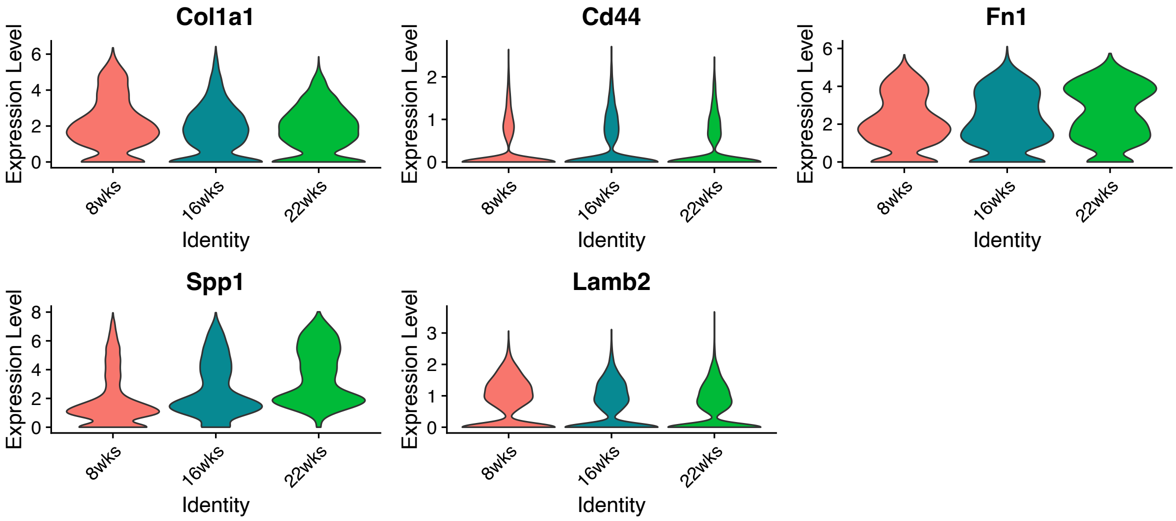

Supplementary Figure 13. mRNA levels of identified key signalling genes in fibrous cap associated-VSMC in ApoE-/- mice subjected to high fat diet for 8 (red), 16 (blue) or 22 weeks (green).

Supplementary Figure 14

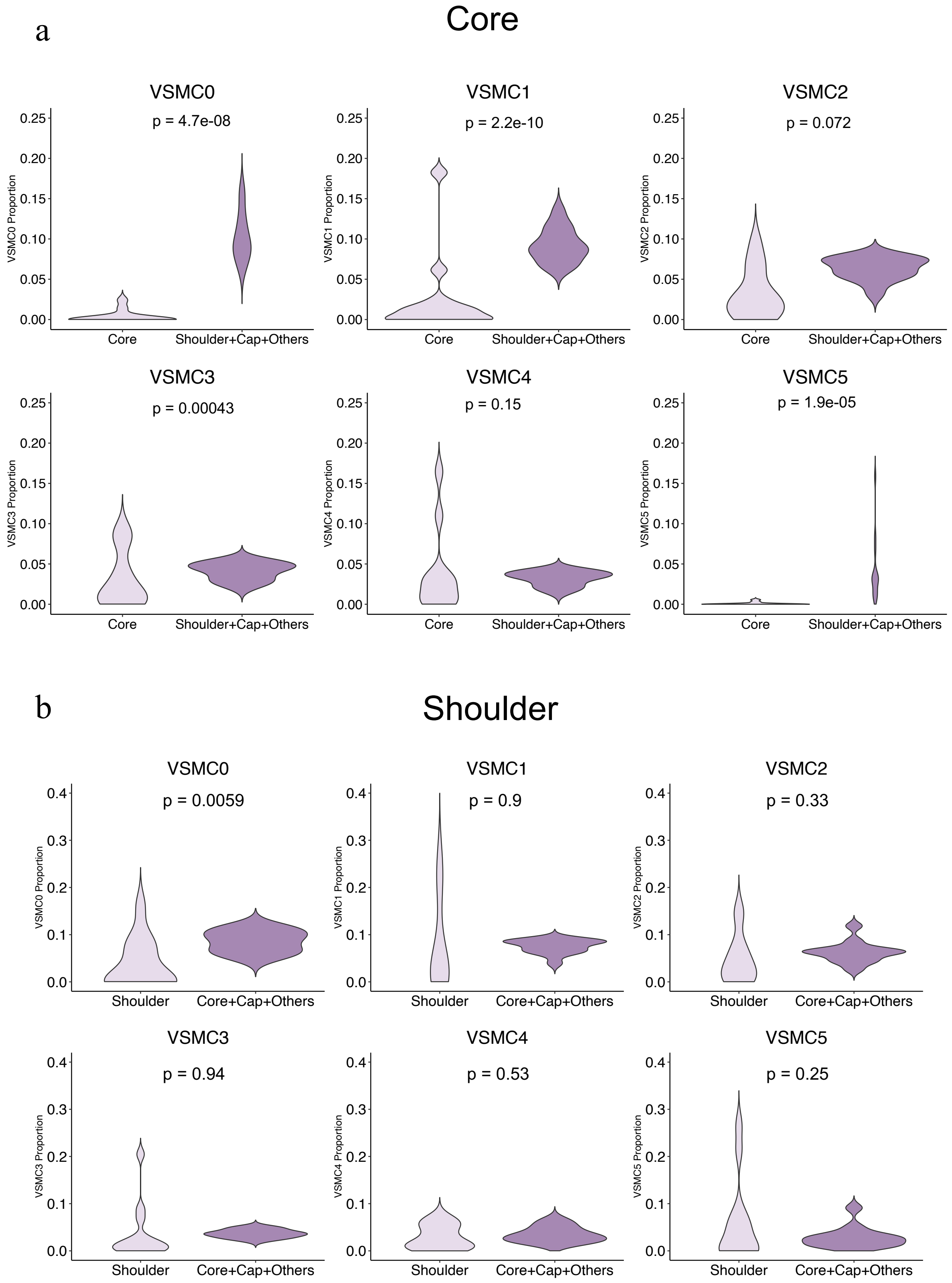

c

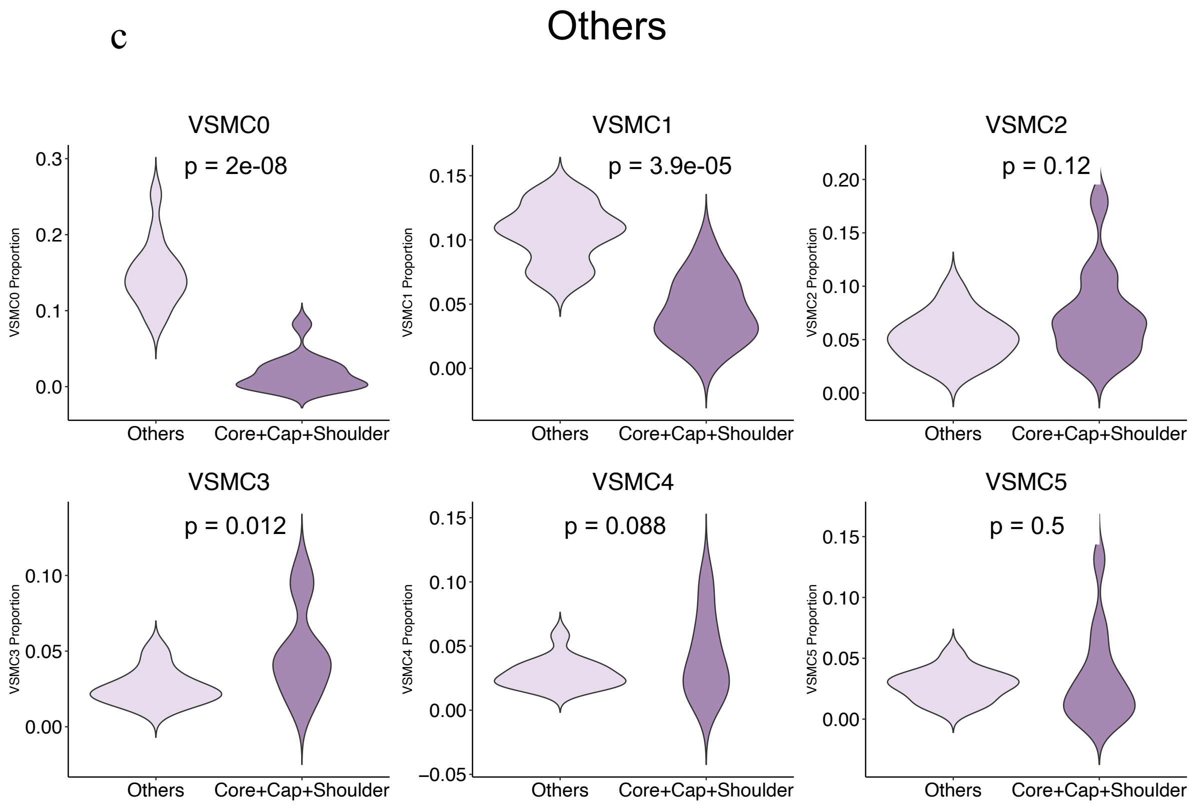

Supplementary Figure 14. Comparisons of the different VSMC spot clusters among the various plaque regions. Spatial transcriptomics analysis of human carotid plaques showing **a)** in the core (compared to the non-core regions (cap, shoulder and other regions)) all VSMC spot clusters were significantly less abundant except macrophage-like VSMC (VSMC2) and foam-cell like VSCMC (VSMC4). **b)** in the shoulder (compared to non-shoulder regions (cap, core and other regions): no significant differences were detected in VSMC spot clusters. **c)** in the other regions (compared to shoulder, core and cap regions): contractile VSMC spot clusters (VSMC0 and 1) were significantly more abundant whereas fibroblast-like VSMC (VSMC3) were significantly less abundant. Student’s t-test was used. n=13 plaques.

Supplementary Figure 15

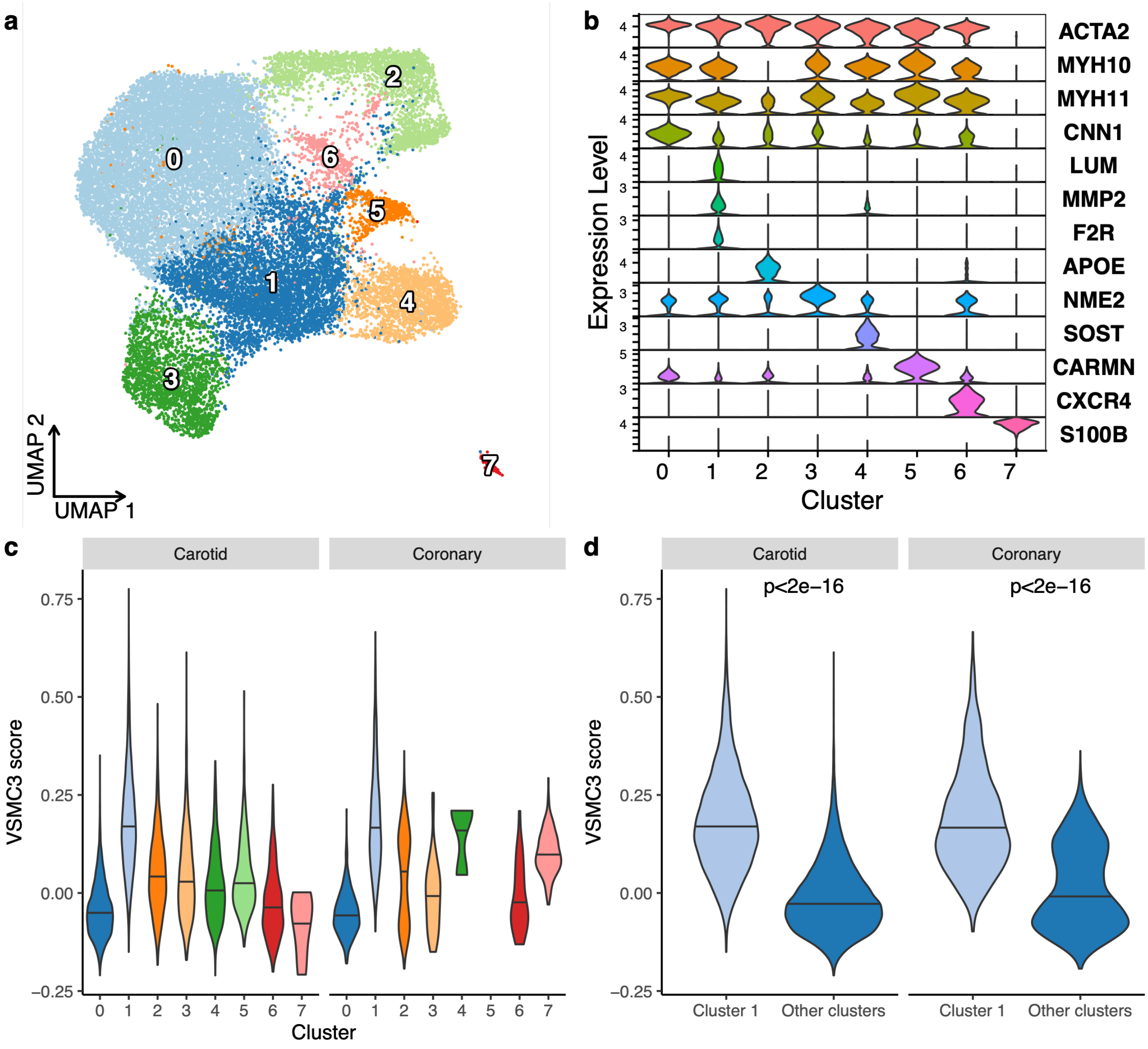

e

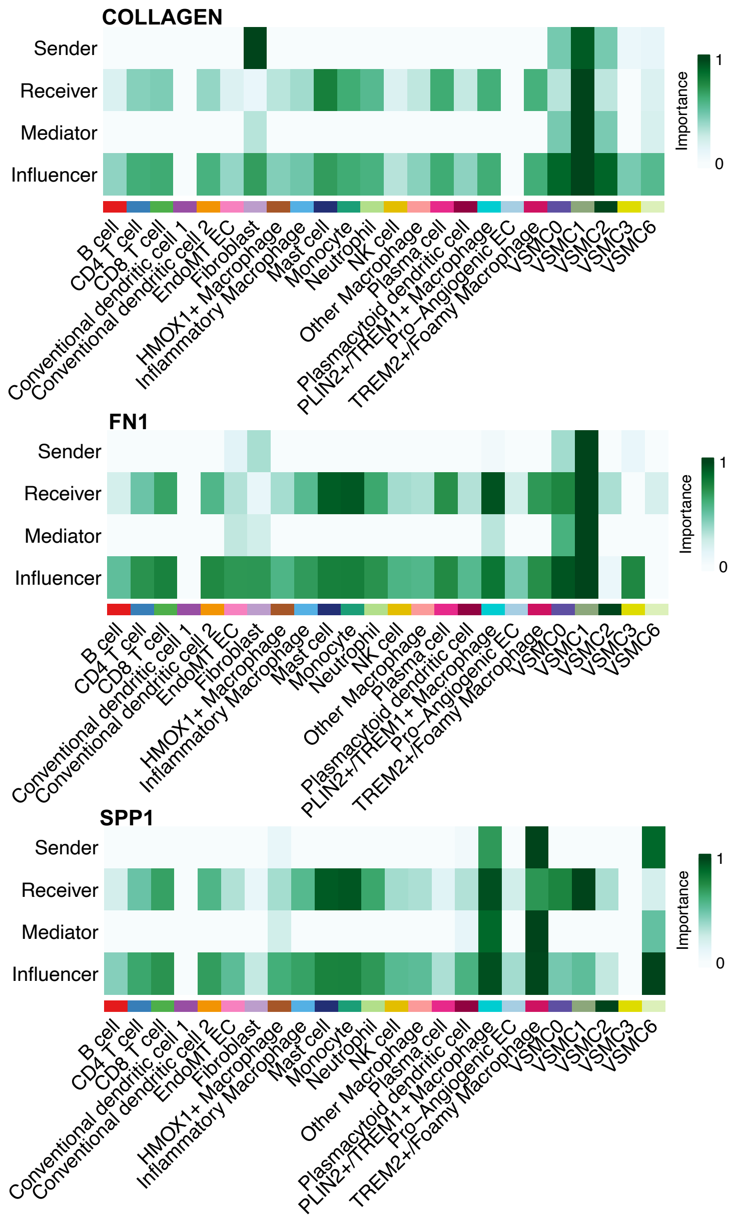

Supplementary Figure 15. Presence of fibroblast-like VSMC (VSMC3) in human atherosclerotic plaques from carotid and coronary arteries. a) Uniform Manifold Approximation and Projection (UMAP) visualization of eight cell clusters identified from carotid (n cells= 20,696) and coronary (n cells= 3,156) plaques, previously annotated as vascular smooth muscle cells (VSMCs) or fibromyocytes. **b)** Violin plots showing the expression of VSMC marker genes (ACTA2, MYH10, MYH11, CNN1) and genes highly expressed in the respective clusters. **c)** Module scores calculate the average expression of genes highly expressed in fibroblast-like VSMCs across cell clusters from carotid and coronary plaques. **d)** Module scores are higher in the cell cluster expressing LUM, MMP2, and F2R compared to other cell clusters. P-values from Wilcox test are reported.

e) In the external validation using a dataset from cells from human coronary plaques (Traeuble et al, PMID: 40931012), we identified signaling pathways in which fibroblast-like VSMCs (VSMC1) exhibited similar roles to VSMC3 observed in our study (n cells=35,859). Minimum number of cells required in each cell cluster for cell-cell communication is 10.

Supplementary Figure 16

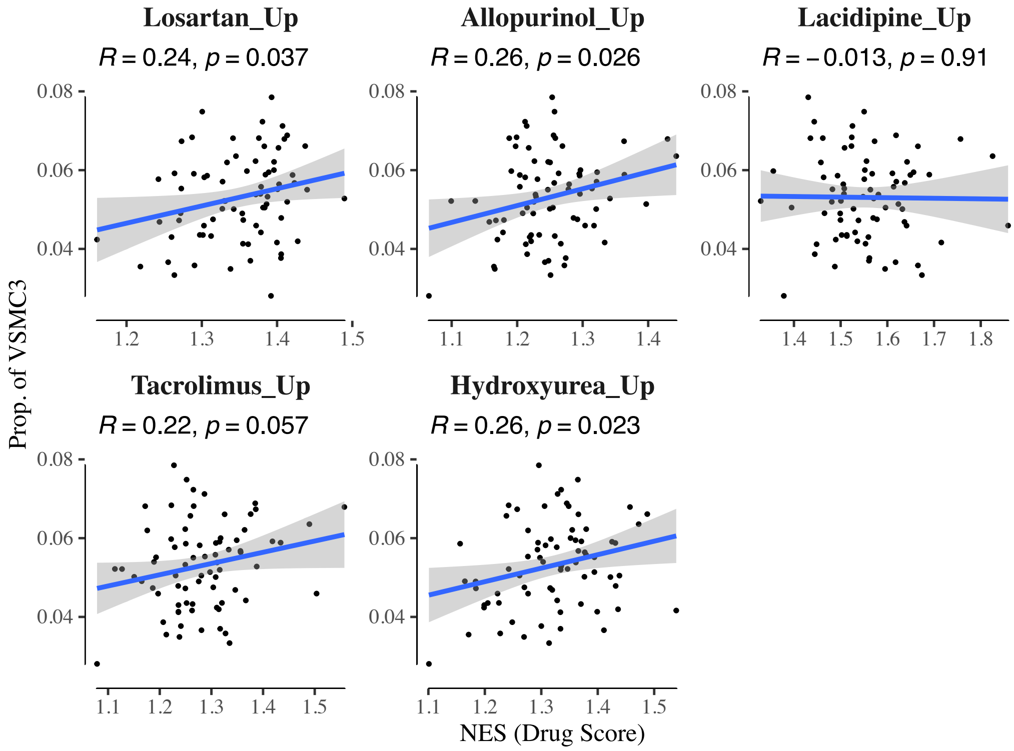

Supplementary Figure 16. Scatterplots showing Pearson's correlations between the proportion of VSMC3 in human carotid plaques and drug scores for the set of genes upregulated in response to losartan, allopurinol, lacidipine, tacrolimus, and hydroxyurea (namely Losartan_Up, Allopurinol_Up, Lacidipine_Up, TacrolimusUp, and Hydroxyurea_Up). NES: normalized enrichment score, Prop.: proportion
