# Supplementary material for: Spatial transcriptomics reveals a key role of fibroblast-like vascular smooth muscle cells in human atherosclerotic cell crosstalk and stability
Source: Eur Heart J. 2026 Feb 13;47(28):3711–32. doi: 10.1093/eurheartj/ehaf1091 (PMC13384730; doi:10.1093/eurheartj/ehaf1091)
Supplement: ehaf1091_Supplementary_Data [file ehaf1091_supplementary_data.zip › SUPPLEMENTARY_METHODS_20260108.pdf]

"SUPPLEMENTARY METHODS"

Spatial transcriptomics reveals a key role of fibroblast-like vascular smooth muscle cells in human atherosclerotic cell crosstalk and stability

Isabel Goncalves, MD, PhD[1,2]; Mengyu Pan[1]; Pratibha Singh, PhD[1]; Wenqi Wang[1]; Jing Zhao, PhD[3]; Lea Dib, PhD[4,5]; Lena Sundius[1]; Ana Persson[1]; Chrysostomi Gialeli, PhD[1]; Panagiotis Fountas[1]; Mihaela Nitulescu[1]; Jan Nilsson, MD, PhD[1]; Stephen Malin, PhD[6]; Claudia Monaco, MD, PhD[4]; Helle F Jørgensen, PhD[3]; Jiangming Sun, PhD[1#]; Andreas Edsfeldt, MD, PhD[1,2,7#]

[#]Shared senior authorship

[1]Cardiovascular Research–Translational Studies, Clinical Science Malmö, Lund University, Jan Waldenströms gata 35, SE-214 28, Malmö, Sweden.
[2]Department of Cardiology, University Hospital of Skåne, Jan Waldenströms gata 15, SE-205 02, Sweden.
[3]Section of Cardiorespiratory Medicine, University of Cambridge, VPD Heart and Lung Research Institute, Papworth Road, Cambridge Biomedical Campus, Cambridge, CB2 0BB, UK
[4]Kennedy Institute of Rheumatology, Nuffield Department of Orthopaedics, Rheumatology and Musculoskeletal Sciences, University of Oxford, Oxford, UK
[5]School of Biological Sciences, University of Reading, Berkshire, UK
[6]Department of Medicine Solna, Karolinska Institute, Stockholm, Sweden
[7]Wallenberg Centre for Molecular Medicine, Lund University, Lund, Sweden.

Short title: Spatial cell communications in human plaque microenvironments

Carotid Plaque Imaging Project

Carotid plaques from the Carotid Plaque Imaging Project (CPIP; Clinical-Trials.gov ID NCT05821894) biobank were used for the present study[1]. Patients were included if they were accepted for carotid endarterectomy due to a carotid plaque with a degree of stenosis >70% (as assessed by Duplex ultrasound) and cerebrovascular symptoms (amaurosis fugax, stroke or transient ischemic attack) or a degree of stenosis >80% without cerebrovascular symptoms. Clinical characteristics of the study cohort are summarized in Table 1. The study has been approved by the Swedish ethical committee (472/2005, 2014/904, 2017/89, 2018/63, 27-2020/3.1, 60/2008, 2012/209). Written consent was provided by all participants and the study follows the declaration of Helsinki. All plaques were instantly snap-frozen in liquid nitrogen upon surgical removal.

Carotid plaque preparation for histology and spatial transcriptomic analysis

Spatial transcriptomics was performed on sections from the most stenotic region of 13 human carotid plaques using the Visium Spatial Gene Expression Slide & Reagent Kit, 16 reactions (Catalog #PN-1000184) following the manufacturer's protocol (CG000239 RevD,10x Genomics, Pleasanton, CA, USA). OCT-embedded 10 μm sections of frozen plaques were placed on the visium spatial slide, fixed with methanol, stained with hematoxylin and eosin (H&E), and imaged by ScanScope Console (Version 8.2, LRI imaging AB, Vista CA, USA).

The tissue was then permeabilized for 12 mins which is an optimal time determined by previous optimization experiments[2]. mRNA bound to capture oligos printed on the slide was then reverse transcribed to cDNA, transferred to tubes, amplified and purified following manufacturer's protocol. The cDNA samples were then quantified using Agilent Bioanalyzer High Sensitivity Kit (Catalog #5067-5592; Agilent technology) and used for library preparation as per

manufacturer's guidelines. The libraries were examined for its quality using a Bioanalyzer High Sensitivity chip (Catalog #5067-5592; Agilent technology) and 2.0pM of library sequenced on NextSeq 500/550 using high Output Kit v2.5 (150 Cycles) with a maximum sequencing depth of 100 million read pairs per sample. The sequencing lengths were performed as recommended: read 1: 28 base pairs (bp); i7 index read: 10 bp; i5 index read: 10 bp; and read 2: 90 bp.

Spatial transcriptomic data processing, integration and clustering

Raw base call files from Illumina NextSeq 500/550 sequencers were demultiplexed into FASTQ reads by Space Ranger (version 1.2.2, 10x Genomics Inc. Pleasanton CA, USA). Space Ranger further mapped the obtained reads to human genome assembly GRCh38 and quantified gene expressions using GENCODE transcriptome, release 32. Gene expression data was aligned with H&E-stained images using Space Ranger. Tissue boundaries and compartments were further manually delineated by two independent clinicians using Loupe Browser (version 7.0.1, 10x Genomics Inc. Pleasanton CA, USA).

Further data processing was conducted using a R package Seurat (version 4.4.0)[3]. Sequencing spots located outside the tissue or within the lumen, as well as those with fewer than 200 detected genes, were filtered out. Mitochondrial genes were also excluded from downstream analyses. After that, 'SCTransform'[4] was implemented for normalization and variance stabilization of gene expression to minimize the impact of technical noise. Principal component analysis (PCA) was then performed using top 3, 000 variable genes per sample where first 50 principal components (PCs) were reported. ElbowPlot illustrating the standard deviations of the first 50 principal components is provided in the supplementary methods figure below.

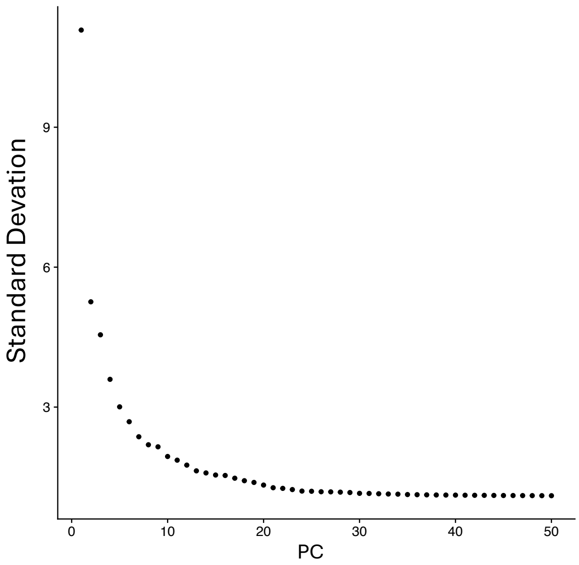

Supplementary methods figure. ElbowPlot illustrating the standard deviations of the first 50 principal components.

To mitigate technical variations among samples, the normalized data from the 13 plaques were integrated, using an anchor-based canonical correlation analysis (CCA) method. Dimensionality reduction was then performed on the integrated data using PCA, followed by Uniform Manifold Approximation and Projection (UMAP). Using the first 30 PCs, a shared nearest neighbor (SNN) graph was constructed to optimize the modularity function and identify spatial clusters via "FindClusters" function with resolution of 0.2.

Cell type deconvolution

Previously published scRNA-seq data[5] from human carotid plaques from three patients were used as a reference to deconvolute the cell types identified in our spatial transcriptomic (ST) analyses of human carotid plaques. This data had been re-analyzed using the PlaqView pipeline[6]. In total, 19 cell clusters were identified, and cell type were previously annotated and aggregated into vascular smooth muscle cells (VSMCs), fibroblasts, endothelial cells (ECs), macrophages (MO), dendritic cells (DC), mast cells, B cells, T cells and NK cells, as previously described[1]. Next, we integrated the scRNA-seq data with our ST dataset using the CCA integration. The aggregated cell types from the scRNA-seq data were then mapped onto the ST data using the

"TransferData" function from the Seurat package[3]. For each spot, prediction scores to be cell types from the scRNA-seq data were reported, ranging from 0 to 1.

Sub-clustering analyses of VSMCs, MO, ECs and B-cells dominating clusters

Sequencing spots within a major cell dominated spatial clusters (VSMCs: cluster 1 and 2 from the integrated data; macrophages: clusters 3 and 4; B-cells: cluster 5; ECs: cluster 6; Fig. 1a-1c) were selected respectively for further clustering analysis. Data normalization, integration and dimensionality reduction were performed using same strategies as previously described for the overall dataset in the present study. Clustering analyses were subsequently conducted using function "FindClusters" from the Seurat package, with the resolution parameter set to 0.5 for VSMCs and macrophages, and 0.2 for ECs and B-cells.

To identify genes that were highly expressed in each subcluster compared to all other subclusters, the Seurat package's "FindAllMarkers" function was utilized employing the Wilcoxon Rank Sum test to compare gene expression between subclusters. Genes with a Benjamini-Hochberg adjusted p-value of less than 0.05 were considered significant. Over-representation analysis was consequently performed on these differentially expressed genes (DEGs) using KEGG database with the Enrichr[7] to identify the enriched pathways.

RNA velocity analysis at spatial resolution

To perform RNA velocity analysis, spliced and unspliced mRNA from each plaque sample were quantified using the Velocyto[8]. RNA velocity was then estimated using the dynamical model in scVelo[9], specifically for sequencing spots from VSMC and MO subclusters. Prior to RNA velocity estimation, the top 3,000 highly variable genes were selected, retaining only those with at least 20 shared counts between unspliced and spliced mRNA. RNA velocities were visualized both in a low-dimensional embedding (i.e., UMAP) and in spatial space as

streamplots, which illustrated the estimated future transcriptional state of cell populations based on RNA velocities.

Cell–cell communications at spatial resolution

Intercellular communications within subclusters of VSMCs, MOs, ECs, and B-cells was analyzed using CellChat[10] (version 2.1.2), specifically adapted for spatially resolved transcriptomics data. Cell-cell communications were limited to a contact range of 100 µm, specifc for 10X visium, an interaction range of 250 µm, reflecting the maximum diffusion range of molecules, and minimum of 10 spatial spots required in each cluster for cell-cell communication. The ligand-receptor interaction database used in this analysis included 2,239 interactions, excluding those involving non-protein signaling pairs[10]. CellChat analysis reported number and weight of significant interaction (strength, $p<0.05$) between clusters. Cell-cell communication at the signaling pathway level summarized the communication probabilities of all ligand-receptor interactions associated with each pathway.

To compare intercellular communication difference within plaque regions (shoulder versus cap and core, cap versus shoulder and core, core versus cap and shoulder), cell-cell communications were performed separately for each region. H&E stained tissue sections were used to define plaque regions as caps (distinct layer of connective tissue that covers the lipid-rich necrotic core towards the lumen of artery), shoulders (region at the edge, transition or margin where the plaque meets the adjacent, less diseased or nearly normal arterial wall), core (central area of the plaque, beneath the fibrous cap, with less cells and altered or no connective tissue structures) or other regions (regions that did not meet the criteria for the cap, shoulder or core regions).

The inferred intercellular communication was then compared between shoulder and non-shoulder (cap and core) regions, cap and non-cap (shoulder and core) regions, core and non-core (cap and shoulder) regions. Interaction strength of signaling pathway between regions was

compared and those signaling pathway significantly increased in cap, core and shoulder regions were reported ($p<0.05$). Similarly, intercellular communication difference between plaques from symptomatic and asymptomatic patients within cap regions were examined.

Plaque histological vulnerability index

Sections from the most stenotic region of the plaque were stained for α-actin, Oil Red O, CD68, glycophorin and Russell–Movat pentachrome to generate a histological vulnerability index by dividing the combined stained areas of CD68, Oil Red O, and glycophorin A by the sum of α-actin and collagen (Movat pentachrome) positive regions, as previously described[11]. The percentage area of each stained plaque component was quantified using Biopix iQ 2.1.8 (Gothenburg, Sweden).

Survival analyses

Information regarding postoperative CV events from 2005 until 2015 (including myocardial infarction, unstable angina, stroke (ipsilateral and contralateral events), transient ischemic attack, amaurosis fugax, vascular interventions (including carotid endarterectomy/stenting, coronary artery bypass grafting/percutaneous coronary artery intervention, and CV death) was collected through the Swedish National Inpatient Health Register and the national cause of death register (2005–2015) as described previously[12].

Bulk RNA sequencing analysis of human carotid plaques and deconvolution of spot cluster compositions

RNA was isolated from the most stenotic region from 78 carotid plaques (51 symptomatic and 27 asymptomatic at baseline) obtained from the CPIP cohort. RNA sequencing was performed using Illumina HiSeq2000 and NextSeq 500 platforms, as previously described[13,14]. The

obtained reads were mapped to transcripts using Salmon[15] based on transcriptome release 27 of GENCODE. Gene counts were summarized using tximport[16] and were normalized between samples using a trimmed mean of M-values (TMM) by edgeR[17], giving gene expressions as log2-transformed counts per million (CPM) after voom transformation. Batch effects of sequencing platforms were adjusted by an empirical Bayes method[18].

Next, plaque spot cluster deconvolution was applied to the bulk RNA-seq data from human carotid plaques, using the obtained 19 spot clusters from the spatial RNA-seq analysis of human carotid plaques as references. Dtangle[19] was used for spot cluster deconvolution to estimate spot cluster composition for each plaque.

The estimated proportions of fibroblast-like VSMCs (VSMC3) in plaques from symptomatic and asymptomatic patients at baseline were compared. To explore associations with future CV events, patients with plaques with a high proportion of VSMC3 (2nd and 3rd tertiles) were compared to patients with a low proportion of VSMC3 (1st tertile) using Kaplan–Meier curves.

In vitro models of fibroblast-like VSMC signalling

Immortalized human coronary artery VSMCs were derived from primary SMCs as previously described[20]. Cells were maintained in GlutaMAX DMEM Gibco™ (Fisher Scientific, Göteborg, Sweden) supplemented with 10% fetal bovine serum (Thermo Fisher Scientific Inc., Waltham, Massachusetts, USA), L-glutamine (Thermo Fisher Scientific Inc., Waltham, Massachusetts, USA), 100 U/mL penicillin and 100 µg/mL streptomycin (Thermo Fisher Scientific Inc., Waltham, Massachusetts, USA), and incubated at 37 °C under 5 % CO2.

To induce phenotypic transition toward a fibroblast-like-state, a sequential stimulation protocol was applied. VSMCs were first treated with 5 ng/mL of transforming growth factor-β1 (TGF-β1) for 24h. Following this, the medium was replaced, and cells were treated with 20 ng/mL platelet-derived growth factor-BB (PDGF-BB) for 48h.

To assess the effect of the CD44 signalling axis on fibroblast-like VSCMs, cells were treated with and without blocking CD44 antibodies (Abs) at a concentration of 2 µg/mL for 24h (ab254530, Abcam), following PDGF-BB treatment.

To assess the effect of pharmacological agents on the phenotypic transition of fibroblast-like VSCMs, candidate drugs (obtained from the drug repurposing analysis) were added to the culture medium during the final 24h of the PDGF-BB treatment phase. Control wells were treated with vehicle (DMSO or the drug) under the same timing.

Total RNA was extracted from VSMCs using RNAeasy Plus Mini kits (Qiagen, Hilden, Germany), and cDNA was synthesized using High-Capacity RNA-to-CDNA™ kit (Applied Biosystems/Thermo Fisher Scientific Inc., Waltham, Massachusetts, USA). Gene expression was analyzed by quantitative real-time PCR on QuantStudio 7 Flex instrument (Applied Biosystems/Thermo Fisher Scientific Inc., Waltham, Massachusetts, USA) using Taqman Fast Advanced master mix and appropriate Taqman probes (Supplementary methods table 1). Relative gene expression was calculated using the $2^{-\Delta Ct}$ method, with hypoxanthine phosphoribosyl transferase 1 serving as endogenous control.

SMCs were lysed in radioimmunoprecipitation assay (RIPA) buffer composed of 150 mM NaCl (Thermo Fisher Scientific Inc., Waltham, Massachusetts, USA), 1.0% Triton X100 (Sigma-Aldrich, Darmstadt, Germany), 0.1% SDS (Sigma-Aldrich, Darmstadt, Germany), 50 mM Tris, pH 8.0 (Sigma-Aldrich, Darmstadt, Germany), supplemented with a Thermo Scientific™ Halt™ Protease Inhibitor Cocktail (100X) (Thermo Fisher Scientific Inc., Waltham, Massachusetts, USA) to 1% final concentration. Total protein concentration was determined using the Pierce™ BCA Protein Assay Kit (Thermo Fisher Scientific Inc., Waltham, Massachusetts, USA), following the manufacturer's protocol.

Cells were fixed for 15 minutes using Kahle's fixative (26% ethanol, 3.7% formaldehyde, 2% glacial acetic acid), followed by PBS washes. Collagen was stained with 0.1% Sirius Red (Direct Red 80 in 1% acetic acid) for 1 hour at room temperature. Excess stain was removed by washing with 0.1M HCl, and collagen-bound dye was subsequently eluted with 0.1M NaOH. Absorbance was measured at 540 nm using Tecan Sunrise Microplate Reader. Collagen content was quantified using a standard curve of rat tail collagen type I (Corning®). Collagen was determined based on a collagen standard curve and normalized to total sample protein content.

All experiments were repeated at least 4-6 times, with bars indicating mean ± SD, and the dots correspond to independent biological repeats for each condition. For direct comparisons between CTRL and TGF-β1/PDGF-BB-treated cells, paired t-tests were used. Statistical analysis was performed using two-way ANOVA followed by Šidák's multiple comparisons test when more than two treatment conditions were compared, such as in experiments including CD44 blocking treatment alongside CTRL and TGF-β1/PDGF-BB-treated cells. Statistical significance was indicated as follows: $p<0.05$ (*), $p<0.01$ (**), $p<0.001$ (***), and $p<0.0001$ (****). Statistical analyses were conducted using RStudio[21] (version 2024.04.0+735) and GraphPad Prism version 8.0.0 for MacOS (GraphPad Software, San Diego, CA, USA).

In silico knockout of key signalling genes

Using the expression matrix from the VSMC3 dataset, *in silico* virtual knock-out of FN1, SPP1, CD44, CD36, COL1A1, and COL1A2 was conducted using the scTenifoldKnk[22]. The enrichment analysis was performed using the ReactomePA database[23]. Normalized enrichment scores and p-values were reported.

Multiplex immunofluorescence staining of VSMC3 markers in human carotid plaques

Human atherosclerotic plaque specimens were formalin-fixed, decalcified, paraffin-embedded, and sectioned. Multiplex immunofluorescence staining was performed using a sequential protocol based on the Phenoptics workflow (Akoya Biosciences). The antibodies used included: anti-Cell Migration-Inducing Hyaluronidase 1 (CEMIP, E8Y4R, Cell Signaling Technology; 4 µg/mL), anti-collagen type I (COLI, ab34710, Abcam; 6 µg/mL), anti-fibronectin 1 (FN1, ab2413, Abcam; 0.05 µg/mL), and anti-αSMA (M0851, Dako; 0.08 µg/mL). Signal detection was achieved using tyramide signal amplification with Opal fluorophores at 1:100 dilution: Opal 480 (CEMIP), Opal 570 (COL I), Opal 780 (FN1), and Opal 690 (αSMA). Cell nuclei were counterstained with DAPI. Slides were deparaffinized in xylene, rehydrated through ethanol gradients, and subjected to heat-induced epitope retrieval using citrate buffer (pH 6). Each staining round consisted of incubation with the primary antibody, followed by horseradish peroxidase-linked secondary detection and fluorophore amplification. Between rounds, bound antibodies were removed using a stripping buffer containing β-mercaptoethanol at 50 °C for 30 minutes, preserving the fluorescent signal. To enable spectral unmixing and autofluorescence correction, single-marker controls, a DAPI-only control, and an unstained section were used to generate a reference spectral library. Imaging was performed with the PhenoImager HT (Akoya Biosciences), and image analysis was carried out using inForm software[24] and QuPath[25].

Proximity Ligation Assay (PLA)

PLA was performed on FFPE tissue sections using the Duolink® in situ red starter kit (Sigma-Aldrich, DUO92101) following the manufacturer's protocol. After deparaffinization and antigen retrieval, sections were blocked and incubated overnight at 4 °C with mouse anti-CD44 (ab254530, Abcam; 1 µg/mL) and rabbit anti-collagen type I (COLI, ab34710, Abcam; 6 µg/mL) primary antibodies. Species-specific PLA probes (PLUS and MINUS) were applied, followed by ligation and rolling circle amplification at 37 °C. Fluorescent signals were

visualized using a fluorescence microscope, and each PLA signal appeared as a discrete red punctum indicating molecular proximity (<40 nm). Fluorescent PLA signals were visualized as distinct red puncta using the Olympus SlideView VS200 scanner (Evident Scientific, Waltham, USA). Images were acquired as maximum intensity projections across the full thickness of each tissue section to ensure detection of all focal-plane-localized PLA signals. PLA puncta were identified based on morphological criteria: small area and high circularity, which distinguish true signals from autofluorescence and nonspecific staining that typically present with larger, irregular shapes and low circularity. Detected PLA signals were overlaid on the original raw images as white crosses for visual verification.

Genome-wide gene-based association and gene-set analyses for genes highly expressed in fibroblast-like VSMC

To determine whether fibroblast-like VSMCs (VSMC3) were implicated in large artery stroke (LAS), coronary artery disease (CAD) or myocardial infarction (MI), genome-wide gene-based associations and gene-set analyses were conducted using MAGMA[26]. For each gene that was highly expressed in the VSMC3 cluster, MAGMA aggregated the cumulative effects of single nucleotide polymorphisms (SNPs) located within 35 kb upstream and 10 kb downstream of each gene, utilizing summary statistics from genome-wide association studies (GWAS) of LAS[27] (n=1,241,785, n cases=6,977), CAD[28] (n=1,165,690, n cases=181,522) and MI[29] (n=1,110,717, n cases=61,505) where the majority of the study participants were European. A p-value was reported for each gene whereas genome-wide significance was defined as 0.05 divided by number of genes examined, i.e., 2.58×10^{-6}. Next, gene-set analyses were implemented by MAGMA to evaluate whether associations between all the genes highly expressed in VSMC3 with LAS, CAD and MI are greater than in other genes.

A reference data set (1000 Genomes European panel) was used to account for linkage disequilibrium between SNPs. Prior to analysis, GWAS summary data was cleaned to make sure only biallelic autosomal SNPs with minor allele frequency greater than 0.05 was kept.

Validation of the mechanism of cap formation in Apoe$^{-/-}$ mice model showing the similarity between human fibroblast-like VSMC (VSMC3) and Apoe$^{-/-}$ fibrous cap associated VSMC

Publicly available single cell RNA-seq profiles of lineage-labelled VSMCs from healthy *Apoe*$^{+/+}$ mice (available in the Gene Expression Omnibus (GEO) database under accession code GSE274572) and high-fat-diet feeding *Apoe*$^{-/-}$ mice (available in the GEO database under accession code GSE155513) were used to test for presence of an equivalent fibroblast-like VSMC phenotype in mice[30]. Briefly, data was integrated and clustered using Seurat, followed by identification of main VSMC states based on expression of marker genes, as described[30]. Violin plots were made for genes highly expressed in VSMC3 (identified in this study, converted to mouse IDs using biomart (version 2.62.1) with the Ensembl 107 assembly) to compare gene expressions across cells from mouse plaques. An expression score (UCell signature score) was computed using UCell (version 2.10.1)[31] for all the transcriptional states of plaque cells obtained from the *Apoe*$^{-/-}$ model, using genes highly expressed in VSMC3 as a signature. Such scores were compared to identify mouse cells with phenotypic similarity to human fibroblast-like VSMCs.

Validation of fibroblast-like VSMC (VSMC3) in atherosclerotic plaques from different arterial territories.

To investigate if the fibroblast-like VSMC (VSMC3) reflects a transcriptional state not only present in carotid plaques but also in other arterial territories, an integrated single cell dataset of plaque cells from human carotid, coronary and femoral arteries was used[32]. Based on this

pre-processed data, cells that were previously annotated as smooth muscle cells and fibromyocytes were further integrated by fastMNN method[33] using top 2,000 highly variable genes. In total, 23,852 cells from human carotid (n cells=20,692) and coronary (n cells=3,156) plaques were used for further analyses. By sub-clustering on the integrated dataset using the Seurat[3], 8 subclusters were identified at a resolution of 0.2. Cell phenotypes were annotated using genes that were highly expressed in each subcluster compared to all other subclusters.

Using genes that were highly expressed in fibroblast-like VSMCs (VSMC3), module scores were calculated by the Seurat[3] for each cell cluster and compared across the subclusters and origins (carotid or coronary plaques). P-value from Kruskal-Wallis test was reported.

The cell communication analyses were also conducted by using the VSMC subclusters annotation, together with other human coronary plaque cells in the public dataset (n=35,859)[32].

Candidate drug screening

Overlapping genes highly expressed in both human fibroblast-like VSMCs (VSMC3) and the phenotypically similar fibrous cap-associated VSMCs from *Apoe*$^{-/-}$ mice were used to explore LINCS L1000 chemical perturbation consensus signatures[34] through Enrichr[35] aiming to identify compounds that affect these marker genes in human cell lines. To evaluate potential toxicity or beneficial transcriptional effects of these selected compounds *in vivo*, the DrugMatrix toxicogenomic database[36] was used through Enrichr, focusing on gene expression profiles following high-dose drug exposures in rat. For both analyses, Enrichr reported odds ratios, nominal p-values, and Benjamini-Hochberg (BH)-adjusted p-values for the enriched compounds. A BH-adjusted p-value<0.05 was considered statistically significant. Compounds that significantly upregulated VSMC3-associated genes in both human cell lines and rat tissues were prioritized as candidate drugs for future possible repurposing in the context of atherosclerosis.

In-silico drug scores

To construct the drug score, a single-sample gene set enrichment analysis was performed on bulk RNA sequencing data from human carotid plaques (n=78). Briefly, log_2-transformed gene expression values for each sample were ranked, and a normalized enrichment score (named drug score thereafter) was calculated for each gene set from the LINCS L1000 chemical perturbation consensus signatures dataset[34]. This dataset contains consensus gene expression signatures derived from chemical perturbations measured using the L1000 platform, summarizing cellular transcriptional responses to various drugs and small molecules by integrating multiple experiments into robust, representative signatures. For simplicity, only gene sets corresponding to genes upregulated in response to specific chemical perturbations were used. Higher drug scores indicate stronger transcriptional similarity to the perturbation signature, reflecting a higher inferred drug activity or exposure.

Supplementary methods table 1. Primers

Gene	Assay ID	Species	Target Description
FN1	Hs01549976_m1	Human	Fibronectin 1
KLF4	Hs00358836_m1	Human	Kruppel Like Factor 4
CD44	Hs01075864_m1	Human	cluster of differentiation 44
THBS2	Hs01568063_m1	Human	Thrombospondin 2
COL1A2	Hs01028956_m1	Human	Collagen Type I Alpha 2 Chain
COL1A1	Hs00164004_m1	Human	Collagen Type I Alpha 1 Chain
COL5A2	Hs00893878_m1	Human	Collagen Type V Alpha 2 Chain
COL3A1	Hs00943809_m1	Human	Collagen Type III Alpha 1 Chain
HPRT1	Hs02800685_m1	Human	Hypoxanthine Phosphoribosyltransferase 1

References

1. Singh P, Sun J, Cavalera M, Al-Sharify D, Matthes F, Barghouth M, et al. Dysregulation of MMP2-dependent TGF-ss2 activation impairs fibrous cap formation in type 2 diabetes-associated atherosclerosis. *Nat Commun* 2024;**15**:10464. doi: https://doi.org/10.1038/s41467-024-50753-8
2. Sun J, Singh P, Shami A, Kluza E, Pan M, Djordjevic D, et al. Spatial Transcriptional Mapping Reveals Site-Specific Pathways Underlying Human Atherosclerotic Plaque Rupture. *J Am Coll Cardiol* 2023;**81**:2213-2227. doi: https://doi.org/10.1016/j.jacc.2023.04.008
3. Hao Y, Hao S, Andersen-Nissen E, Mauck WM, 3rd, Zheng S, Butler A, et al. Integrated analysis of multimodal single-cell data. *Cell* 2021;**184**:3573-3587 e3529. doi: https://doi.org/10.1016/j.cell.2021.04.048
4. Hafemeister C, Satija R. Normalization and variance stabilization of single-cell RNA-seq data using regularized negative binomial regression. *Genome Biol* 2019;**20**:296. doi: https://doi.org/10.1186/s13059-019-1874-1
5. Alsaigh T, Evans D, Frankel D, Torkamani A. Decoding the transcriptome of calcified atherosclerotic plaque at single-cell resolution. *Commun Biol* 2022;**5**:1084. doi: https://doi.org/10.1038/s42003-022-04056-7
6. Ma WF, Turner AW, Gancayco C, Wong D, Song Y, Mosquera JV, et al. PlaqView 2.0: A comprehensive web portal for cardiovascular single-cell genomics. *Front Cardiovasc Med* 2022;**9**:969421. doi: https://doi.org/10.3389/fcvm.2022.969421
7. Chen EY, Tan CM, Kou Y, Duan Q, Wang Z, Meirelles GV, et al. Enrichr: interactive and collaborative HTML5 gene list enrichment analysis tool. *BMC Bioinformatics* 2013;**14**:128. doi: https://doi.org/10.1186/1471-2105-14-128
8. La Manno G, Soldatov R, Zeisel A, Braun E, Hochgerner H, Petukhov V, et al. RNA velocity of single cells. *Nature* 2018;**560**:494-498. doi: https://doi.org/10.1038/s41586-018-0414-6
9. Bergen V, Lange M, Peidli S, Wolf FA, Theis FJ. Generalizing RNA velocity to transient cell states through dynamical modeling. *Nat Biotechnol* 2020;**38**:1408-1414. doi: https://doi.org/10.1038/s41587-020-0591-3
10. Jin S, Plikus MV, Nie Q. CellChat for systematic analysis of cell-cell communication from single-cell transcriptomics. *Nat Protoc* 2024. doi: https://doi.org/10.1038/s41596-024-01045-4
11. Goncalves I, Sun J, Tengryd C, Nitulescu M, Persson AF, Nilsson J, et al. Plaque Vulnerability Index Predicts Cardiovascular Events: A Histological Study of an Endarterectomy Cohort. *J Am Heart Assoc* 2021;**10**:e021038. doi: https://doi.org/10.1161/JAHA.120.021038
12. Tomas L, Edsfeldt A, Mollet IG, Perisic Matic L, Prehn C, Adamski J, et al. Altered metabolism distinguishes high-risk from stable carotid atherosclerotic plaques. *Eur Heart J* 2018;**39**:2301-2310. doi: https://doi.org/10.1093/eurheartj/ehy124
13. Edsfeldt A, Dunér P, Ståhlman M, Mollet IG, Asciutto G, Grufman H, et al. Sphingolipids Contribute to Human Atherosclerotic Plaque Inflammation. *Arteriosclerosis, Thrombosis, and Vascular Biology* 2016;**36**:1132-1140. doi: https://doi.org/doi:10.1161/ATVBAHA.116.305675
14. Gonçalves I, Singh P, Tengryd C, Cavalera M, Yao Mattisson I, Nitulescu M, et al. sTRAIL-R2 (Soluble TNF [Tumor Necrosis Factor]-Related Apoptosis-Inducing

Ligand Receptor 2) a Marker of Plaque Cell Apoptosis and Cardiovascular Events. *Stroke* 2019;**50**:1989-1996. doi: https://doi.org/10.1161/strokeaha.119.024379
15. Patro R, Duggal G, Love MI, Irizarry RA, Kingsford C. Salmon provides fast and bias-aware quantification of transcript expression. *Nat Methods* 2017;**14**:417-419. doi: https://doi.org/10.1038/nmeth.4197
16. Soneson C, Love MI, Robinson MD. Differential analyses for RNA-seq: transcript-level estimates improve gene-level inferences. *F1000Res* 2015;**4**:1521. doi: https://doi.org/10.12688/f1000research.7563.2
17. Robinson MD, McCarthy DJ, Smyth GK. edgeR: a Bioconductor package for differential expression analysis of digital gene expression data. *Bioinformatics* 2010;**26**:139-140. doi: https://doi.org/10.1093/bioinformatics/btp616
18. Johnson WE, Li C, Rabinovic A. Adjusting batch effects in microarray expression data using empirical Bayes methods. *Biostatistics* 2007;**8**:118-127. doi: https://doi.org/10.1093/biostatistics/kxj037
19. Hunt GJ, Freytag S, Bahlo M, Gagnon-Bartsch JA. dtangle: accurate and robust cell type deconvolution. *Bioinformatics* 2018;**35**:2093-2099. doi: https://doi.org/10.1093/bioinformatics/bty926
20. Heuschkel MA, Babler A, Heyn J, van der Vorst EPC, Steenman M, Gesper M, et al. Distinct role of mitochondrial function and protein kinase C in intimal and medial calcification in vitro. *Front Cardiovasc Med* 2022;**9**:959457. doi: https://doi.org/10.3389/fcvm.2022.959457
21. Posit team. RStudio: Integrated Development Environment for R. Posit Software, PBC, Boston, MA. URL http://www.posit.co/. 2025.
22. Osorio D, Zhong Y, Li G, Xu Q, Yang Y, Tian Y, et al. scTenifoldKnk: An efficient virtual knockout tool for gene function predictions via single-cell gene regulatory network perturbation. *Patterns* 2022;**3**:100434. doi: https://doi.org/https://doi.org/10.1016/j.patter.2022.100434
23. G Y, Q H. ReactomePA: an R/Bioconductor package for reactome pathway analysis and visualization. *Molecular BioSystems* 2016; **12(12), 477-479**. doi: https://doi.org/doi:10.1039/C5MB00663E
24. Kramer AS, Latham B, Diepeveen LA, Mou L, Laurent GJ, Elsegood C, et al. InForm software: a semi-automated research tool to identify presumptive human hepatic progenitor cells, and other histological features of pathological significance. *Sci Rep* 2018;**8**:3418. doi: https://doi.org/10.1038/s41598-018-21757-4
25. Bankhead P, Loughrey MB, Fernández JA, Dombrowski Y, McArt DG, Dunne PD, et al. QuPath: Open source software for digital pathology image analysis. *Scientific Reports* 2017;**7**:16878. doi: https://doi.org/10.1038/s41598-017-17204-5
26. de Leeuw CA, Mooij JM, Heskes T, Posthuma D. MAGMA: Generalized Gene-Set Analysis of GWAS Data. *PLOS Computational Biology* 2015;**11**:e1004219. doi: https://doi.org/10.1371/journal.pcbi.1004219
27. Mishra A, Malik R, Hachiya T, Jürgenson T, Namba S, Posner DC, et al. Stroke genetics informs drug discovery and risk prediction across ancestries. *Nature* 2022;**611**:115-123. doi: https://doi.org/10.1038/s41586-022-05165-3
28. Aragam KG, Jiang T, Goel A, Kanoni S, Wolford BN, Atri DS, et al. Discovery and systematic characterization of risk variants and genes for coronary artery disease in over a million participants. *Nat Genet* 2022;**54**:1803-1815. doi: https://doi.org/10.1038/s41588-022-01233-6

29. Hartiala JA, Han Y, Jia Q, Hilser JR, Huang P, Gukasyan J, et al. Genome-wide analysis identifies novel susceptibility loci for myocardial infarction. *Eur Heart J* 2021;**42**:919-933. doi: https://doi.org/10.1093/eurheartj/ehaa1040
30. Taylor JCK, Worssam MD, Oc S, Lambert J, Mahbubani KT, Foote K, et al. Delineation of a thrombin receptor-stimulated vascular smooth muscle cell transition generating cells in the plaque-stabilizing fibrous cap. *Cardiovascular Research* 2025;**121**:1359-1372. doi: https://doi.org/10.1093/cvr/cvaf112
31. Andreatta M, Carmona SJ. UCell: Robust and scalable single-cell gene signature scoring. *Comput Struct Biotechnol J* 2021;**19**:3796-3798. doi: https://doi.org/10.1016/j.csbj.2021.06.043
32. Traeuble K, Munz M, Pauli J, Sachs N, Vafadarnejad E, Carrillo-Roa T, et al. Integrated single-cell atlas of human atherosclerotic plaques. *Nat Commun* 2025;**16**:8255. doi: https://doi.org/10.1038/s41467-025-63202-x
33. Haghverdi L, Lun ATL, Morgan MD, Marioni JC. Batch effects in single-cell RNA-sequencing data are corrected by matching mutual nearest neighbors. *Nature Biotechnology* 2018;**36**:421-427. doi: https://doi.org/10.1038/nbt.4091
34. Subramanian A, Narayan R, Corsello SM, Peck DD, Natoli TE, Lu X, et al. A Next Generation Connectivity Map: L1000 Platform and the First 1,000,000 Profiles. *Cell* 2017;**171**:1437-1452 e1417. doi: https://doi.org/10.1016/j.cell.2017.10.049
35. Kuleshov MV, Jones MR, Rouillard AD, Fernandez NF, Duan Q, Wang Z, et al. Enrichr: a comprehensive gene set enrichment analysis web server 2016 update. *Nucleic Acids Res* 2016;**44**:W90-97. doi: https://doi.org/10.1093/nar/gkw377
36. National Toxicology Program. DrugMatrix. 2010. doi: https://doi.org/ https://ntp.niehs.nih.gov/drugmatrix/index.html
